# Comparative glycosylation mapping of plasma-derived and recombinant human factor VIII

**DOI:** 10.1371/journal.pone.0233576

**Published:** 2020-05-22

**Authors:** Jingyao Qu, Cheng Ma, Xiao-Qian Xu, Min Xiao, Junping Zhang, Dong Li, Ding Liu, Barbara A. Konkle, Carol H. Miao, Lei Li, Weidong Xiao

**Affiliations:** 1 National Glycoengineering Research Center, Microbial Technology Institute, Shandong University, Qingdao, Shandong, China; 2 Department of Chemistry, Georgia State University, Atlanta, GA, United States of America; 3 Department of Hematology, Shanghai Jiaotong University Affiliated Shanghai General Hospital, Shanghai, China; 4 Sol Sherry Thrombosis Research Center, Temple University, Philadelphia, PA, United States of America; 5 Department of Clinical Laboratory, Shanghai Tongji Hospital, Tongji University School of Medicine, Shanghai, China; 6 Bloodworks Northwest, Seattle, WA, United States of America; 7 University of Washington, Seattle, WA, United States of America; 8 Center for Immunity and Immunotherapies, Seattle Children’s Research Institute, Seattle, WA, United States of America; University of Pennsylvania Perelman School of Medicine, UNITED STATES

## Abstract

Human coagulation factor VIII (FVIII) is a key co-factor in the clotting cascade, the deficiency of which leads to Hemophilia A. Human plasma-derived (pdFVIII) and recombinant FVIII (rFVIII) had been used as effective products to prevent and treat bleeding episodes. Both FVIII products share identical amino acid sequences and appear to be equivalent as of clinical efficiency. However, systemic reviews found an increased risk of neutralizing antibody (or inhibitor) development with recombinant products. FVIII is a highly glycosylated protein, and its glycosylation pattern is specific to host cells and environments. The roles of glycosylation in immune responses toward pdFVIII and rFVIII are yet to be defined. Herein, we systemically profiled *N*- and *O*-glycomes of pdFVIII and rFVIII using a mass spectrometry-based glycoproteomic strategy. A total of 110 site-specific *N*-glycopeptides consisting of 61 *N*-glycoforms were identified quantitatively from rFVIII and pdFVIII. Additionally, 31 *O*-glycoforms were identified on 23 peptides from rFVIII and pdFVIII. A comprehensive comparison of their site-specific glycan profiles revealed distinct differences between the glycosylation of pdFVIII and rFVIII.

## Introduction

Human coagulation factor VIII (FVIII) is a heavily glycosylated plasma protein consisting of six domains (A1, A2, B, A3, C1, and C2) along with a 19 amino acid signal peptide. [1] The deficiency of active FVIII leads to hemophilia A, one of the most common bleeding disorders. [2, 3] Under physiological conditions, FVIII forms a stable complex with von Willebrand factor (VWF) in the circulation, with a half-life of 12–18 hours. Upon activation by thrombin to remove the large B domain in the event of blood vessel damage, FVIII is converted into FVIIIa, which is then complexed with FIXa to activate FXa and initiate the clot formation. [4, 5] Patients with severe hemophilia require repeated infusions of plasma-derived FVIII (pdFVIII) or recombinant FVIII (rFVIII) to prevent and treat bleeding. Despite progresses made in developing various FVIII products, a frequent complication is the development of neutralizing alloantibodies (inhibitors) against FVIII. [6, 7] Once inhibitors develop in those patients, the regular dose of FVIII is no longer effective, administration of high doses (100–200 units/kg/day) for a prolonged period of time is often necessary to induce tolerance, named immune tolerance induction (ITI). An ongoing controversy in the field is whether treatments with plasma-derived products, particularly those containing VWF, are associated with less inhibitor development than those treated with recombinant ones. [8–11] Recently, a randomized trial of FVIII and neutralizing antibody in previously untreated hemophilia A patients concluded an overall inhibitor formation rate of 26.8% among patients treated with pdFVIII (contains VWF), but a much higher rate of 44.5% among those treated with rFVIII. [12] A possible explanation for this phenomenon is that VWF in complex with pdFVIII masks critical FVIII epitopes thus reduces its immunogenicity. [9, 13] Alternatively, it might result from different post-translational modifications (especially glycosylation) between pdFVIII and rFVIII that derived from various cell lines, as numerous reports had suggested that glycosylation variations affect the stability, immunogenicity, pharmacokinetics, and pharmacodynamics of glycoprotein biopharmaceuticals. [14–17] This is evidenced by a recent report that baby hamster kidney (BHK) cell-derived rFVIII (Kongenate FS) elicited a stronger immune response and exhibited accelerated clearance from circulation compared to Chinese hamster ovary (CHO) cell-derived rFVIII (Xyntha that is B-domain deleted and Advate) in hemophilia A mouse models. [18] The authors performed *N*-glycosylation profiling, revealed significant *N*-glycome differences between BHK and CHO cell-derived products. [18] Another most recent observation is that a rFVIII (Kovaltry) with higher levels of *N*-glycan branching and sialylation has an improved pharmacokinetic profile than other rFVIII products (Kogenate FS and Advate). [19] The field continues to reveal the functional roles of FVIII glycosylation and to understand the underlying mechanisms of inhibitor development.

We sought to identify possible inhibitor epitopes on FVIII related to glycans or glycopeptides and study the functional roles of site-specific glycosylation in inhibitor development. Such research activities rely on a comprehensive understanding of glycosylation patterns of both pdFVIII and rFVIII. The first report associated with FVIII glycosylation was in 1992, where Hironaka and coworkers chemically released *N*-glycans of pdFVIII purified from blood group A donors and rFVIII produced in BHK cells. [20] Glycosidase treatment and methylation analysis revealed that both proteins contain mainly high-mannose and bi-, tri-, and tetra-antennary complex *N*-glycans. Site-specific *N*-glycan heterogeneity and occupancy of a recombinant FVIII expressed in CHO cells were later reported in 1997, using liquid chromatography-electrospray ionization mass spectrometric (LC-ESI-MS) analysis combined with selected ion monitoring (SIM). [21] Subsequently, Thim and coworkers reported the production of a B domain partially truncated FVIII produced in CHO cells, N8. A total of 4 *N*-glycosites were identified from A1, A3, and C1 domains by matrix-assisted laser desorption/ionization-MS (MALDI-MS) analysis, and glycoforms on these sites are mainly sialylated bi-antennary structures. [22] Recent advances in mass spectrometry (MS) instrumentation and methodology have made in-depth analyses of protein glycosylation more accessible and practical, revealing many more details, e.g., specific glycoforms and linkage information. Accordingly, the *N*-glycosylation of pdFVIII and several rFVIII products were profiled by various MS approaches in detail. [18, 19, 23, 24] For example, Kannicht and coworkers site-specifically profiled the *N*-glycome of A and C domains of a rFVIII derived from a human cell line using LC-MS/MS, revealed that *N*-glycans on N41 and N1810 mainly contain bi-antennary complex structures, whereas N2118 was solely occupied by high-mannose *N*-glycans. [24] Structures on N239 were most diverse, with high-mannose, bi-antennary complex and hybrid types *N*-glycans. These results were confirmed by a most recent report, where an in-depth comparison of *N*-glycosylation was performed using a combination of MALDI-MS/MS, GC-MS, and UPLC-UV-MS analysis. [23] Besides the 4 *N*-glycosites on A and C domains, the authors were able to identify 14 *N*-glycosites on the B domain of pdFVIII and two rFVIII derived from BHK and CHO cell lines.

Nevertheless, the *N*-glycan profile of FVIII seems to be cell line-dependent, and relevant quantitative information is rarely reported. Additionally, comprehensive profiling of FVIII *O*-glycome is still missing, regardless of the finding that the B-domain of pdFVIII was highly *O*-glycosylated. [21, 25, 26] In this study, we performed the comprehensive site-specific *N*- and *O*-glycan profiling of full-length plasma-derived FVIII from humans (pdFVIII-f) and a recombinant FVIII (Kogenate FS, rFVIII-K), as well as quantitative glycoform analysis of each *N*-glycosite. A total of 61 *N*-glycoforms were identified from 13 *N*-glycosites, and 31 *O*-glycoforms were identified on 23 peptides A2, B, C1, and C2 domains. Comparative glycan analysis revealed disparate *N*- and *O*-glycan patterns between the two FVIII products.

## Materials and methods

### Material and chemicals

Recombinant factor VIII (Kogenate FS) was from Bayer AG. The FVIII enriched cryoprecipitate was obtained from Shanghai Lai Shi Blood Products Co., Ltd (Shanghai, China). PNGase F was acquired from New England Biolabs (Ipswich, MA). Sequencing-grade porcine trypsin was purchased from Promega (Madison, WI); ZIC-HILIC resin was acquired from Merck (Darmstadt, Germany). Deionized water was produced by a Milli-Q A10 system from Millipore (Bedford, MA). HPLC grade acetonitrile (ACN) was purchased from J. T. Baker Inc. Iodoacetamide (IAA), and DTT were purchased from ACROS ORGANIC (Geel, Belgium). 3M Empore C8 disk was obtained from 3M Bioanalytical Technologies (St. Paul, MN). Filter YM-30 (30 kD) and zip-tip C18 were purchased from Millipore. LCMS-grade formic acid (FA) and other chemicals were purchased from Sigma-Aldrich (St. Louis, MO).

### Purification of plasma-derived factor VIII

The FVIII enriched cryoprecipitate was dissolved at a final FVIII activity of about 10 U/ml. Prothrombin complex related proteins were absorbed with Al(OH)_3_ solution. After centrifugation, the resulted supernatant was treated with 1% TNBP and 1% Triton X-l00 at 30°C for 4 hours for virus inactivation. The solution was then subjected to buffer exchange by ultra-filtration (0.5 M NaCl, 0.25 M CaCl_2_, and 20 mM Tris-HCl pH 6.5). The obtained solution was then loaded on a human FVIII affinity column (VIII select, GE). The resulting FVIII enriched eluent was subjected to Q-FF ionic exchange (GE) column subsequently. Then the product was polished by SP (GE) and gel chromatography. The subsequent product was buffer-exchanged by ultrafiltration with 0.15 M NaCl, 100 μg/ml Tween-80, 20 mM histidine, 5 mM CaCl_2_ and 5% trehalose. After the final sterile filtration and filling step, the purified pdFVIII product (S2 Fig) was lyophilized and stored at 2–8°C.

### Trypsin digestion

Approximately 200 μg FVIII was subjected to the filter-aided sample preparation (FASP) procedure as reported [27]. In brief, 200 μg FVIII were mixed with 50 μL of lysis buffer (20 mM Tris-HCl, 4% (v/v) SDS, 100 mM DTT, pH 7.6), and incubated for 5 mins at 95°C. After mixing with 200 μL of UA solution (8 M urea in 0.1 M Tris/HCl, pH 8.5), the sample was loaded into a 30 kDa Microcon filtration device and centrifuged at 13,000 g until the volume was reduced to less than 10 μL. The concentrate was washed twice in the device with 200 μL of UA solution. After centrifugation, the concentrate was mixed with 100 μL of 50 mM IAA in the UA solution and incubated in the dark at room temperature (RT) for 30 mins, followed by brief centrifugation for 20 mins. Then, the protein concentrate was washed two more times against the UA solution. The so yielded sample was diluted twice with 100 μL of 40 mM NH_4_HCO_3_ for digestion. Sequencing grade trypsin was mixed with the sample at an enzyme to protein ratio of 1:50 for overnight digestion at 37°C. Finally, digested peptides were transferred into a filtration device and subjected to centrifugation, and washed with 50 μL of 0.5 M NaCl for 20 mins for 6 times. The concentration of peptides was determined by a Thermo Nanodrop UV-spectrometer, applying an extinction coefficient of 1.1 for 0.1% (g/L) solution at 214 nm.

### *N*-glycan removal and ^18^O labeling

Fifty micrograms of tryptic digested peptides were dried under vacuum, dissolved in 50 μL of 50 mM ammonium bicarbonate prepared from ^18^O water (97 atom % ^18^O, Sigma-Aldrich). Deglycosylation was performed by 250U of PNGase F (New England Biolabs) in 37°C water bath for 8 hours. The reaction mixture was then dried under vacuum, desalted with Zip-tip according to the protocol, and subjected to LC-MS/MS analysis. CID data from the PNGase F-in-H_2_^18^O treatment is processed with Proteome Discoverer 1.4 (Thermo Fisher Scientific). Peptide fragments are matched against the FVIII protein sequence (UniProtKB entry P00451), where the carboxymethyl on Cys is used as static modification, oxidation of Met and ^18^O labeling of Asn (Δm = 2.9848) as dynamic modification.

### Glycopeptides enrichment

Approximately 100 μg of digested peptides were added into 20 mg ZIC-HILIC resin and enriched as we previously described [28]. Briefly, a piece of C8 disk was put into a 200 μL pipette tip, and 3 mg ZIC-HILIC resin that suspended in 100 μL acetonitrile was loaded into the tip, followed by equilibration with binding buffer (80% ACN, 5% FA). In-solution digested peptides were re-dissolved in the binding buffer, and loaded into the tip. The ZIC-HILIC tip was washed with 100 μL binding buffer for six times and bounded peptides were eluted with 80 μL elution buffer (99.5% H_2_O, 0.5% FA) for three times. The concentration of ZIC-HILIC enriched peptides was determined by UV-spectrometry. Desalted peptides were stored at -80°C until analysis by LC-MS/MS.

### LC-MS analysis

Experiments were performed on an LTQ-Orbitrap Elite mass spectrometer (Thermo Fisher) equipped with EASY-spray source and nano-LC UltiMate 3000 high-performance liquid chromatography system (Thermo Fisher). EASY-Spray PepMap C18 Columns (15 cm; particle size, 3 μm; pore size, 100 Å; Thermo Fisher) were used for separation. Separation was achieved with linear gradient elution from 3% to 40% solvent B for 30 min at a flow rate of 300 nL/min (mobile phase A, 2% ACN, 98% H_2_O, 0.1% FA; mobile phase B, 80% ACN, 20% H_2_O, 0.1% FA). The LTQ-Orbitrap Elite mass spectrometer was operated in data-dependent mode. A full-scan survey MS data (m/z range from 375 to 1,500; automatic gain control target, 10^6^ ions; resolution at m/z 400, 60,000; maximum ion accumulation time, 50 ms) was acquired by the Orbitrap analyzer, and 10 most intense ions were fragmented by HCD in the octopole collision cell. HCD fragment ion spectra were acquired in the Orbitrap analyzer with a resolution of 15,000 at m/z 400. Each experiment was performed in duplicates.

### Data interpretation

*N*-acetylhexosamine (HexNAc) is a common sugar residue in almost all glycopeptides. The bond of glycosidic linkages within oligosaccharides is easier to break than the amide bond between the innermost GlcNAc and Asn via HCD fragmentation. Therefore, it is the last bond to break when an *N*-glycopeptide enters the collision cell during MS/MS analysis before the peptide backbone starts to fall apart. Also, *N*-glycan has a conserved core structure. Combining this knowledge with bioinformatics tools (e.g., PeptideMass in ExPASY), glycan masses are calculated by parent ion and peptide mass. Structures are predicted by database search and fragment pattern analysis. HCD spectra of glycopeptides were selected manually by the presence of HexNAc fragment ions of 126.0549, 138.0549, 144.0655, 168.0760 and 204.0866. Theoretical masses of all possible peptides were calculated by PeptideMass [29]. Due to the nature of covalent linkage in *N*- and *O*- glycosylation, [peptide+GlcNAc]^n+^ and [peptide]^n+^ masses can be used to cross-reference selected spectra for rough identification of peptide sequences, respectively, thanks to the high mass accuracy of Orbitrap detector. Given the relative simplicity compared to *N*-glycopeptide fragmentations, peptide ID of *O*-glycopeptides are easier to be further confirmed by matching b and y ions. The glycan mass of individual glycopeptide can then be calculated by subtracting peptide mass from parent ion mass and adding one water molecule (18.0106). The resulting masses were then searched against CarbBank with free reducing end and no derivatization using profiler function from GlycoWorkbench [30, 31]. Possible structure hits can be narrowed down by the sequential addition of monosaccharide residues to the peptide. For example, the presence of [peptide+GlcNAc+Fuc]^n+^ ion is direct evidence of core-fucosylated *N*-glycopeptide.

## Results

### Sample preparation and data collection

For site-specific glycoanalysis of a large glycoprotein like FVIII, results from open software had been notoriously inaccurate. [32] In this study, we combined manual and software analysis with the highly accurate HCD fragmentation MS strategy. The normalized collision energy (NCE) is set at 27 to generate good glycopeptide fragment ions based on our previous reports. [33] Under this condition, the HexNAc moiety from *N*-, *O*-glycopeptides were fragmented into a series of diagnostic ions of 126.0549, 138.0549, 144.0655, 168.0760 and 204.0866. Some oligosaccharide fragments were also observable, such as Hex-HexNAc (366.1395), Hex-Hex-HexNAc (528.1923), and Neu5Ac-Hex-HexNAc (675.2455). Meanwhile, in most cases, peptide backbones would not dissociate, leaving a strong peptide (N)-HexNAc (Y1 ions) signal in *N*-glycopeptides or a peptide peak in *O*-glycopeptides as a clue for the peptide identity. Peptide fragmentation peaks were observed in *O*-glycopeptides with short sugar sequences in low signal intensities, further facilitating peptide identification. The analytical workflow is shown in S1 Fig. Briefly, pdFVIII-f and rFVIII-K were denatured, digested by trypsin, and dried under vacuum. For *N*-glycosylation site analysis, PNGase F digestion was performed in H_2_^18^O. Deglycosylated peptides were subjected to LC-MS/MS analysis using a C18 nano-column and collision-induced dissociation (CID). Home-made hydrophilic interaction liquid chromatography-solid phase extraction (HILIC-SPE) cartridge was applied for intact *N*- and *O*-glycopeptide enrichment. Higher energy collisional dissociation (HCD) was selected to analyze glycopeptide. The HCD technique surmounted the problems of low mass cutoff of ion trap fragmentation, and dramatically improved the quality of MS/MS spectra because of the high accuracy at both the precursor mass and fragment levels. [34]

### *N*-Glycosite occupancy and relative abundance of *N*-glycans in pdFVIII-f and rFVIII-K

**Fig 1:**
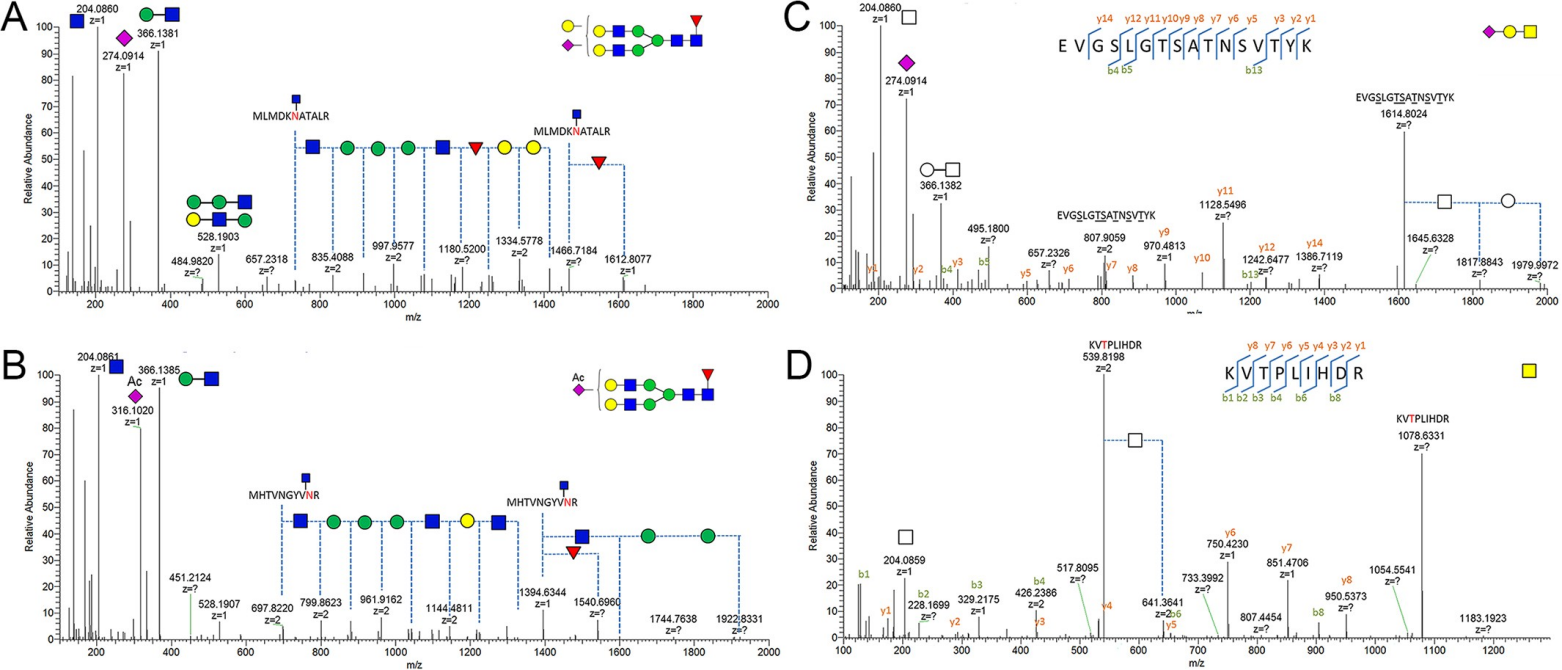
Examples of *N*-glycan identification analysis (A, B) and glycan identification and peptide recognition of *O*-glycopeptides (C, D).

From a typical one-hour LC-HCD-MS/MS analysis of enriched glycopeptides, roughly 400 glycan-fragment-containing MS/MS spectra were collected, from which 110 site-specific *N*-glycopeptides with 61 glycoforms were quantitatively identified from pdFVIII-f and rFVIII-K (Table 1 and S1 Table). Among these, 50 glycoforms were identified from 9 *N*-glycosites on rFVIII-K, and 36 *N*-glycoforms were identified from 10 sites on pdFVIII-f (S1 Table). Examples of tandem MS annotation of *N*-glycopeptides are illustrated in Fig 1A & 1B. Site occupancy estimated by the ratio of regular and ^18^O labeled Asn-containing peptides were summarized in S1 Table. While most identified sites are occupied over 50%, one site from rFVIII-K (SHSIPQAN_1384_R) and two sites from pdFVIII-f (IQN_784_VSSSDLLMLLR and EDFDIYDEDEN_1685_QSPR) have less than 20% occupancy. Most significantly, N1066 was 100% occupied in rFVIII-K, while no glycan was observed on that of pdFVIII-f. In contrast, pdFVIII-f has an *N*-glycan occupancy of 50% on N757, while the site rFVIII-K is not glycosylated. How the cells control the non-template driven glycosylation pathway to achieve such selectivity is beyond our understanding.

**Table 1:** *N*- (x-N) and *O*-linked (x-O) glycoforms identified on FVIII by LC-ESI-MS/MS.

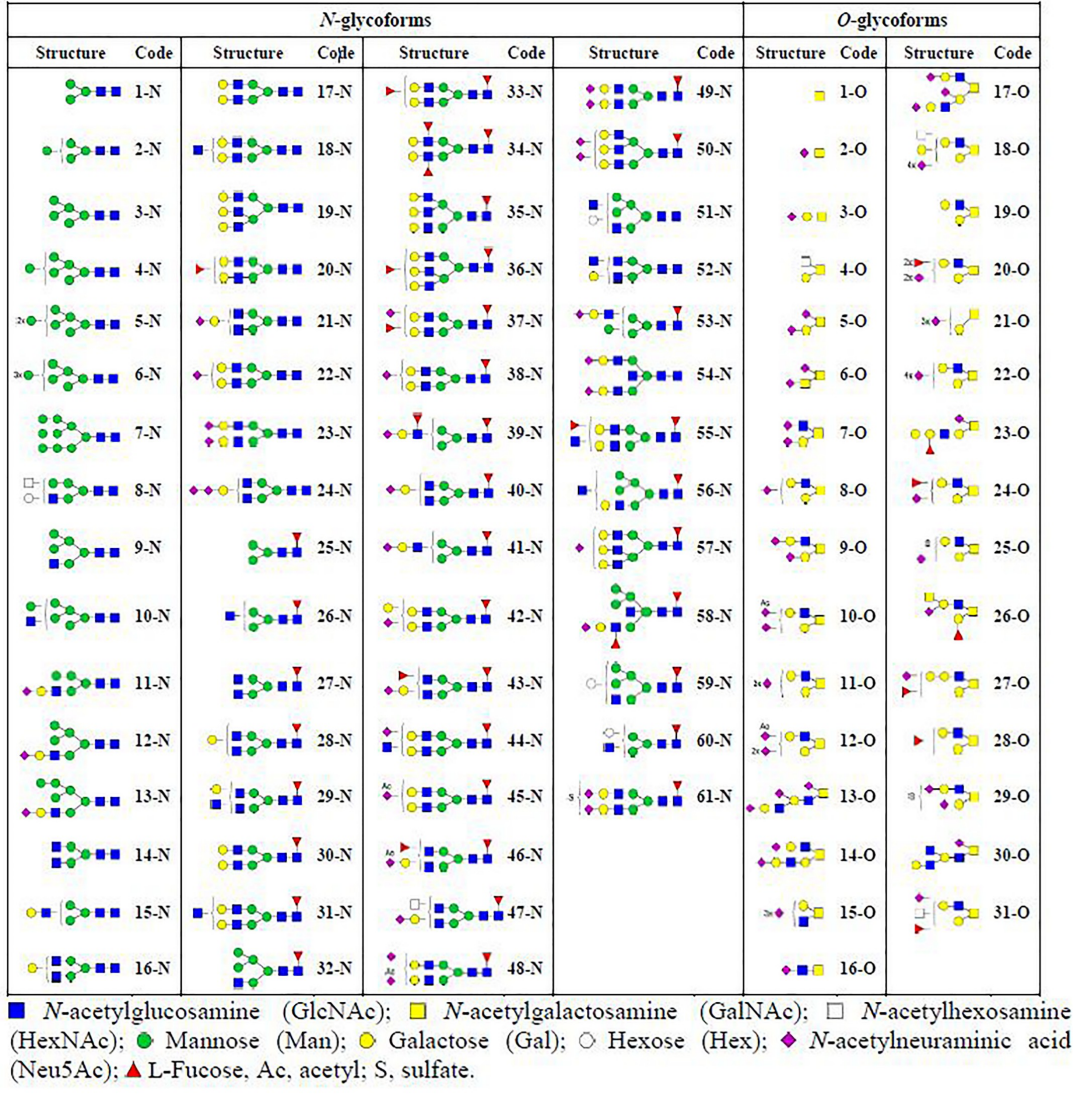

**Fig 2:**
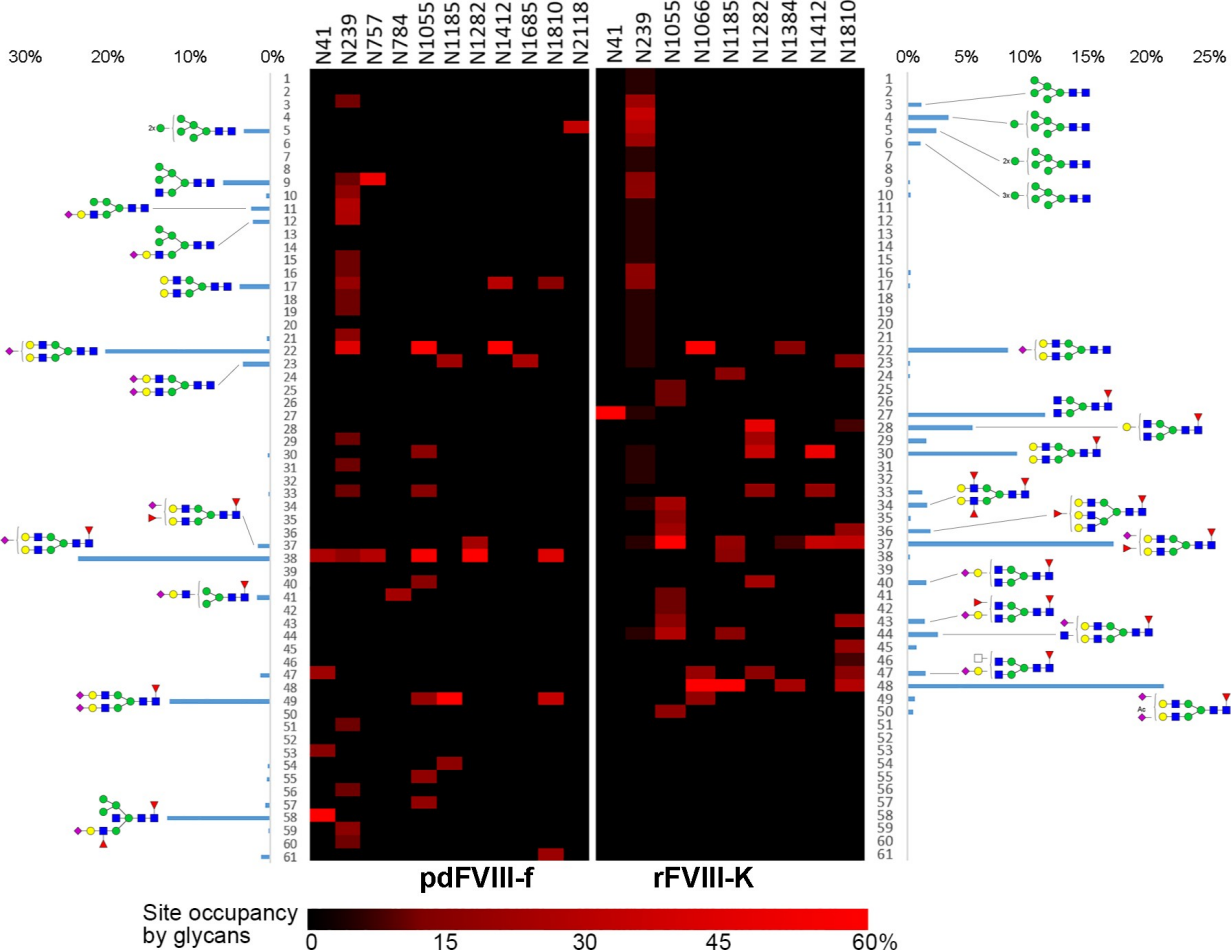
*N*-glycan comparison between pdFVIII-f and rFVIII-K. Total relative abundance of 61 *N*-glycan compositions are represented by columns, site-specific relative abundance is presented in heat map.

To estimate relative abundance, the peak area percentage of each glycopeptide (extracted by XIC regarding the accurate mass at the corresponding retention time) was calculated from the sum of all glycopeptides at each site. The total relative abundance of each glycoform is estimated from the site occupancies and relative abundance at each site. Fig 2 illustrates the overall occupancy of glycans on each *N*-glycosite of both rFVIII-K and pdFVIII-f. The majority of most abundant *N*-glycoforms are composed of the bi-antennary H5N4 backbone (Gal_2_GlcNAc_2_Man_3_GlcNAc_2_, 17-N), with one or two sialic acid residues (S) and the presence or absence of core-fucosylation (F). The most abundant *N*-glycoform on pdFVIII-f is H5N4S1F1 (38-N, 23%). This core-fucosylated *N*-glycan is identified on 6 out of 11 *N*-glycosites. The second most abundant glycoform is its non-core-fucosylated form (H5N4S1, 22-N, 20%), which is distributed among N239, N1055 and N1412. On the other hand, the top 2 abundant glycoforms found on rFVIII-K are mono-acetylated H5N4S2F1 (48-N, 21%) and H5N4S1F2 (37-N, 17%).

### Site-specific *N*-glycome comparison of pdFVIII-f and rFVIII-K

**Fig 3:**
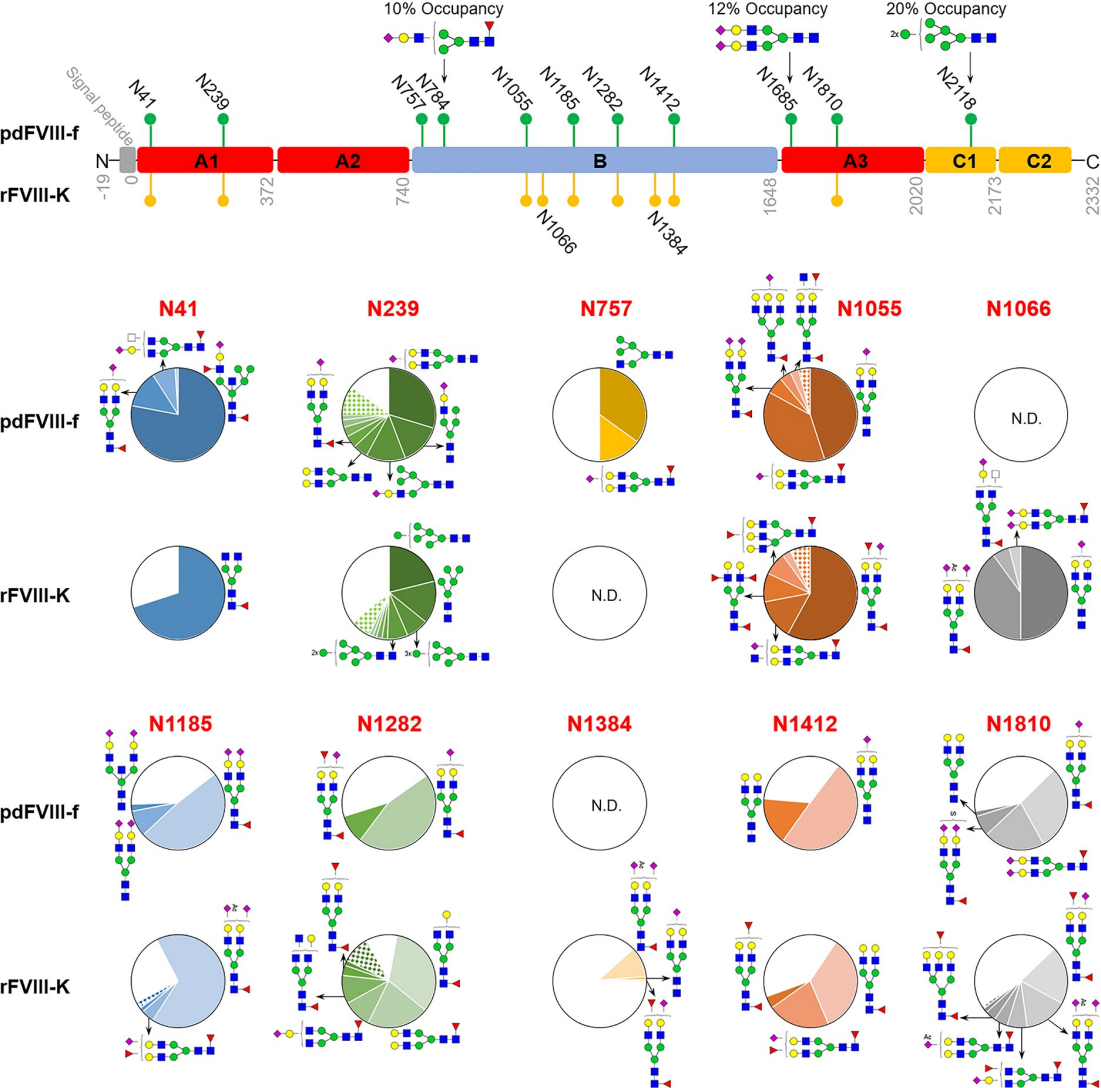
Comparison of *N*-glycosite localization and glycoform abundance between rFVIII-K and pdFVIII-f. The sites identified in rFVIII-K were labeled orange while those in pdFVIII-f in green. Site-specific *N*-glycosylation analysis with occupancy and relative abundance were shown in pie charts. White pie, not glycosylated; colored pie, the abundance of each glycoform; patterned pie, the total abundance of remaining glycoforms on this site.

Site-specific *N*-glycome comparison with occupancy and abundance is shown in Fig 3. For both pdFVIII-f and rFVIII-K, 2, 0, and 1 *N*-glycosites were identified from the A1, A2, and A3 domains respectively, consistent with previous reports. [18, 21, 23, 24] Like recently reported,[23, 24] core-fucosylated *N*-glycans were predominant on N41 and N1810 of both FVIIIs (Fig 3), specifically, N1810 were mainly occupied by core-fucosylated bi-antennary complex *N*-glycans. Worth noting that a core-fucosylated hybrid *N*-glycan H6N4S1F2 (58-N) accounts for 78% glycoforms on N41 of pdFVIII-f, different from a recent report where core-fucosylated bi-antennary complex structures were dominant on this site of pdFVIII. [23] An explanation would be pdFVIII products were obtained from different commercial resources. Such results again demonstrated that glycosylation of FVIII is cell line- and environment-dependent. Additionally, N41 of rFVIII-K was predominantly occupied by an agalacosylated *N*-glycans (H3N4F1, 27-N), unlike previous reports that galactosylated *N*-glycans are among top glycoforms on this site of other rFVIII products. [18, 24] A major difference between the glycosylation of the two FVIIIs is glycoforms on N239, with complex and hybrid structures dominant on pdFVIII-f, while high-mannose structures dominant on rFVIII-K (Fig 3). Specifically, the top 5 high-mannose glycans (3-N, 4-N, 5-N, 6-N, and 7-N) account for around 80% relative abundance out of the total 31-identified glycoforms on this site of rFVIII-K (S1 Table). The most abundant glycoform on N239 of pdFVIII-f is non-core-fucosylated bi-antennary *N*-glycan H5N4S1 (22-N), same as the recent report on another commercial pdFVIII. [23] Differently, high-mannose *N*-glycans were also found on the pdFVIII,[23] but not on pdFVIII-f (Fig 3). Lastly, we identified a new *N*-glycosite on the A3 domain of pdFVIII-f, N1685, even with low occupancy at 12%. Only one glycoform H5N4S2 (23-N) was observed on this site. No glycans was found on the same site of rFVIII-K.

The C1 domain of pdFVIII-f contains another *N*-glycosite N2118, which occupied solely by a high-mannose *N*-glycan (5-N). Other reports also confirmed that this site of pdFVIII and various rFVIIIs were predominantly occupied by high-mannose glycans,[23, 24] which is essential for the secretion of FVIII proteins. [35] No *N*-glycosylation sequon exists in the C2 domain.

Multiple *N*-glycosylation sites were identified from the B domain of both pdFVIII and rFVIII,[21, 23], but detailed quantitative information on each site has not been reported. In this study, *N*-glycans were observed on 6 N-X-T/S sequons in the B domain of pdFVIII-f (N757, N784, N1055, N1185, N1282, N1412), and 6 sequons in rFVIII-K (N1055, N1066, N1185, N1282, N1384, N1412) (Fig 3). These sites mainly contain core-fucosylated bi-antennary *N*-glycans, consistent with a recent report. [23] The most significant difference between pdFVIII-f and rFVIII-K is the glycosylation on sites N757 and N1066. N757 of pdFVIII-f was 50% occupied by hybrid glycan H4N3 (9-N) and H5N4S1F1 (38-N), whereas no glycan was detected on that of rFVIII-K. On the other hand, N1066 of rFVIII-K was 100% occupied by 4 sialylated bi-antennary glycans, but the site of pdFVIII-f is not glycosylated (Fig 3). Different *N*-glycan patterns were also observed on the 4 shared glycosites (N1055, N1185, N1282, N1412) in the B domain of both pdFVIII-f and rFVIII-K (Fig 3). For example, N1412 of pdFVIII-f contained only non-core-fucosylated complex glycans, while all glycans on that of rFVIII-K were all core-fucosylated. These glycosylation disparities between pdFVIII-f and rFVIII-K could result in changes in immunogenicity. [18]

### *O*-Glycome comparison of pdFVIII-f and rFVIII-K

**Fig 4:**
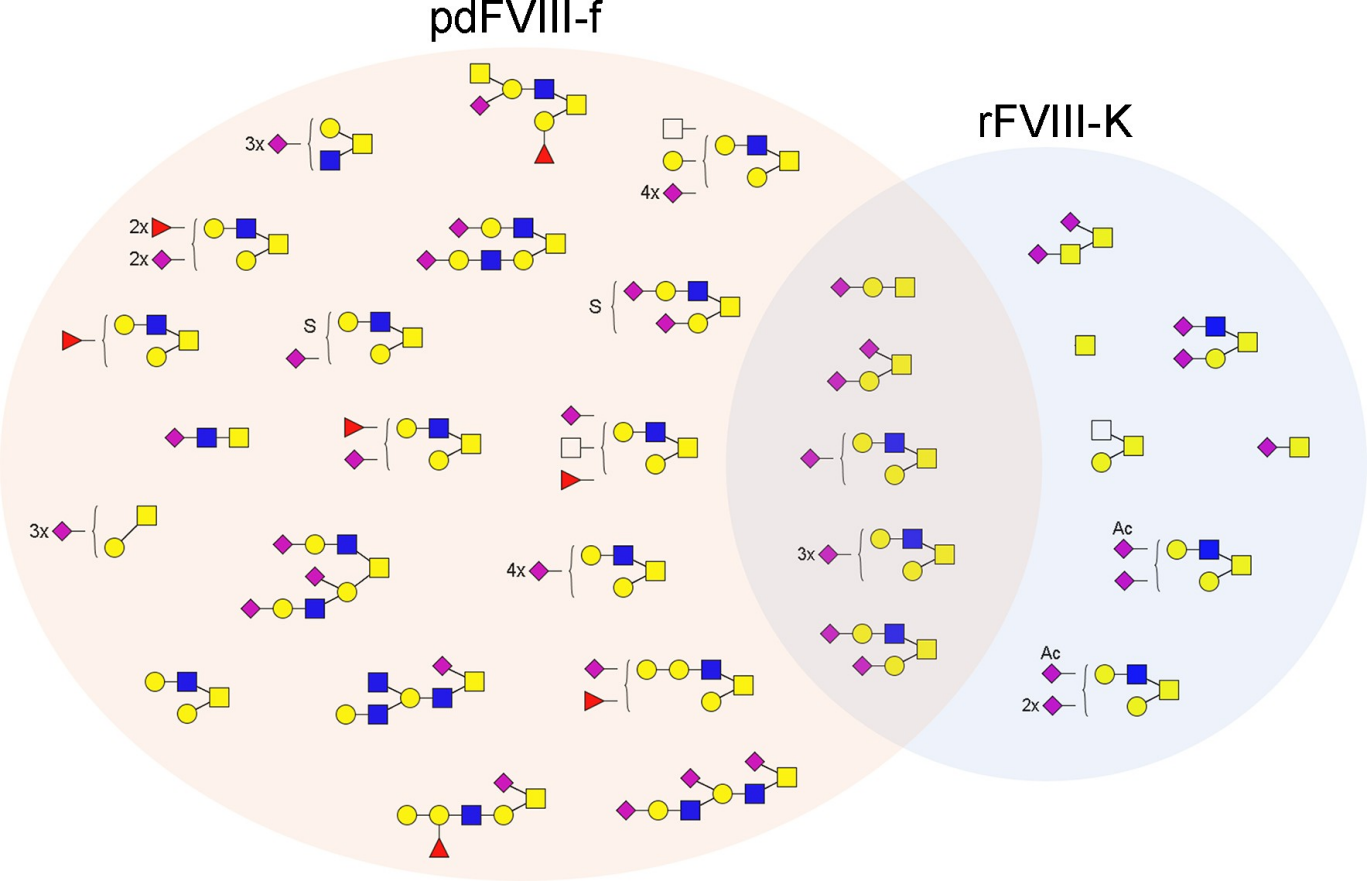
*O*-glycoforms identified from pdFVIII-f and rFVIII-K.

A total of 31 *O*-glycoforms were identified from 23 peptides in pdFVIII-f and rFVIII-K (Table 1, Table 2). The linkages between *O*-GalNAc and Serine or Threonine are chemically similar to glycosidic bonds if not identical. Therefore, strong bare peptide ions were observed during MS/MS analysis. Using a similar approach as that for *N*-glycosylation profiling, 34 unique *O*-glycopeptides were identified from rFVIII-K with 12 *O*-glycoforms on 18 peptides, while 37 *O*-glycopeptides were identified from pdFVIII-f with 24 *O*-glycoforms on only 9 peptides (Table 2). The two FVIIIs share 5 *O*-glycoforms (Fig 4). Most glycoforms are Core 2 structures that contain a core trisaccharide GlcNAc-β1,6-(Gal-β1,3-)GalNAc. Examples of tandem MS annotation of *O*-glycopeptides are illustrated in Fig 1C & 1D. The majority of identified *O*-glycopeptides were located in the B domain, consistent with previous observations. [21, 25, 26] Additionally, one *O*-glycopeptide was identified from the A2 domain of pdFVIII-f, and two was identified from C domains of rFVIII-K (Table 2). In terms of glycoform diversity on different glycosites, peptide T_769_DPWFAHRTPMPK and Q_796_SPTPHGLSLSDLQEAK of pdFVIII-f contained 12 and 9 *O*-glycoforms of 24 identified ones, respectively, representing the most diversified O-glycosites. Another peptide in the B domain, L_863_GTTAATELK, contained 7 glycoforms. Glycosylation on the same peptide was found to be most diversified in rFVIII-K, with 9 out of 12 identified *O*-glycoforms (Table 2).

**Table 2:** *O*-glycan localization and glycoforms identified on pdFVIII-f and rFVIII-K by LC-ESI-MS/MS.

Peptide sequence	*O*-Glycoforms	
	pdFVIII-f	rFVIII-K
S_409_QYLNNGPQRIGR	20	
S_741_FSQNSR		3, 5
T_769_DPWFAHRTPMPK	5, 8, 9, 14, 16, 19, 21, 23, 24, 25, 26, 29	
Q_796_SPTPHGLSLSDLQEAK	8, 9, 11, 15, 24, 27, 28, 30, 31	8
L_863_GTTAATELKK	9, 11, 13, 15, 17, 18, 22	3, 4, 5, 7, 8, 9, 10, 11, 12
L_947_LESGLMNSQESSWGKNVSSTESGR		3
K_1041_VTPLIHDR		1, 2, 5
K_1081_EGPIPPDAQNPDMSFFK		3
G_1155_EFTKDVGLK		5
N_1173_LFLTNLDNLHENNTHNQEK		2
K_1263_HTAHFSKK		5
Q_1286_IVEKYACTTR	5	3, 5, 6
I_1297_SPNTSQQNFVTQR		5
Q_1317_FRLPLEETELEK		5
H_1346_LTPSTLTQIDYNEK	3, 5	
E_1458_VGSLGTSATNSVTYK		3, 5, 9, 11
K_1474_VENTVLPKPDLPK		5
V_1549_ATESSAKTPSK		5
L_1561_LDPLAWDNHYGTQIPKEEWK	9	5
L_1635_CSQNPPVLKR	3, 5, 9	
Q_1629_GRTERLCSQNPPVLKR	8	
T_2086_QGARQK		7
V_2280_KVFQGNQDSFTPVVNSLDPPLLTR		3

Note that for identified glycopeptides containing multiple Ser or Thr (underlined), current method could not specify the exact glycosite.

The most widely distributed *O*-glycan in rFVIII-K is the Core 1 tetrasaccharide disialyl-T antigen (5-O), observed on 12 peptides. The structure was also among top distributed *O*-glycans in pdFVIII-f, found on 5 of the 9 identified *O*-glycosylated peptides. A complex Core 2 hexasaccharide, 9-O, is the most widely distributed glycoform in pdFVIII-f, observed on 6 peptides (Table 2). An apparent difference between the *O*-glycosylation of the two FVIIIs is that detected glycan structures on pdFVIII-f are far more complicated than that on rFVIII-K (Fig 4). While 15 of 24 glycoforms on pdFVIII-f are hexasaccharide or longer, with the most complexed structure 18-O as a decasaccharide, rFVIII-K only contained 4 complex *O*-glycoforms with 2 hexasaccharide (9-O, 10-O) and 2 heptasacharide (11-O, 12-O). Additionally, complexed glycans are more abundant in pdFVIII-f. For example, 6 of the 9 peptides from pdFVIII-f was occupied by hexasaccharide or larger glycoforms. On the other hand, among the 18 identified peptides from rFVIII-K, only 4 (Q_796_SPTPHGLSLSDLQEAK, L_863_GTTAATELKK, E_1458_VGSLGTSATNSVTYK, and T_2086_QGARQK) contain *O*-glycans with pentasaccharide or larger. Another notable difference between the two FVIIIs is that fucosylation and sulfation were observed only on pdFVIII-f, whereas acetylated Neu5Ac was identified only on rFVIII-K. Given the complicity of *O*-glycans and multiple Ser/Thr residues on many of the identified *O*-glycopeptides, further effort is required to fully characterize the *O*-glycosylation of FVIII.

## Discussion

Glycosylation is important for the stability, pharmacokinetics, and immunogenicity of recombinant glycoprotein biopharmaceuticals. As a result of non-template driven biosynthesis, glycans are inherently complex and diverse. Various glycoforms were frequently observed on even a single glycosites. Additionally, different organisms usually possess different glycosylation pathways as they may express a varied repertoire of glyco-enzymes and transporters, yielding disparate glycan patterns on expressed glycoproteins. It is thus understandable that host cell lines used to produce glycoprotein therapeutics have strong influences on their glycosylation profiles. [36] To date, three cell lines have been used to express rFVIII, including CHO cells, BHK cells, and human embryonic kidney (HEK) cells. All these cell lines are mammalian expression platforms, glycan pattern differences on proteins expressed in these platforms were well summarized by Justin Bryan Goh and Say Kong Ng. [36] Taking recombinant factor VII (rFVII) as an example, the primary *N*-glycoform on CHO- and BHK-derived FVII was reported to be core-fucosylated-di-sialylated bi-antennary complex structures with, while HEK293-derived FVII was found to be most heterogeneous with core-fucosylated-agalactosylated bi-antennary complex structures. Plus, it was found that pdFVII contains the highest levels of sialylation, while HEK293-derived FVII contains the lowest levels of sialylation. Recently, Canis and coworkers reported an in-depth comparison of the *N*-glycosylation of pdFVIII and rFVIII derived from the three cell lines. [23] It was concluded that CHO cells produce complex *N*-glycans with an average number of antennae closest to pdFVIII, while BHK cells produce a higher number and HEK a lower number. In addition, rFVIII expressed in HEK cells exhibited lowest levels of sialylation (14 to 24%) and highest levels of fucosylation (13 to 38%), whereas pdFVIII had a much higher sialylation (67%) and lower fucosylation (10%). Sialyation ratios of BHK- and CHO-derived FVIII are similar to that of pdFVIII, but their fucosylation ratios are extremely low (3% for BHK cells, and less than 1% for CHO cells). Our results confirmed *N*-glycosylation differences between pdFVIII-f and rFVIII-K (derived from BHK cells), which are not only reflected from glycosites, but also glycoforms on them. For example, N757 and N784 were glycosylated in pdFVIII-f but not rFVIII-K, whereas N1066 and N1384 were glycosylated in rFVIII-K but not pdFVIII-f. Glycoforms on N41, N239 and N1412 of pdFVIII-f are also disparate from those on the sites of rFVIII-K (Fig 3). Note that overall *N*-glycan patterns of pdFVIII-f and rFVIII-K are similar to previously reported pdFVIII and BHK-derived rFVIII,[23] site-specific microheterogeneity were observed. For example, 58-N and 38-N are top 2 abundant glycoforms on N41 of pdFVIII-f identified in this study, whereas 38-N and 49-N are the most abundant ones on that of another commercial pdFVIII. [23] These results indicated that glycosylation can also be influenced by environmental factors, thus glycosylation had been used as one of the quality control factors in glycoprotein production. [37]

The most significant difference between pdFVIII-f and rFVIII-K is their *O*-glycosylation. While more *O*-glycosites were identified form rFVIII-K, the glycoforms on pdFVIII-f are far more complicated and diversified (Fig 4). Fucosylation and sulfation were only observed on glycans of pdFVIII-f, whereas acetylation was only observed on that of rFVIII-K. Additionally, rFVIII-K contain mainly under-glycosylated *O*-glycoforms, e.g., Tn (1-O) and STn (2-O) antigen. Such *O*-glycome difference and under-glycosylation might contribute to the immunogenicity of FVIII. Recombinant glycoprotein therapeutics produced in non-human mammalian cell lines are often modified with the well-known immunogenic form of sialic acid *N*-glycolylneuraminic acid (Neu5Gc), and Neu5Gc-specific antibodies can often be detected in human. [11] However, in this work, Neu5Gc was not observed in either rFVIII or pdFVIII, which is consistent with a previous report,[24] but inconsistent with the most recent report. [23] On the other hand, the immunogenic α-Gal epitope (Gal-α1,3-Gal) is observed (e.g., 41-N on MLMDKN_1055_ATALR), even though in limited amounts with less than 2% total glycopeptide estimated by peak area (S1 Table), which is consistent with the recent report. [23] All these epitopes could increase the immunogenicity and contribute to inhibitor development.

A recent study compared the processing of FVIII by dendritic cells from HA patients and healthy donors, and found that MHC-II proteins resent fewer peptide fragments when administered pdFVIII than full-length rFVIII. [38] It was proposed that biosynthesis of pdFVIII in human cells under physiological conditions could result in reduced heterogeneity and immunogenicity, and/or subtle conformational differences, in turn, may result in reduced FVIII proteolytic processing than rFVIII. Specifically, different glycosylation patterns could be the primary factor. [39] For example, it was concluded that a mannose receptor is involved in immune recognition of FVIII by antigen-presenting cells (APC), e.g., dendritic cells. [40] Our work and previous studies showed that N2118 at the C1 domain is primarily attached with high-mannose *N*-glycans in both pdFVIII and rFVIII,[23, 24] suggesting a mechanism for FVIII internalizations by APC via the mannose receptor. A major difference between the two FVIII analyzed in this study is disparate glycoform presented on N239. pdFVIII-f contains mainly complex structures with two hybrid ones (11-N, 12-N) on this site, whereas the site of rFVIII-K contains solely high-mannose type *N*-glycans. Such high levels of high-mannose glycoforms on N239 of rFVIII-K could be a major cause of higher immunogenicity of rFVIII-K than pdFVIII. [18] The Future efforts are required in such directions.

In summary, we systemically analyzed and compared the glycomes of rFVIII-K and pdVIII-f by using nano LC-MS/MS system functionalized with HCD fragmentation. A total of 61 *N*-glycan structures or isomers and 31 *O*-glycan compositions were identified. The results revealed a significant divergence between rFVIII and pdFVIII regarding both glycosites and glycoforms. Although the likely immunogenic epitope, Neu5Gc, was absent in both rFVIII and pdFVIII, other immunogenic glycopeptides that harbor the α-Gal or Tn/STn epitopes were identified in rFVIII. The glycome disparity and presence of immunogenic glycan epitopes may contribute to FVIII inhibitor development.

## Supporting information

S1 Fig Analytical workflow for glycosylation mapping of pdFVIII and rFVIII. (PDF)

S2 Fig SDS-PAGE of FVIII enriched cryoprecipitate and purified plasma-derived FVIII. (PDF)

S1 Table Site-specific *N*-glycosylation of pdFVIII-f and rFVIII-K identified by LC-ESI-MS/MS, occupancy of each site and percentage of each glycoform. (PDF)

S1 File Representative spectra of identified glycoforms. (PDF)
