# Supplementary figures and images for: Comparative glycosylation mapping of plasma-derived and recombinant human factor VIII
Source: PLoS One. 2020 May 22;15(5):e0233576. doi: 10.1371/journal.pone.0233576 (PMC7244179; doi:10.1371/journal.pone.0233576)

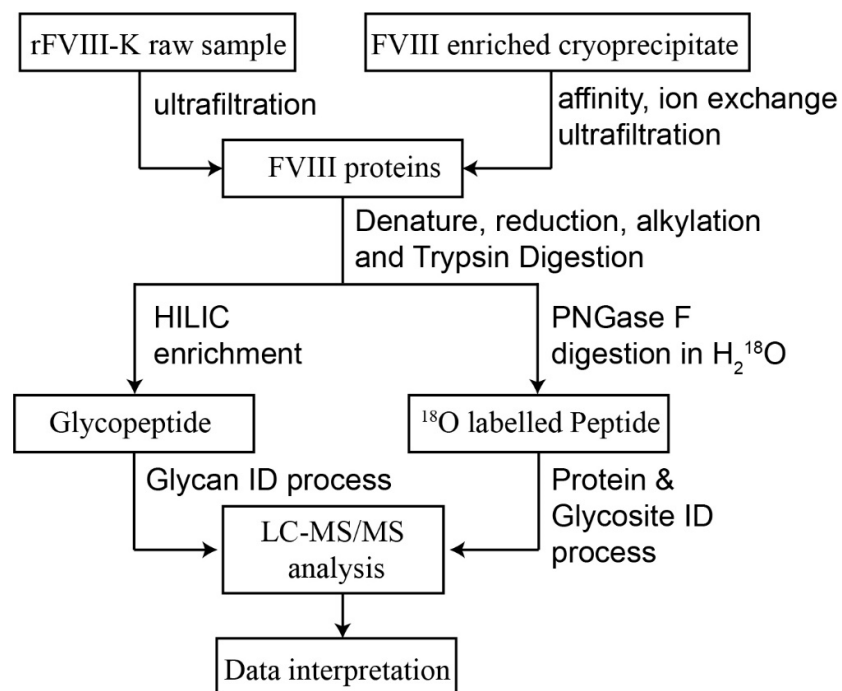

**S1 Fig.** Analytical workflow for glycosylation mapping of pdFVIII-f and rFVIII-K.

Supplement: S1 Fig — (PDF) [file pone.0233576.s001.pdf]
