# Supplementary material for: Comparative glycosylation mapping of plasma-derived and recombinant human factor VIII
Source: PLoS One. 2020 May 22;15(5):e0233576. doi: 10.1371/journal.pone.0233576 (PMC7244179; doi:10.1371/journal.pone.0233576)
Supplement: S2 Fig — (PDF) [file pone.0233576.s002.pdf]

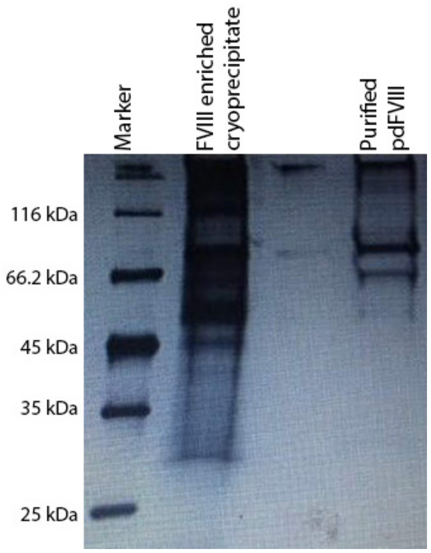

S2 Fig. SDS-PAGE of FVIII enriched cryoprecipitate and purified plasma derived FVIII. The FVIII enriched cryoprecipitate was obtained from Shanghai Lai Shi Blood Products Co., Ltd.
