# Supplementary material for: Comparative glycosylation mapping of plasma-derived and recombinant human factor VIII
Source: PLoS One. 2020 May 22;15(5):e0233576. doi: 10.1371/journal.pone.0233576 (PMC7244179; doi:10.1371/journal.pone.0233576)
Supplement: S1 Table — (PDF) [file pone.0233576.s003.pdf]

S1 Table. Site-specific *N*-glycosylation of pdFVIII-f and rFVIII-K identified by LC-ESI-MS/MS, occupancy of each site and percentage of each glycoform. *Structures of glycoforms are listed in Table 1.

	**pdFVIII-f**			**rFVIII-K**		
**Glycopeptides**	Site Occupancy	*N*-glycoform*	Percentage	Site Occupancy	*N*-glycoform*	Percentage
SFPFN$_{41}$TSVVYK	100%	38	13%	70%	27	100%
		47	8%			
		53	<1%			
		58	78%			
MHTVNGYVN$_{239}$R	85%	10	4%	64%	3	12%
		11	17%		4	33%
		12	16%		5	23%
		17	6%		6	11%
		21	3%		9	2%
		22	35%		10	3%
		38	5%		16	3%
		59	2%		17	2%
		3, 9, 15, 16, 18, 19, 29, 31, 33, 51, 52, 56, 60	<2%		1, 2, 7, 8, 11-15, 18-23, 27, 30-32, 33, 34, 37,39, 44	<2%
QFN$_{757}$ATTIPENDIEK	50%	9	70%	N.D.		
		38	30%			
IQN$_{784}$VSSSDLLMLLR	10%	41	100%	N.D.		
MLMDKN$_{1055}$ATALR	100%	22	45%	100%	34	10%
		30	2%		35	2%
		38	38%		36	8%
		49	6%		37	58%
		55	3%		43	2%
		57	4%		44	14%
		33, 40	<2%		50	3%
					25, 26, 41, 42	<2%
LNHMSN$_{1066}$K	N.D.			100%	22	50%
					47	6%
					48	40%
					49	4%
NLFLTNLDNLHEN$_{1185}$NTHNQEK	60%	23	15%	75%	37	6%
		49	81%		44	2%
		54	4%		48	89%
					24, 38	<2%
KGEEENLEGLGN$_{1282}$QTK	55%	37	18%	89%	28	37%
		38	82%		29	11%
					30	24%
					33	4%
					40	11%
					47	2.0%
SHSIPQAN$_{1384}$R	N.D.			12%	22	9%
					37	4%
					48	87%
VLFQDN$_{1412}$SSHLPAASYR	66%	17	25%	60%	30	57%
		22	75%		33	7%
					37	36%
EDFDIYDEDEN$_{1685}$QSPR	12%	23	100%	N.D.		
KNFVKPN$_{1810}$ETK	60%	17	3%	53%	23	2%
		38	49%		36	7%
		49	35%		37	38%
		61	12%		43	13%
					45	9%
					47	3%
					48	28%
					28, 46	<2%
GN$_{2118}$STGTLMVFFGNVDSSGIK	20%	5	100%	N.D.		
