# Supplementary material for: Comparative glycosylation mapping of plasma-derived and recombinant human factor VIII
Source: PLoS One. 2020 May 22;15(5):e0233576. doi: 10.1371/journal.pone.0233576 (PMC7244179; doi:10.1371/journal.pone.0233576)
Supplement: S1 File — (PDF) [file pone.0233576.s004.pdf]

S1 File. Spectra of identified glycoforms

1-N

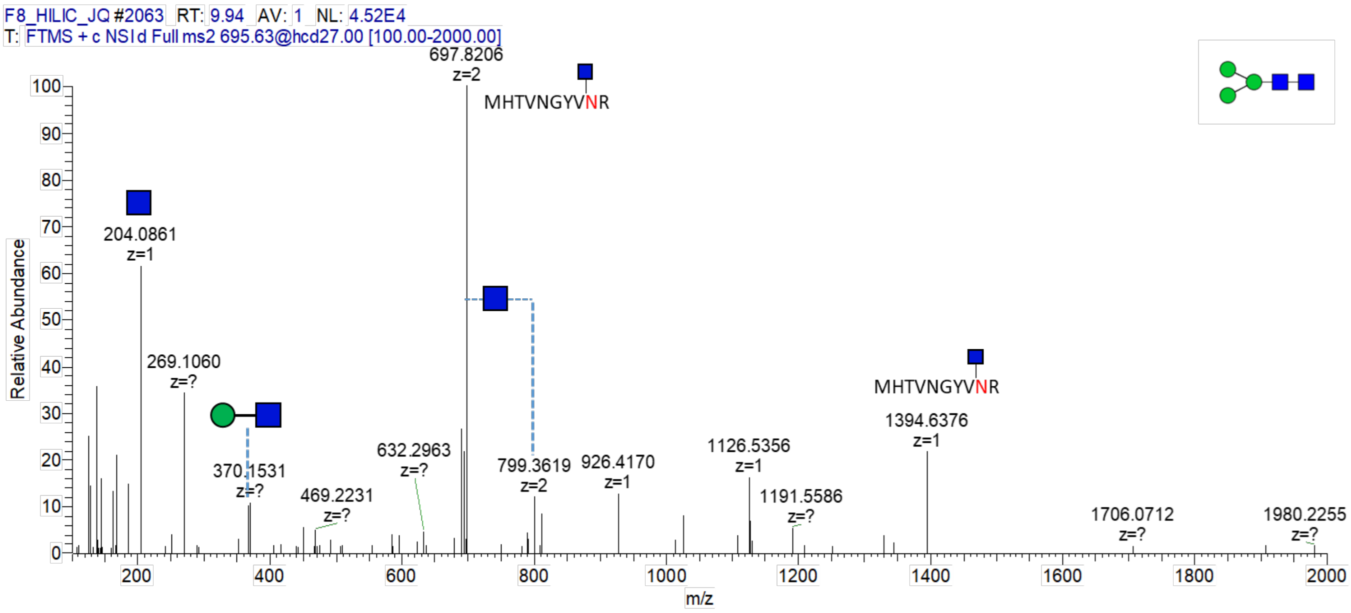

2-N

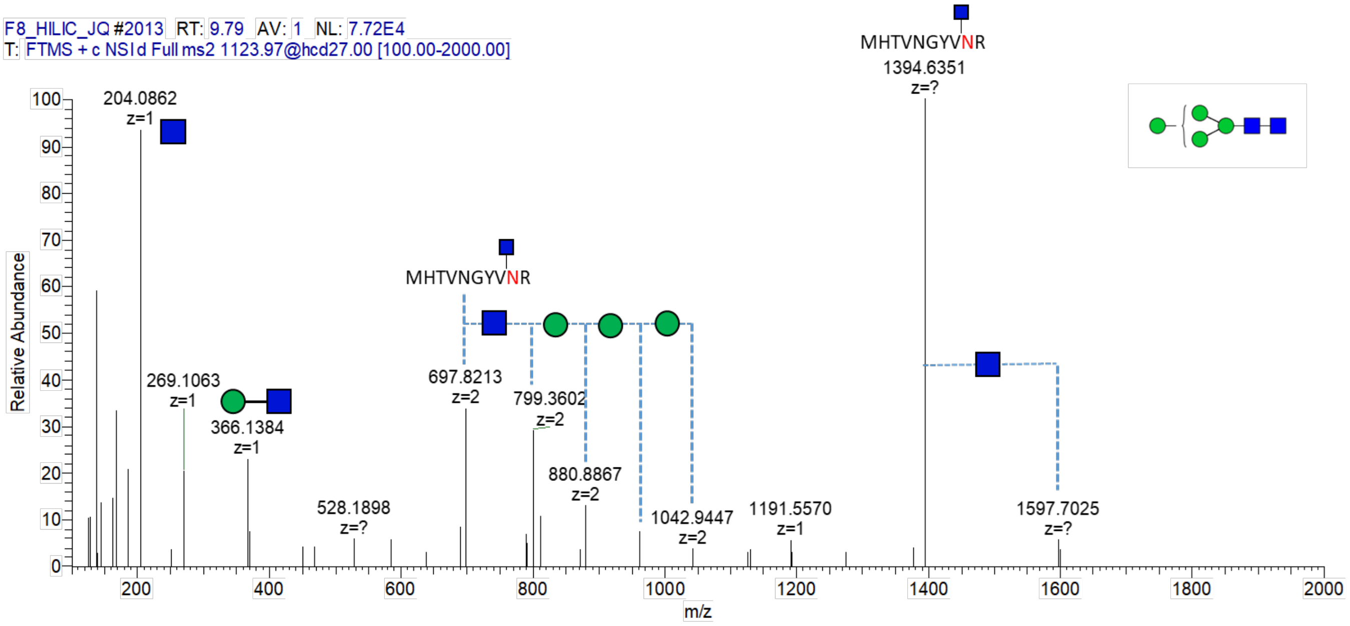

3-N

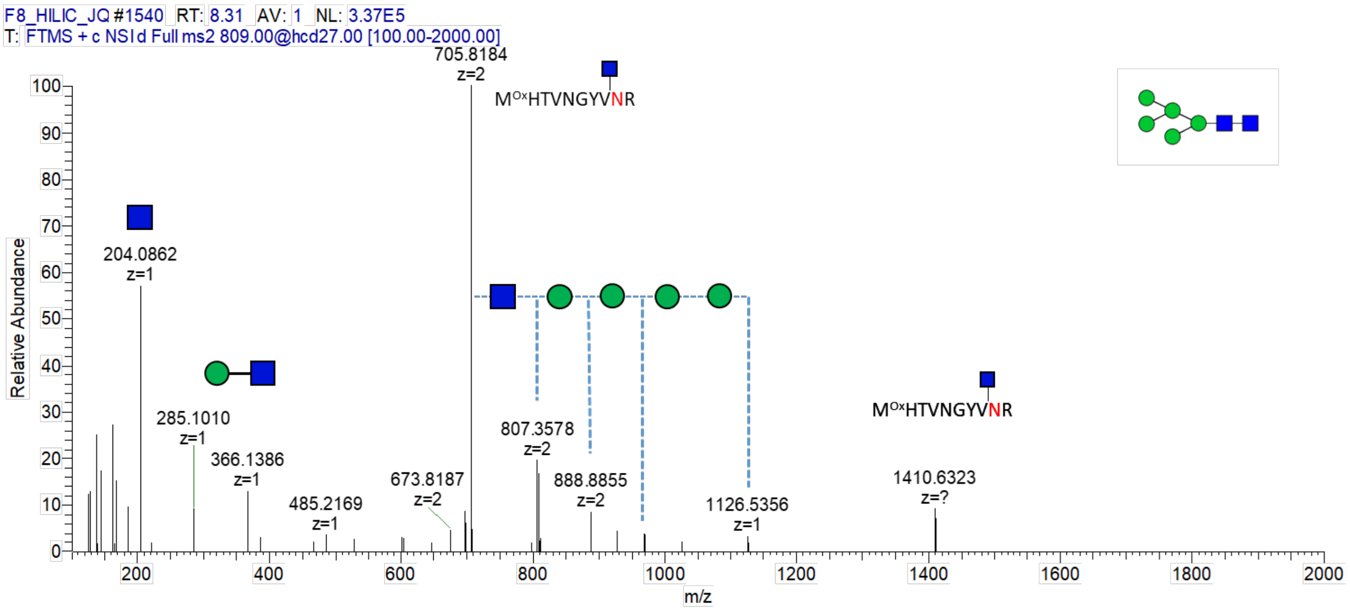

F8_HILIC_JQ #1540 RT: 8.31 AV: 1 NL: 3.37E5
T: FTMS + c NSI d Full ms2 809.00@hcd27.00 [100.00-2000.00]
705.8184
z=2
M^Ox HTVNGYVNR
204.0862
z=1
285.1010
z=1
366.1386
z=1
485.2169
z=1
673.8187
z=2
807.3578
z=2
888.8855
z=2
1126.5356
z=1
M^Ox HTVNGYVNR
1410.6323
z=?
Relative Abundance
m/z

4-N

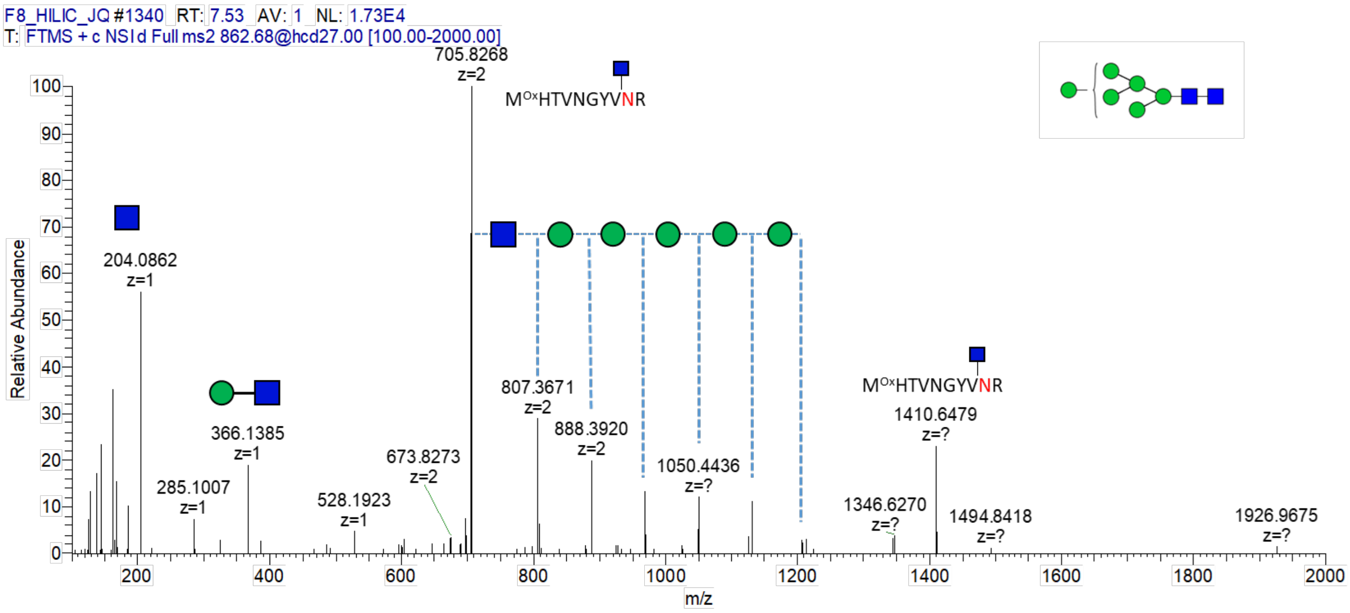

F8_HILIC_JQ #1340 RT: 7.53 AV: 1 NL: 1.73E4
T: FTMS + c NSI d Full ms2 862.68@hcd27.00 [100.00-2000.00]
705.8268
z=2
M^Ox HTVNGYVNR
204.0862
z=1
366.1385
z=1
285.1007
z=1
528.1923
z=1
673.8273
z=2
807.3671
z=2
888.3920
z=2
1050.4436
z=?
M^Ox HTVNGYVNR
1410.6479
z=?
1346.6270
z=?
1494.8418
z=?
1926.9675
z=?
Relative Abundance
m/z

5-N

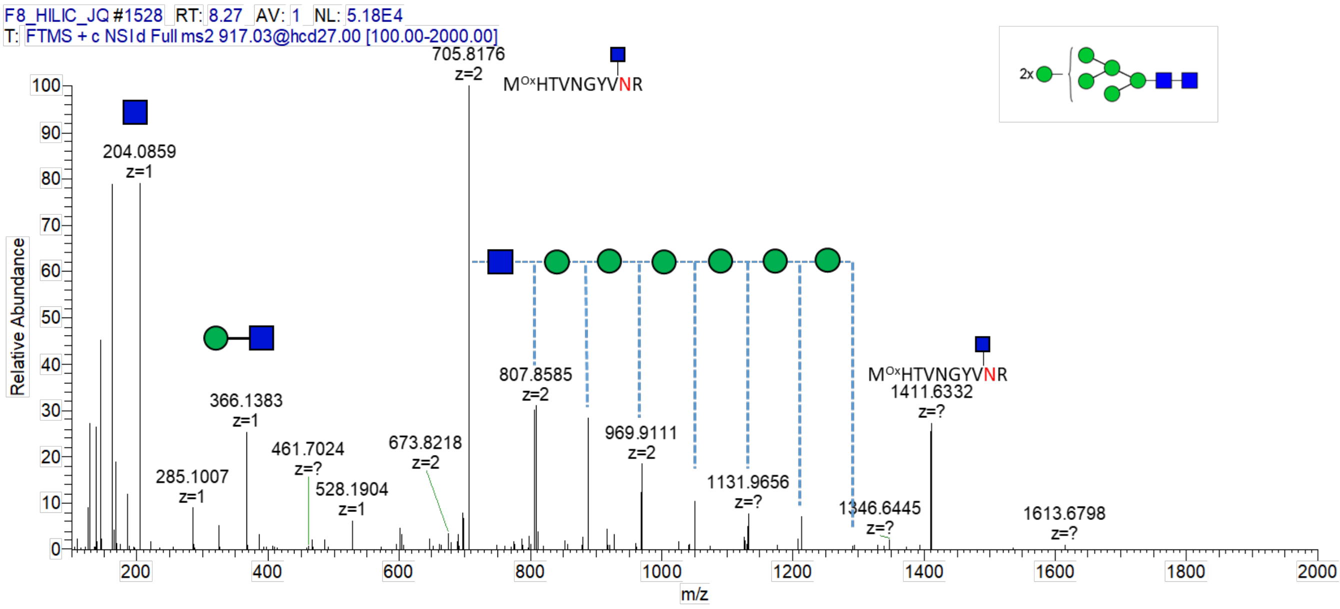
F8_HILIC_JQ #1528 RT: 8.27 AV: 1 NL: 5.18E4
T: FTMS + c NSI d Full ms2 917.03@hcd27.00 [100.00-2000.00]
705.8176
z=2
M^OxHTVNGYVNR
2x
204.0859
z=1
366.1383
z=1
285.1007
z=1
461.7024
z=?
528.1904
z=1
673.8218
z=2
807.8585
z=2
969.9111
z=2
1131.9656
z=?
1346.6445
z=?
M^OxHTVNGYVNR
1411.6332
z=?
1613.6798
z=?
Relative Abundance
m/z

6-N

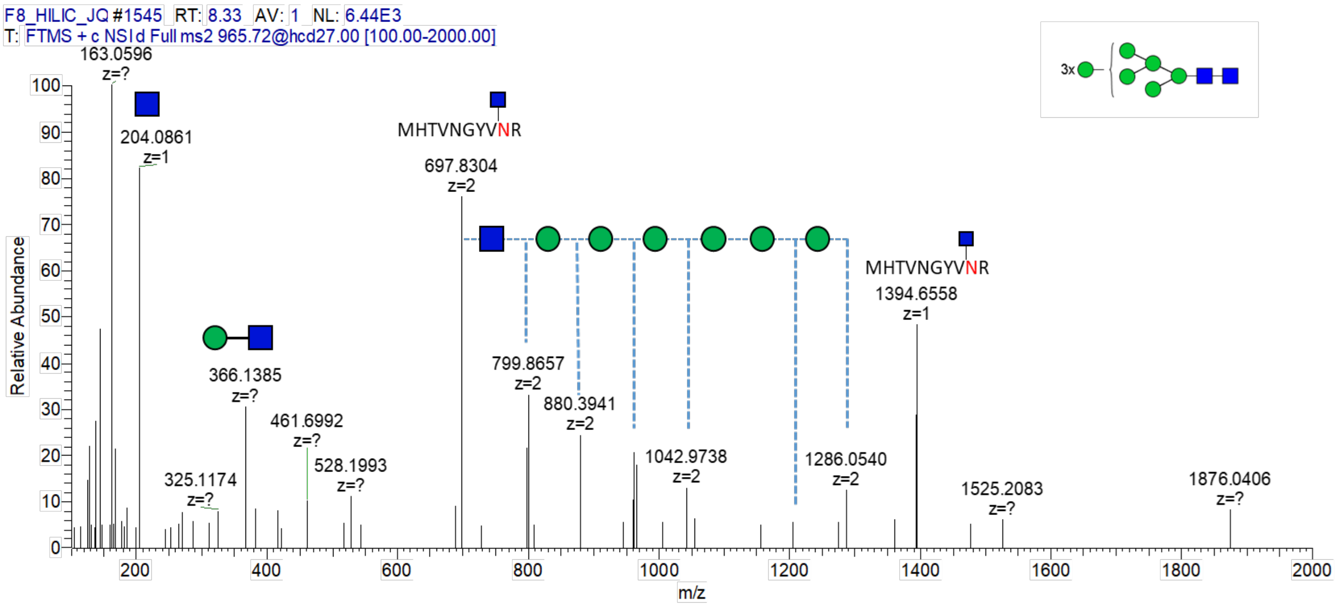
F8_HILIC_JQ #1545 RT: 8.33 AV: 1 NL: 6.44E3
T: FTMS + c NSI d Full ms2 965.72@hcd27.00 [100.00-2000.00]
163.0596
z=?
3x
204.0861
z=1
MHTVNGYVNR
697.8304
z=2
366.1385
z=?
325.1174
z=?
461.6992
z=?
528.1993
z=?
799.8657
z=2
880.3941
z=2
1042.9738
z=2
1286.0540
z=2
MHTVNGYVNR
1394.6558
z=1
1525.2083
z=?
1876.0406
z=?
Relative Abundance
m/z

7-N

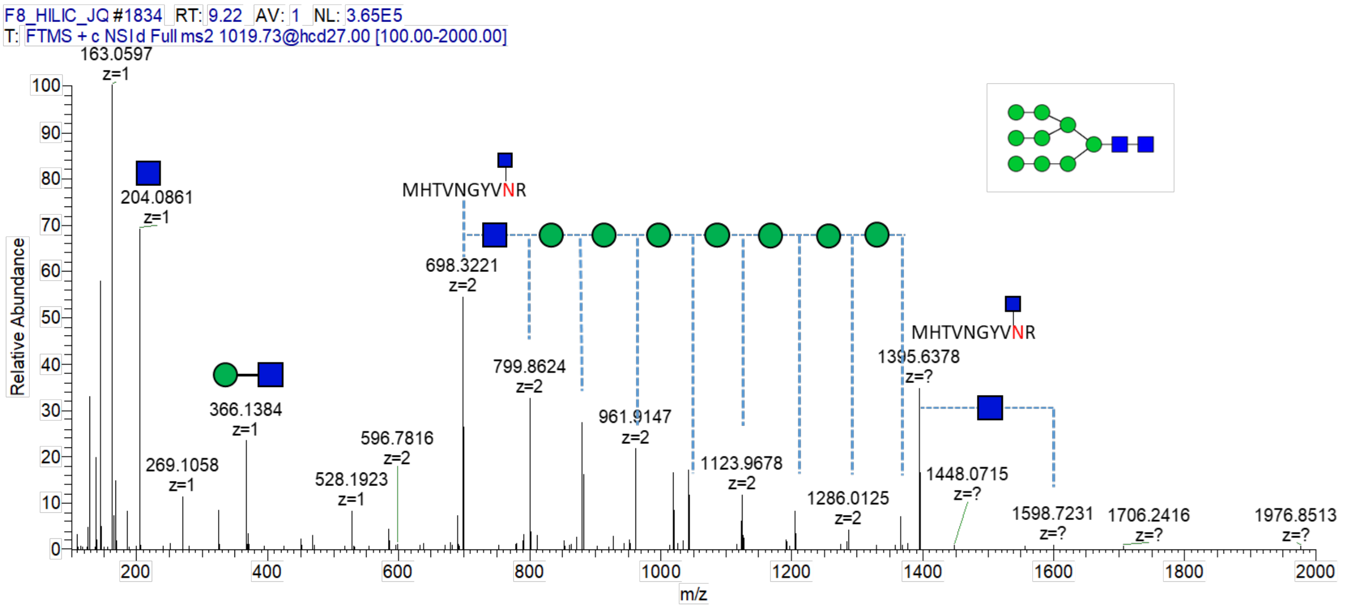

F8_HILIC_JQ #1834 RT: 9.22 AV: 1 NL: 3.65E5
T: FTMS + c NSI d Full ms2 1019.73@hcd27.00 [100.00-2000.00]
163.0597 z=1
204.0861 z=1
269.1058 z=1
366.1384 z=1
528.1923 z=1
596.7816 z=2
698.3221 z=2
MHTVNGYVNR
799.8624 z=2
961.9147 z=2
1123.9678 z=2
1286.0125 z=2
1395.6378 z=?
1448.0715 z=?
1598.7231 z=?
1706.2416 z=?
1976.8513 z=?
Relative Abundance
m/z

8-N

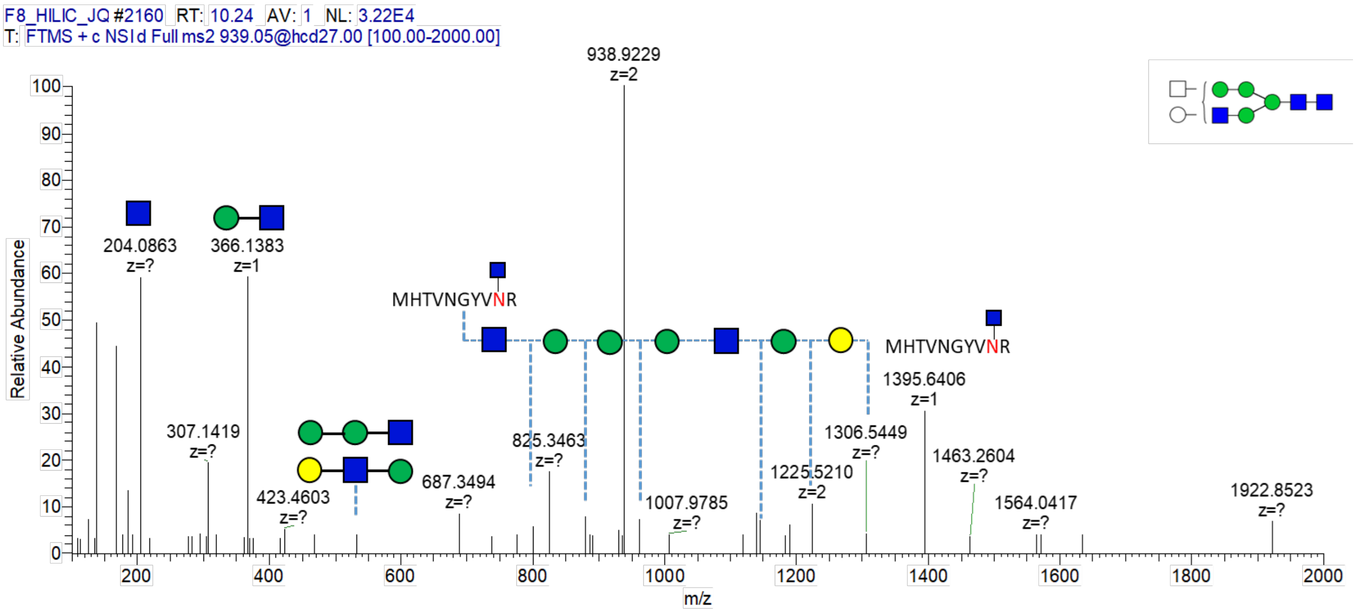

F8_HILIC_JQ #2160 RT: 10.24 AV: 1 NL: 3.22E4
T: FTMS + c NSI d Full ms2 939.05@hcd27.00 [100.00-2000.00]
938.9229 z=2
204.0863 z=?
366.1383 z=1
307.1419 z=?
423.4603 z=?
687.3494 z=?
825.3463 z=?
1007.9785 z=?
1225.5210 z=2
1306.5449 z=?
1395.6406 z=1
1463.2604 z=?
1564.0417 z=?
1922.8523 z=?
MHTVNGYVNR
Relative Abundance
m/z

9-N

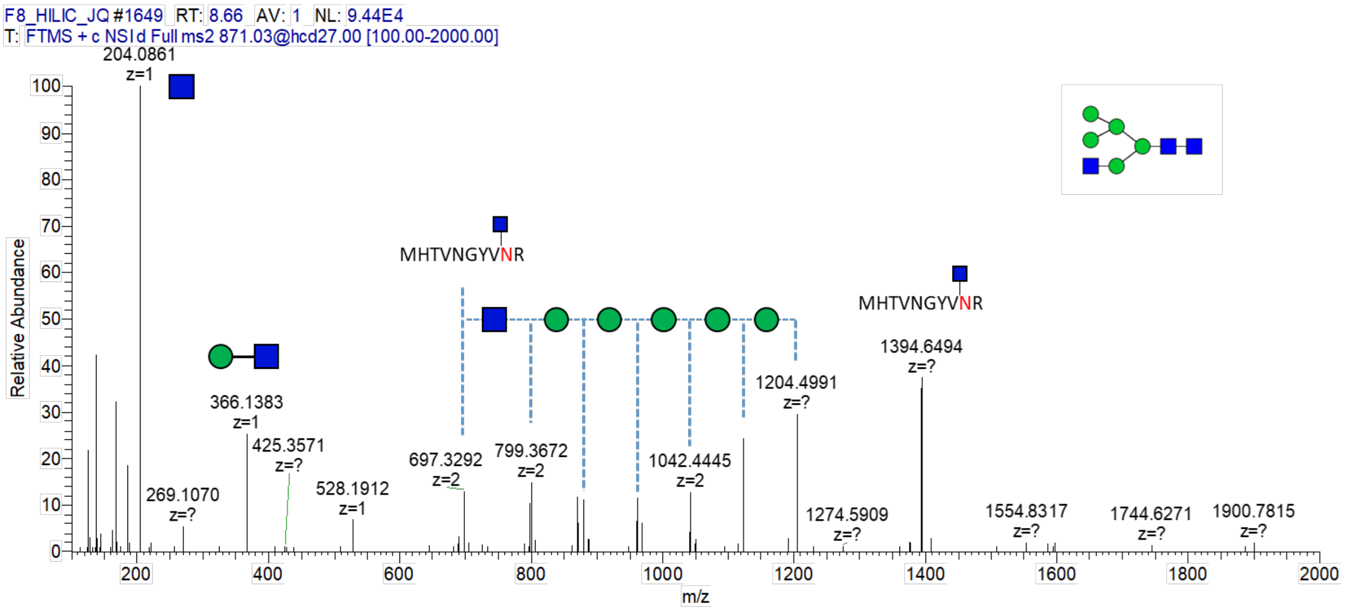
F8_HILIC_JQ #1649 RT: 8.66 AV: 1 NL: 9.44E4
T: FTMS + c NSI d Full ms2 871.03@hcd27.00 [100.00-2000.00]
Relative Abundance
m/z
204.0861 z=1
366.1383 z=1
425.3571 z=?
269.1070 z=?
528.1912 z=1
697.3292 z=2
799.3672 z=2
1042.4445 z=2
1204.4991 z=?
1274.5909 z=?
1394.6494 z=?
1554.8317 z=?
1744.6271 z=?
1900.7815 z=?
MHTVNGYVNR
MHTVNGYVNR

10-N

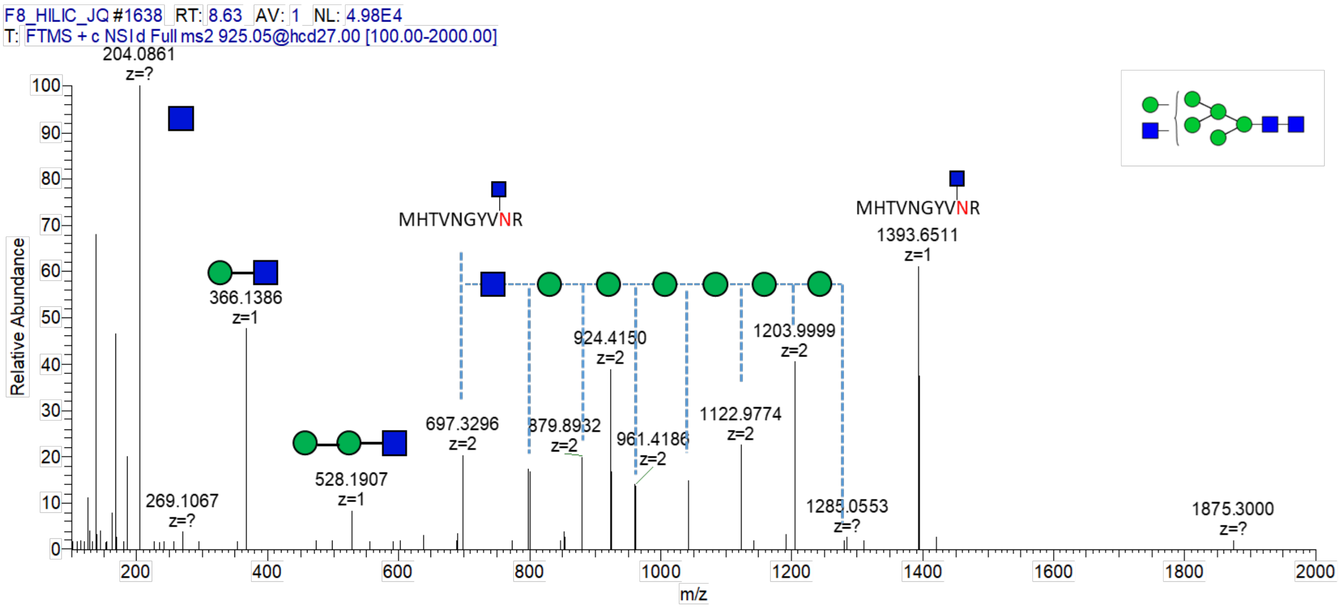
F8_HILIC_JQ #1638 RT: 8.63 AV: 1 NL: 4.98E4
T: FTMS + c NSI d Full ms2 925.05@hcd27.00 [100.00-2000.00]
Relative Abundance
m/z
204.0861 z=?
366.1386 z=1
269.1067 z=?
528.1907 z=1
697.3296 z=2
879.8932 z=2
924.4150 z=2
961.4186 z=2
1122.9774 z=2
1203.9999 z=2
1285.0553 z=?
1393.6511 z=1
1875.3000 z=?
MHTVNGYVNR
MHTVNGYVNR

11-N

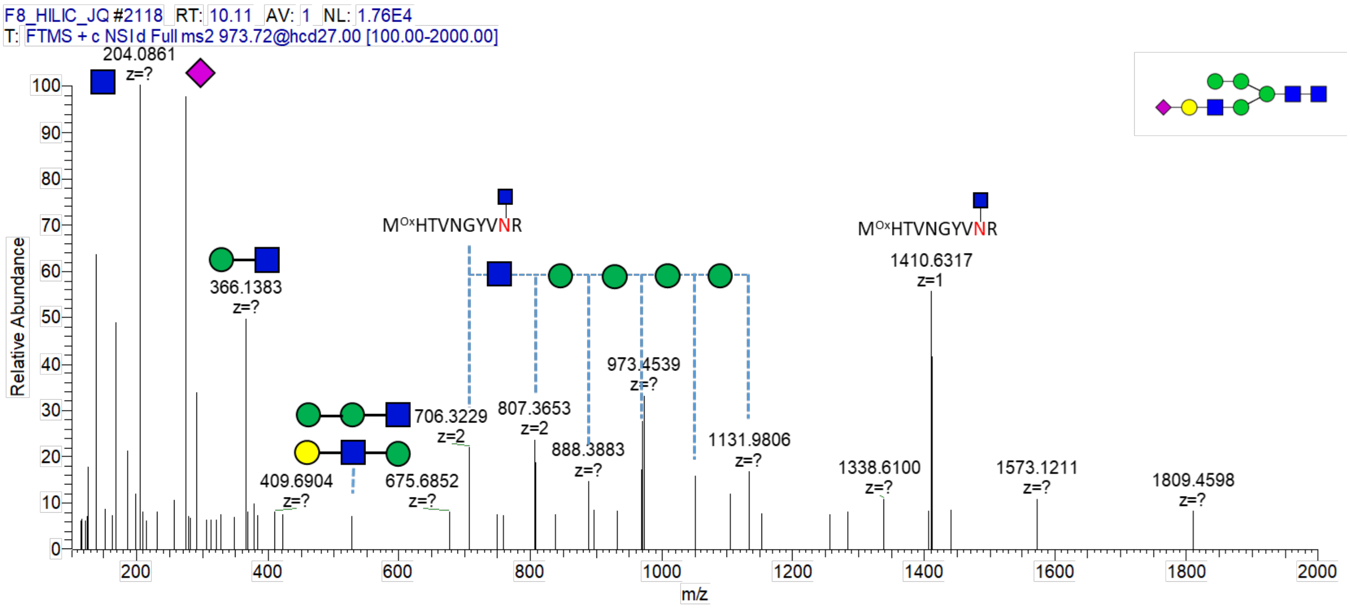

F8_HILIC_JQ #2118 RT: 10.11 AV: 1 NL: 1.76E4
T: FTMS + c NSI d Full ms2 973.72@hcd27.00 [100.00-2000.00]
204.0861
z=?
366.1383
z=?
409.6904
z=?
675.6852
z=?
706.3229
z=2
807.3653
z=2
888.3883
z=?
973.4539
z=?
1131.9806
z=?
1338.6100
z=?
MOxHTVNGYVNR
1410.6317
z=1
1573.1211
z=?
1809.4598
z=?
Relative Abundance
m/z

12-N

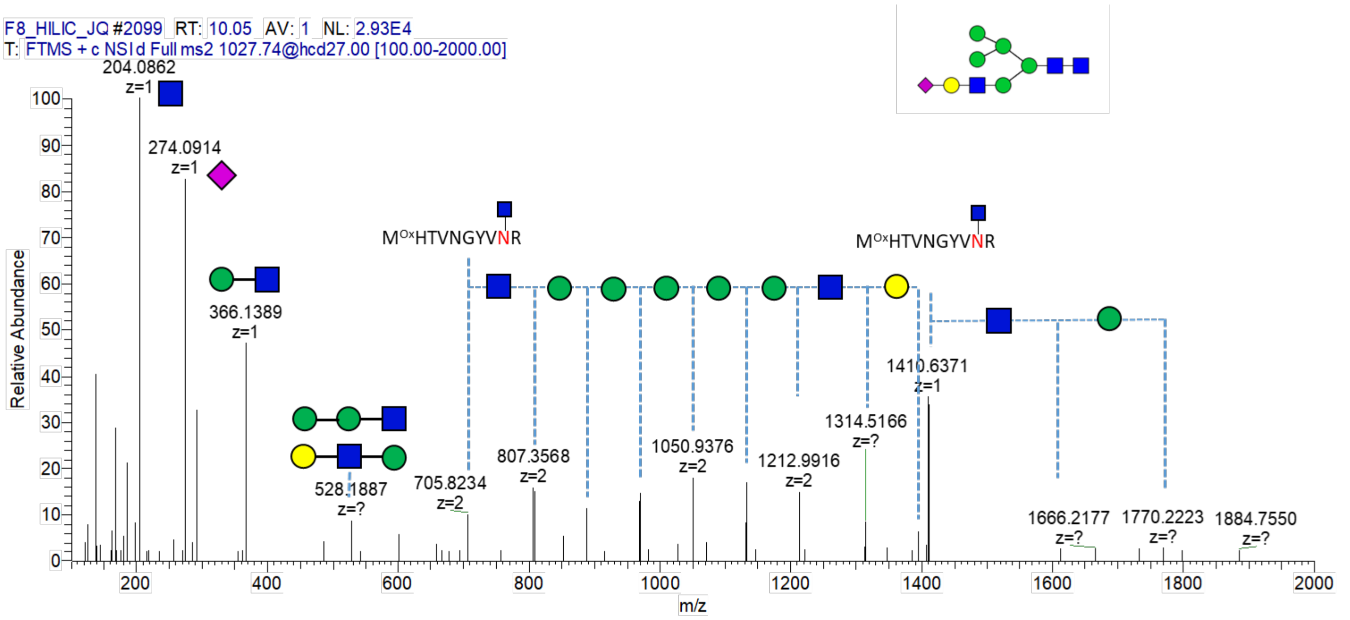

F8_HILIC_JQ #2099 RT: 10.05 AV: 1 NL: 2.93E4
T: FTMS + c NSI d Full ms2 1027.74@hcd27.00 [100.00-2000.00]
204.0862
z=1
274.0914
z=1
366.1389
z=1
528.1887
z=?
705.8234
z=2
807.3568
z=2
1050.9376
z=2
1212.9916
z=2
1314.5166
z=?
MOxHTVNGYVNR
1410.6371
z=1
1666.2177
z=?
1770.2223
z=?
1884.7550
z=?
Relative Abundance
m/z

13-N

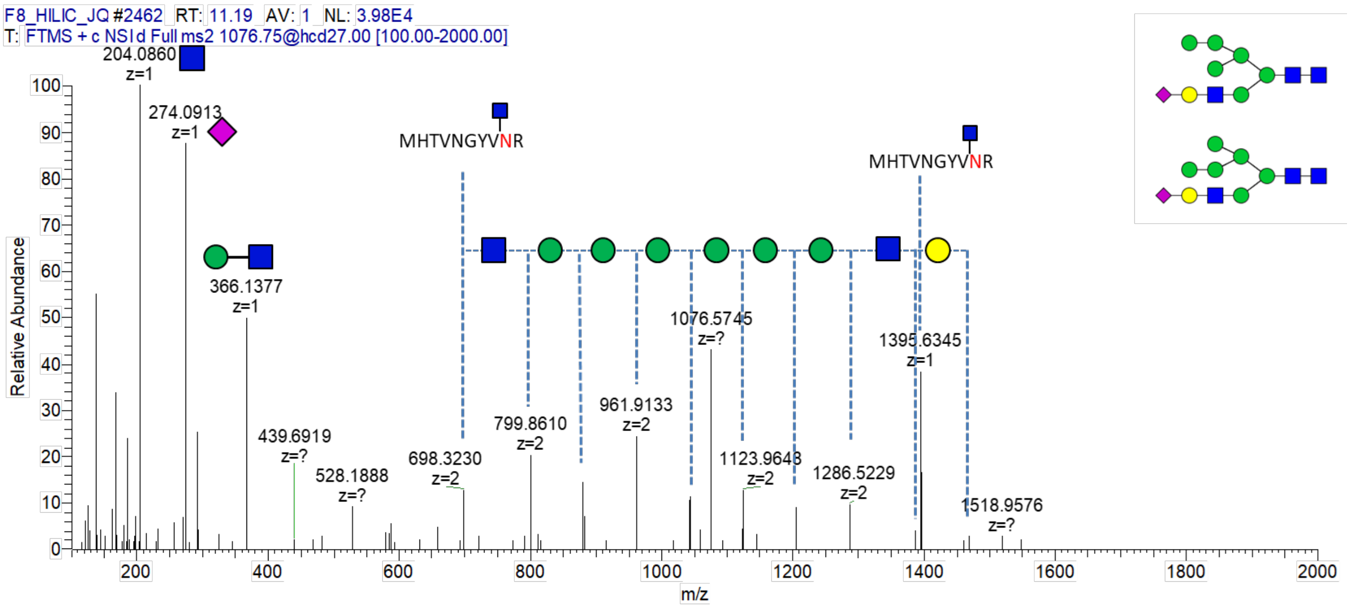

F8_HILIC_JQ #2462 RT: 11.19 AV: 1 NL: 3.98E4
T: FTMS + c NSI d Full ms2 1076.75@hcd27.00 [100.00-2000.00]
204.0860
z=1
274.0913
z=1
366.1377
z=1
439.6919
z=?
528.1888
z=?
698.3230
z=2
799.8610
z=2
961.9133
z=2
1076.5745
z=?
1123.9648
z=2
1286.5229
z=2
1395.6345
z=1
1518.9576
z=?
MHTVNGYVNR
MHTVNGYVNR
Relative Abundance
m/z

14-N

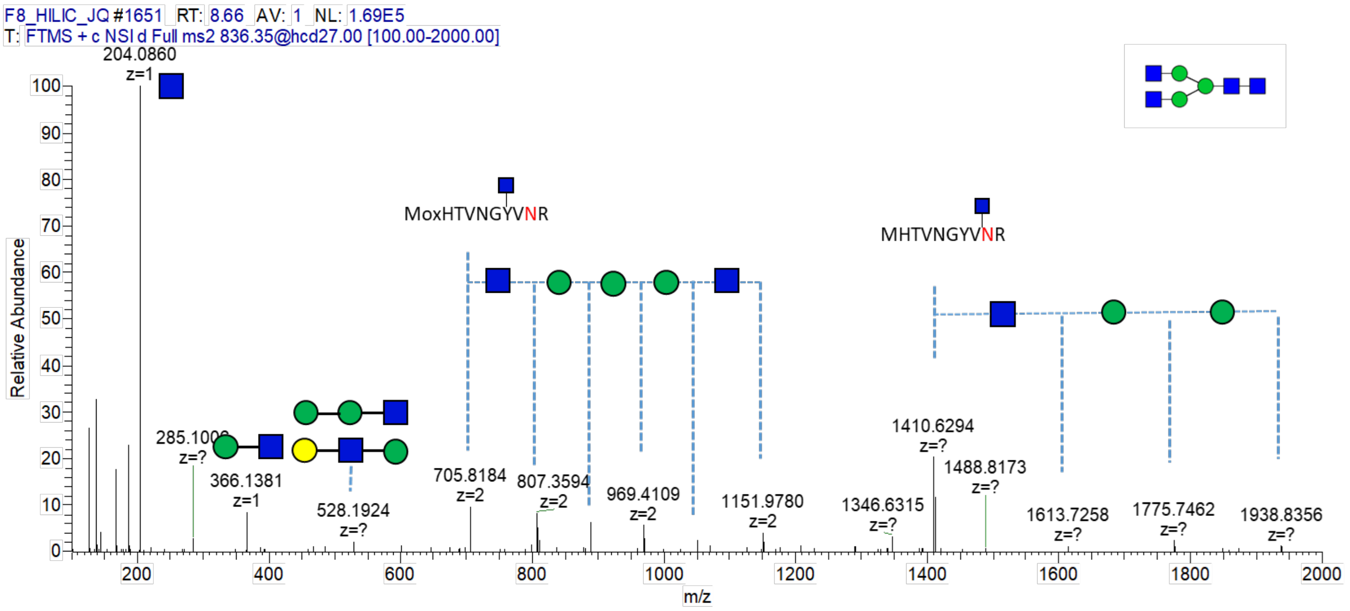

F8_HILIC_JQ #1651 RT: 8.66 AV: 1 NL: 1.69E5
T: FTMS + c NSI d Full ms2 836.35@hcd27.00 [100.00-2000.00]
204.0860
z=1
285.1009
z=?
366.1381
z=1
528.1924
z=?
705.8184
z=2
807.3594
z=2
969.4109
z=2
1151.9780
z=2
1346.6315
z=?
1410.6294
z=?
1488.8173
z=?
1613.7258
z=?
1775.7462
z=?
1938.8356
z=?
MoxHTVNGYVNR
MHTVNGYVNR
Relative Abundance
m/z

15-N

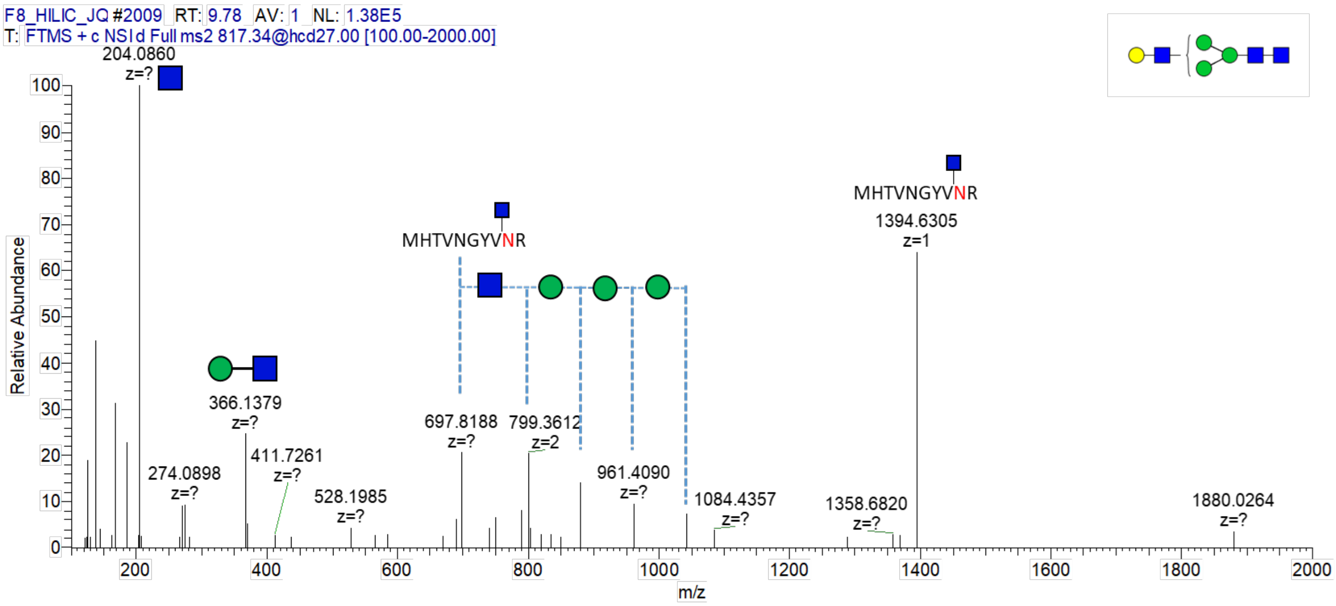

F8_HILIC_JQ #2009 RT: 9.78 AV: 1 NL: 1.38E5
T: FTMS + c NSI d Full ms2 817.34@hcd27.00 [100.00-2000.00]
204.0860 z=?
366.1379 z=?
274.0898 z=?
411.7261 z=?
528.1985 z=?
MHTVNGYVNR
697.8188 z=?
799.3612 z=2
961.4090 z=?
1084.4357 z=?
1358.6820 z=?
MHTVNGYVNR
1394.6305 z=1
1880.0264 z=?
Relative Abundance
m/z

16-N

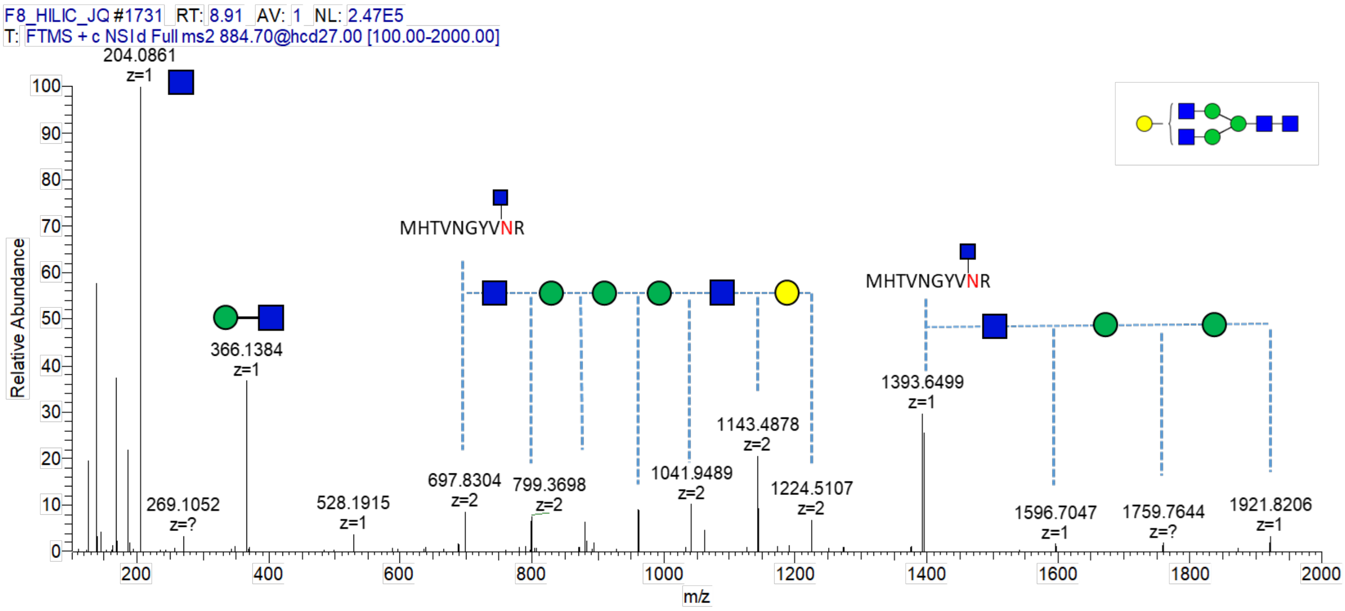

F8_HILIC_JQ #1731 RT: 8.91 AV: 1 NL: 2.47E5
T: FTMS + c NSI d Full ms2 884.70@hcd27.00 [100.00-2000.00]
204.0861 z=1
366.1384 z=1
269.1052 z=?
528.1915 z=1
MHTVNGYVNR
697.8304 z=2
799.3698 z=2
1041.9489 z=2
1143.4878 z=2
1224.5107 z=2
MHTVNGYVNR
1393.6499 z=1
1596.7047 z=1
1759.7644 z=?
1921.8206 z=1
Relative Abundance
m/z

17-N

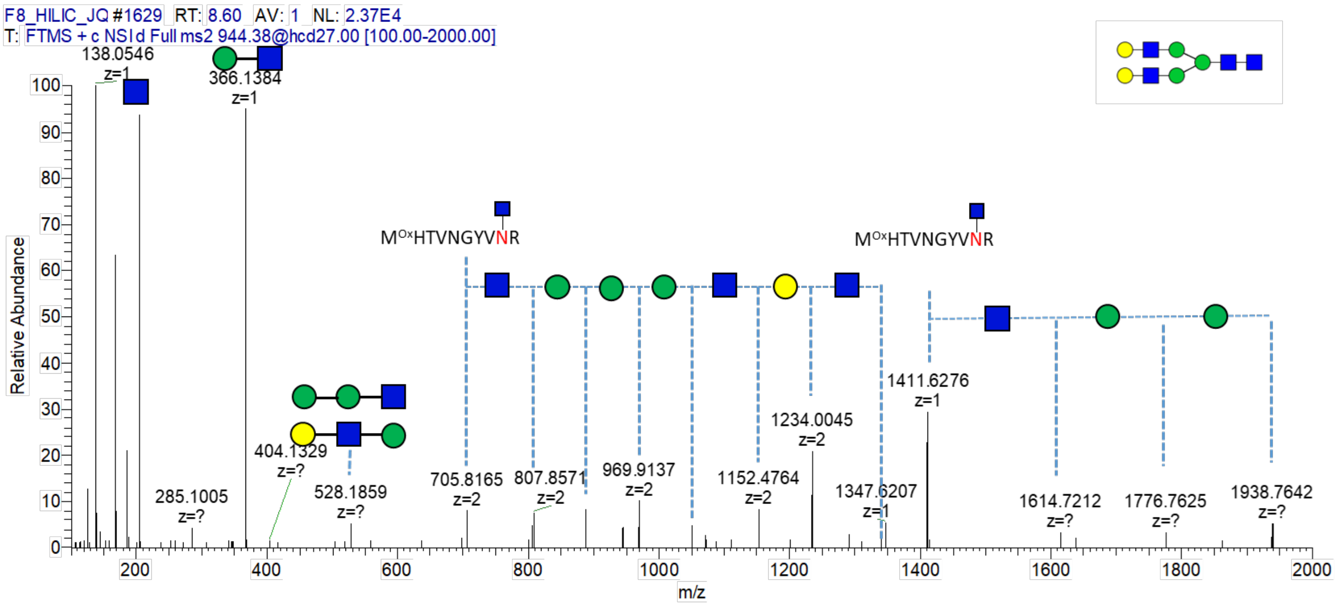

F8_HILIC_JQ #1629 RT: 8.60 AV: 1 NL: 2.37E4
T: FTMS + c NSI d Full ms2 944.38@hcd27.00 [100.00-2000.00]
138.0546 z=1
366.1384 z=1
404.1329 z=?
285.1005 z=?
528.1859 z=?
705.8165 z=2
807.8571 z=2
969.9137 z=2
1152.4764 z=2
1234.0045 z=2
1347.6207 z=1
1411.6276 z=1
1614.7212 z=?
1776.7625 z=?
1938.7642 z=?
MOxHTVNGYVNR
Relative Abundance
m/z

18-N

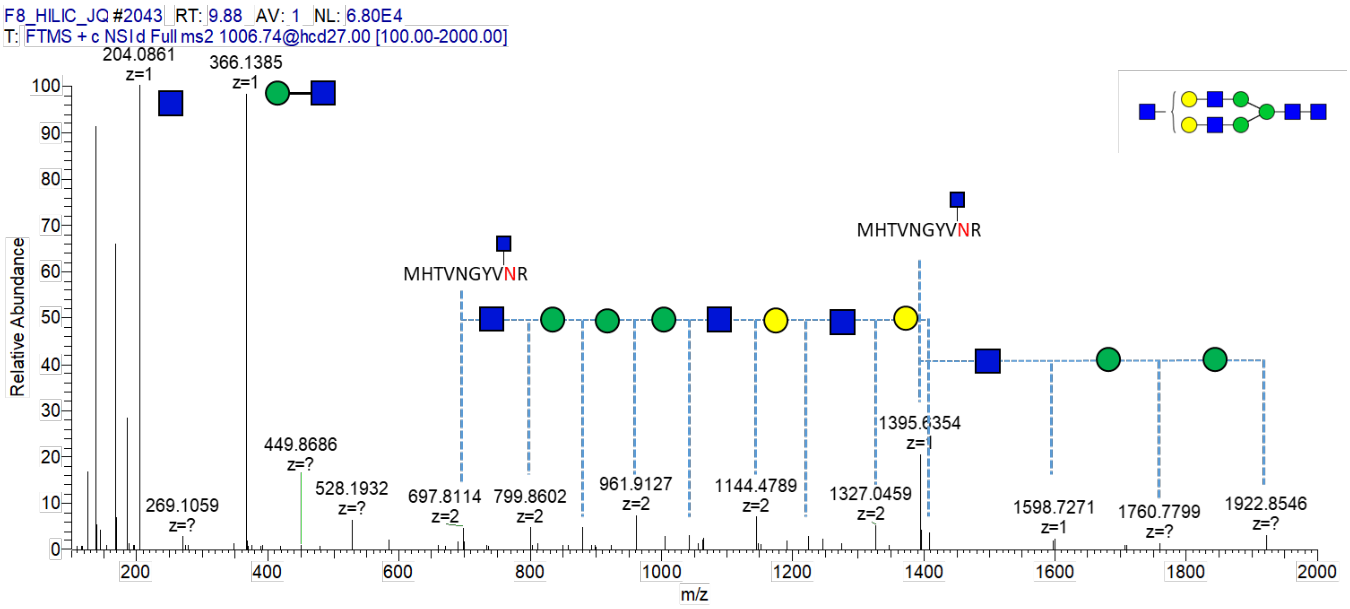

F8_HILIC_JQ #2043 RT: 9.88 AV: 1 NL: 6.80E4
T: FTMS + c NSI d Full ms2 1006.74@hcd27.00 [100.00-2000.00]
204.0861 z=1
366.1385 z=1
269.1059 z=?
449.8686 z=?
528.1932 z=?
697.8114 z=2
799.8602 z=2
961.9127 z=2
1144.4789 z=2
1327.0459 z=2
1395.6354 z=1
1598.7271 z=1
1760.7799 z=?
1922.8546 z=?
MHTVNGYVNR
Relative Abundance
m/z

19-N

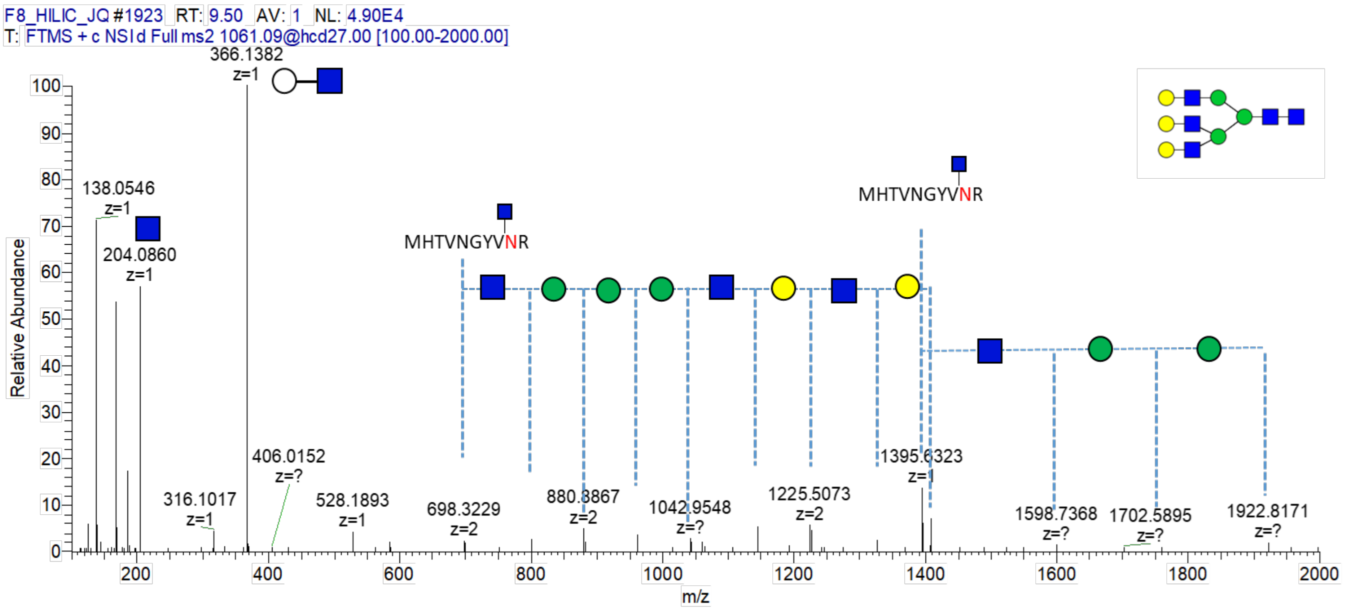
F8_HILIC_JQ #1923 RT: 9.50 AV: 1 NL: 4.90E4
T: FTMS + c NSI d Full ms2 1061.09@hcd27.00 [100.00-2000.00]
366.1382
z=1
138.0546
z=1
204.0860
z=1
316.1017
z=1
406.0152
z=?
528.1893
z=1
698.3229
z=2
880.8867
z=2
1042.9548
z=?
1225.5073
z=2
1395.6323
z=1
1598.7368
z=?
1702.5895
z=?
1922.8171
z=?
MHTVNGYVNR
MHTVNGYVNR
Relative Abundance
m/z

20-N

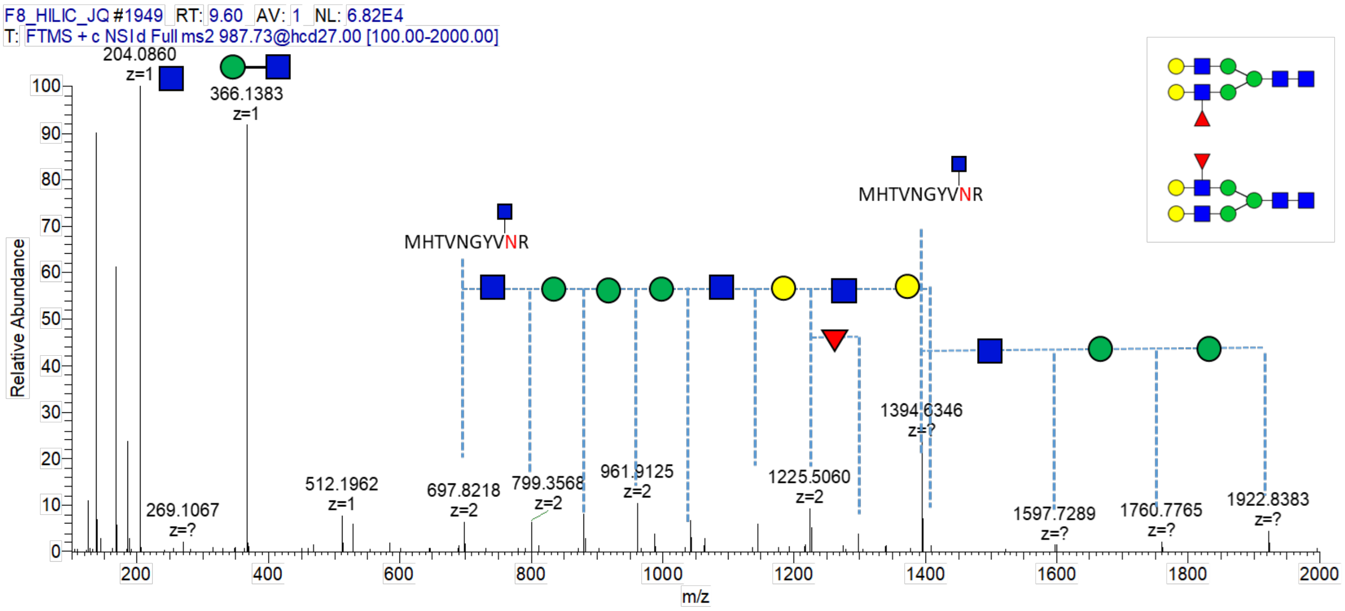
F8_HILIC_JQ #1949 RT: 9.60 AV: 1 NL: 6.82E4
T: FTMS + c NSI d Full ms2 987.73@hcd27.00 [100.00-2000.00]
204.0860
z=1
366.1383
z=1
269.1067
z=?
512.1962
z=1
697.8218
z=2
799.3568
z=2
961.9125
z=2
1225.5060
z=2
1394.6346
z=?
1597.7289
z=?
1760.7765
z=?
1922.8383
z=?
MHTVNGYVNR
MHTVNGYVNR
Relative Abundance
m/z

21-N

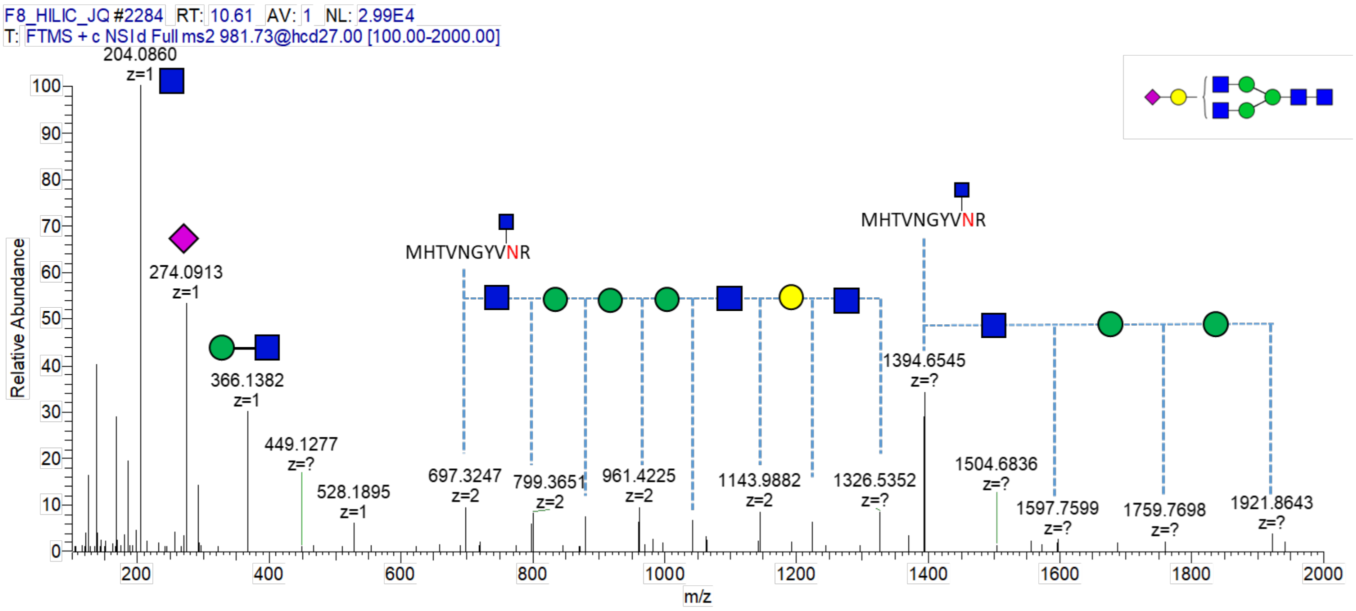

F8_HILIC_JQ #2284 RT: 10.61 AV: 1 NL: 2.99E4
T: FTMS + c NSI d Full ms2 981.73@hcd27.00 [100.00-2000.00]
Relative Abundance
m/z
MHTVNGYVNR
MHTVNGYVNR
204.0860 z=1
274.0913 z=1
366.1382 z=1
449.1277 z=?
528.1895 z=1
697.3247 z=2
799.3651 z=2
961.4225 z=2
1143.9882 z=2
1326.5352 z=?
1394.6545 z=?
1504.6836 z=?
1597.7599 z=?
1759.7698 z=?
1921.8643 z=?

22-N

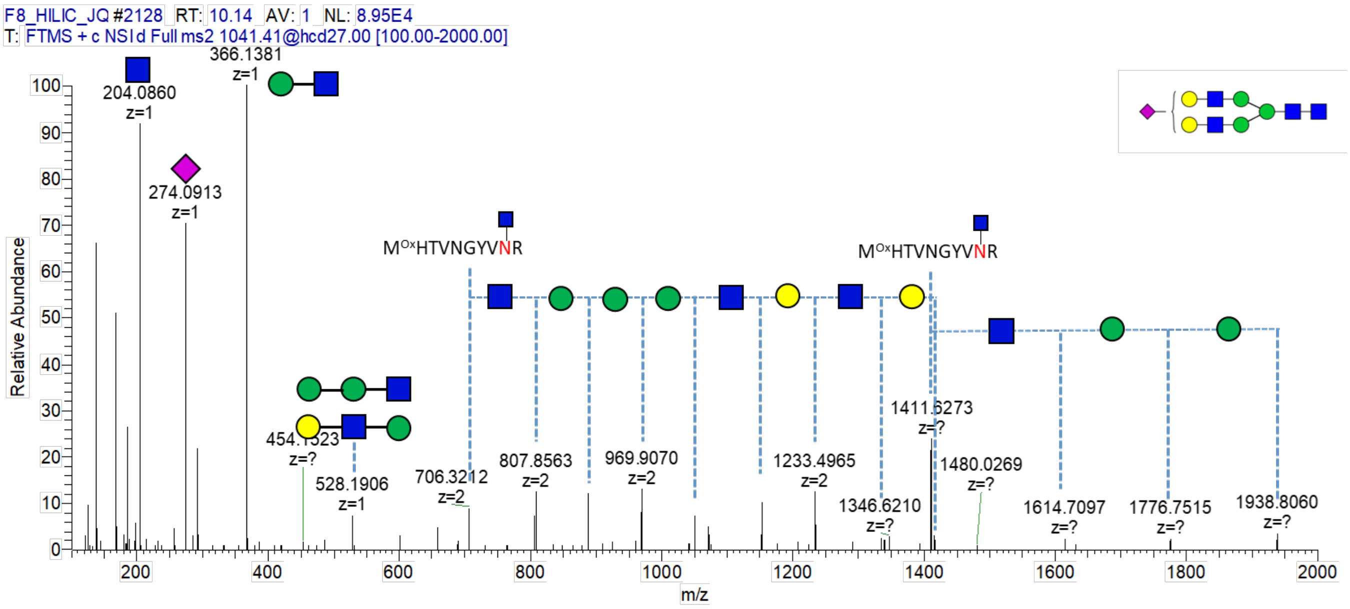

F8_HILIC_JQ #2128 RT: 10.14 AV: 1 NL: 8.95E4
T: FTMS + c NSI d Full ms2 1041.41@hcd27.00 [100.00-2000.00]
Relative Abundance
m/z
M^OxHTVNGYVNR
M^OxHTVNGYVNR
204.0860 z=1
274.0913 z=1
366.1381 z=1
454.1523 z=?
528.1906 z=1
706.3212 z=2
807.8563 z=2
969.9070 z=2
1233.4965 z=2
1346.6210 z=?
1411.6273 z=?
1480.0269 z=?
1614.7097 z=?
1776.7515 z=?
1938.8060 z=?

23-N

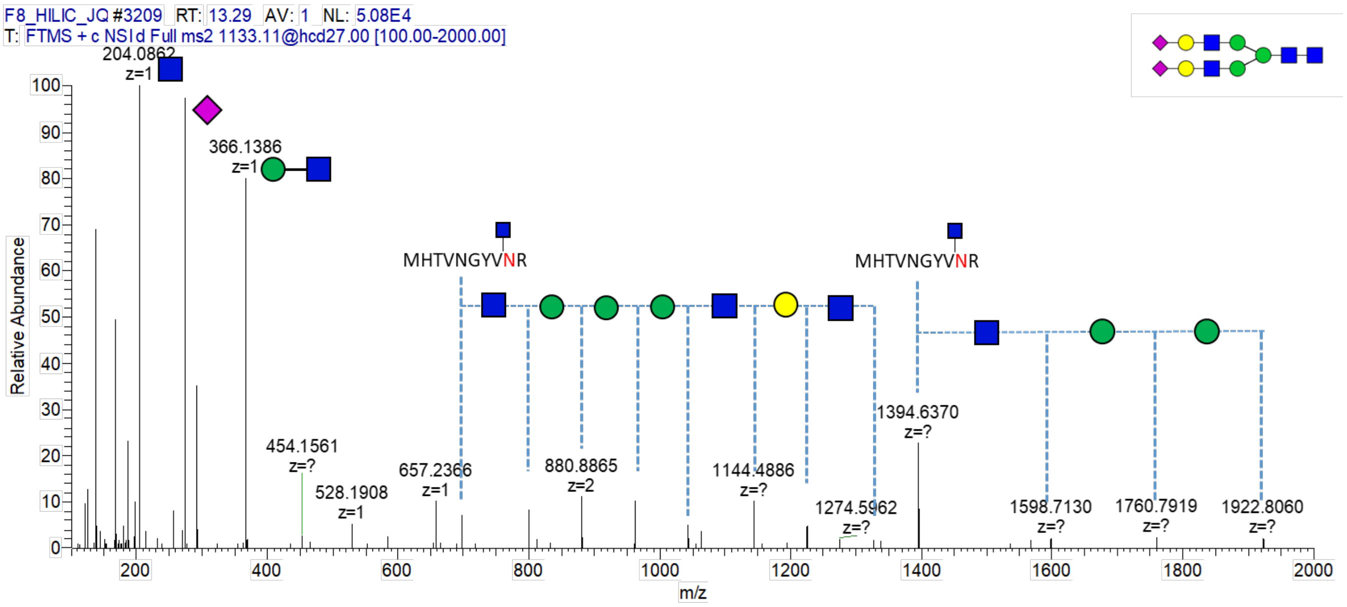
F8_HILIC_JQ #3209 RT: 13.29 AV: 1 NL: 5.08E4
T: FTMS + c NSI d Full ms2 1133.11@hcd27.00 [100.00-2000.00]
Relative Abundance
m/z
204.0862 z=1
366.1386 z=1
454.1561 z=?
528.1908 z=1
657.2366 z=1
880.8865 z=2
1144.4886 z=?
1274.5962 z=?
1394.6370 z=?
1598.7130 z=?
1760.7919 z=?
1922.8060 z=?
MHTVNGYVNR
MHTVNGYVNR

24-N

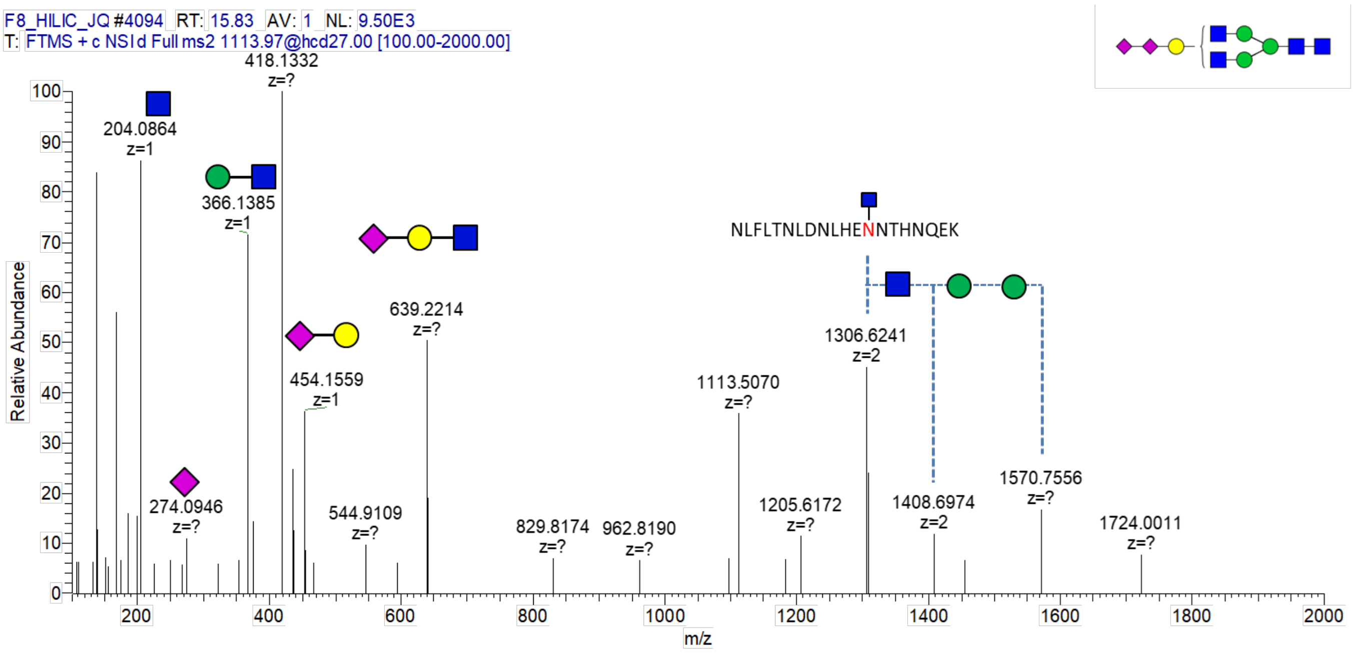
F8_HILIC_JQ #4094 RT: 15.83 AV: 1 NL: 9.50E3
T: FTMS + c NSI d Full ms2 1113.97@hcd27.00 [100.00-2000.00]
Relative Abundance
m/z
418.1332 z=?
204.0864 z=1
366.1385 z=1
274.0946 z=?
454.1559 z=1
544.9109 z=?
639.2214 z=?
829.8174 z=?
962.8190 z=?
1113.5070 z=?
1205.6172 z=?
1306.6241 z=2
1408.6974 z=2
1570.7556 z=?
1724.0011 z=?
NLFLTNLDNLHENNTHNQEK

25-N

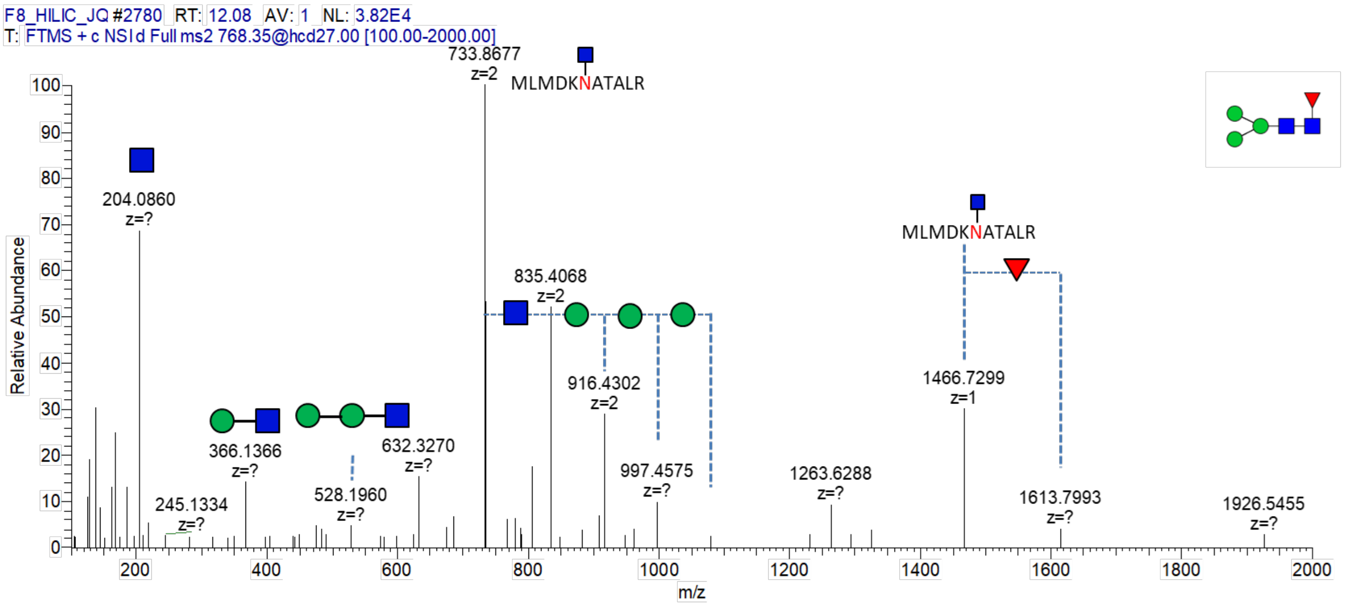

F8_HILIC_JQ #2780 RT: 12.08 AV: 1 NL: 3.82E4
T: FTMS + c NSI d Full ms2 768.35@hcd27.00 [100.00-2000.00]
733.8677
z=2
MLMDKNATALR
204.0860
z=?
835.4068
z=2
916.4302
z=2
1466.7299
z=1
366.1366
z=?
632.3270
z=?
528.1960
z=?
245.1334
z=?
997.4575
z=?
1263.6288
z=?
1613.7993
z=?
1926.5455
z=?
Relative Abundance
m/z

26-N

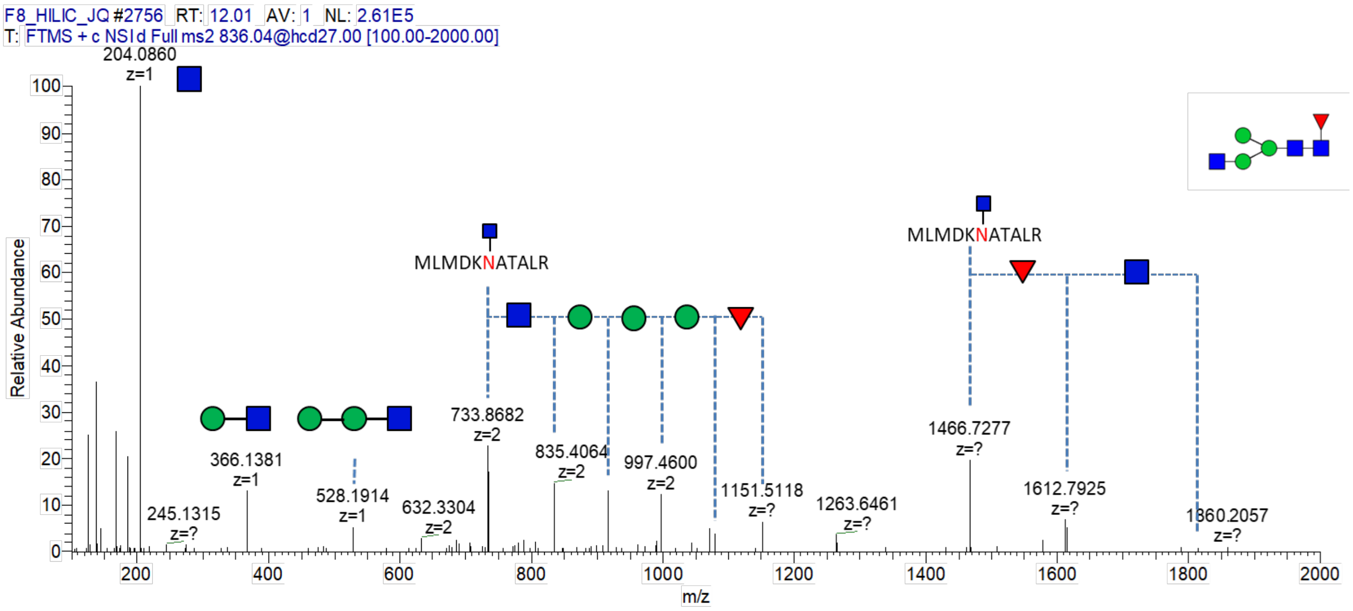

F8_HILIC_JQ #2756 RT: 12.01 AV: 1 NL: 2.61E5
T: FTMS + c NSI d Full ms2 836.04@hcd27.00 [100.00-2000.00]
204.0860
z=1
MLMDKNATALR
MLMDKNATALR
733.8682
z=2
366.1381
z=1
835.4064
z=2
997.4600
z=2
1466.7277
z=?
528.1914
z=1
632.3304
z=2
245.1315
z=?
1151.5118
z=?
1263.6461
z=?
1612.7925
z=?
1860.2057
z=?
Relative Abundance
m/z

27-N

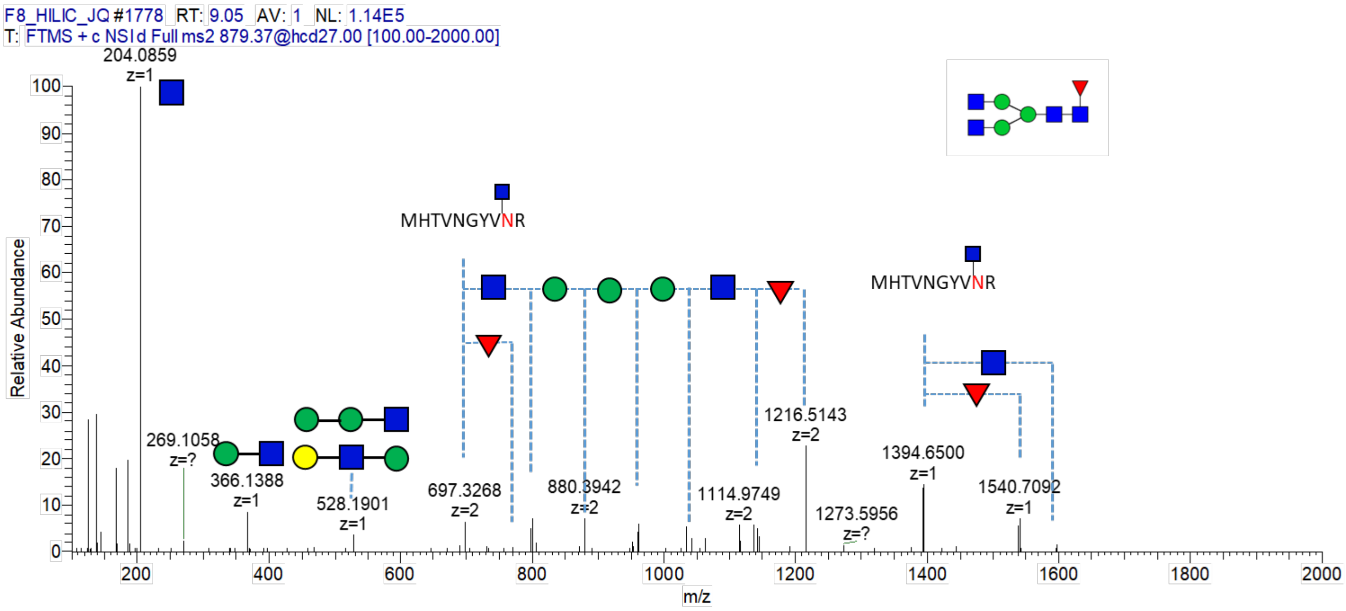

F8_HILIC_JQ #1778 RT: 9.05 AV: 1 NL: 1.14E5
T: FTMS + c NSI d Full ms2 879.37@hcd27.00 [100.00-2000.00]
204.0859
z=1
MHTVNGYVNR
MHTVNGYVNR
269.1058
z=?
366.1388
z=1
528.1901
z=1
697.3268
z=2
880.3942
z=2
1114.9749
z=2
1216.5143
z=2
1273.5956
z=?
1394.6500
z=1
1540.7092
z=1
Relative Abundance
m/z

28-N

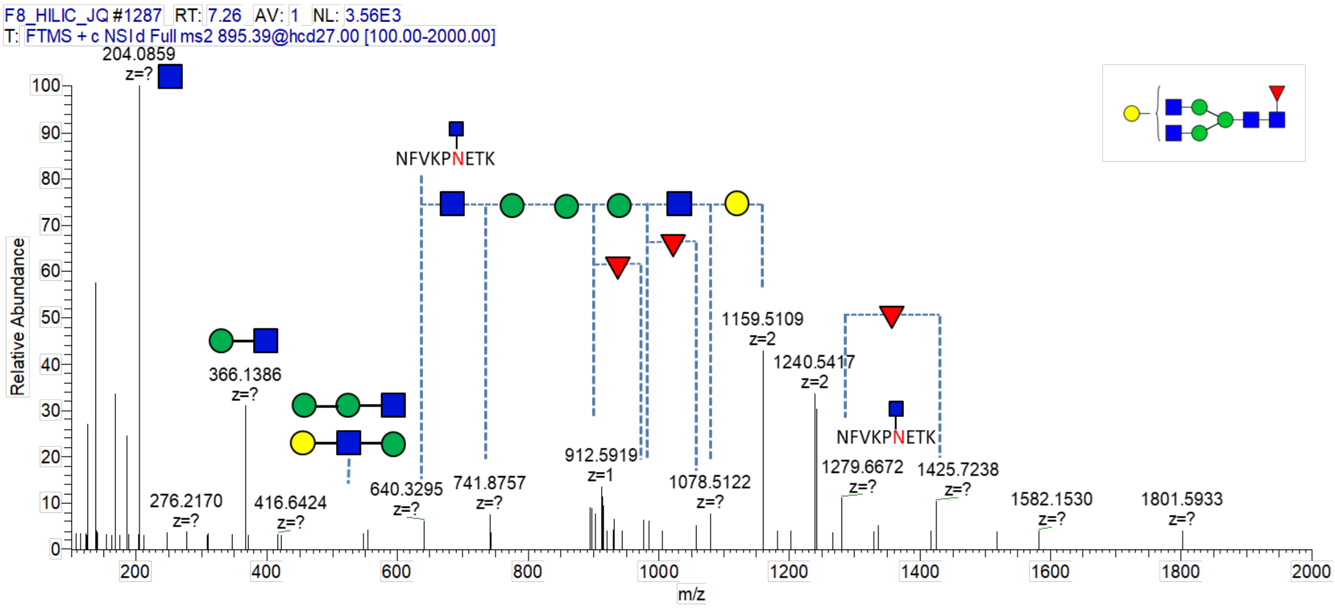

F8_HILIC_JQ #1287 RT: 7.26 AV: 1 NL: 3.56E3
T: FTMS + c NSI d Full ms2 895.39@hcd27.00 [100.00-2000.00]
204.0859
z=?
NFVKPNETK
366.1386
z=?
276.2170
z=?
416.6424
z=?
640.3295
z=?
741.8757
z=?
912.5919
z=1
1078.5122
z=?
1159.5109
z=2
1240.5417
z=2
NFVKPNETK
1279.6672
z=?
1425.7238
z=?
1582.1530
z=?
1801.5933
z=?
Relative Abundance
m/z

29-N

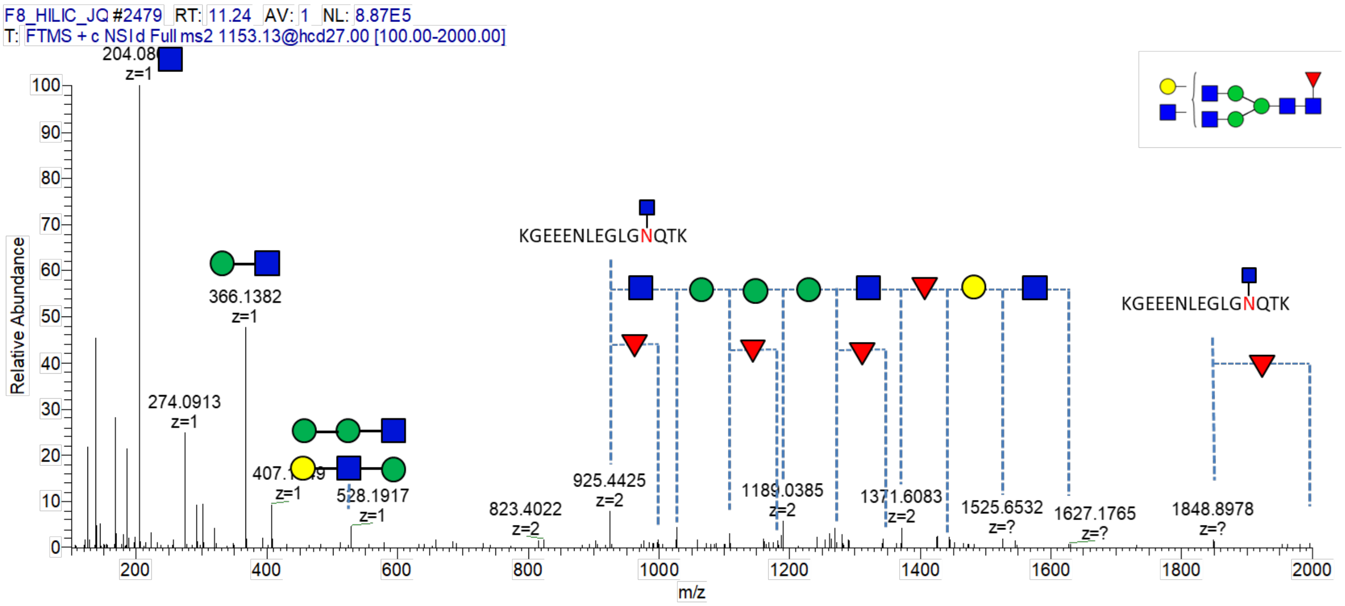
F8_HILIC_JQ #2479 RT: 11.24 AV: 1 NL: 8.87E5
T: FTMS + c NSI d Full ms2 1153.13@hcd27.00 [100.00-2000.00]
Relative Abundance
m/z
z=1
366.1382
z=1
274.0913
z=1
z=1
528.1917
z=1
KGEEENLEGLGNQTK
823.4022
z=2
925.4425
z=2
z=2
z=2
1525.6532
z=?
1627.1765
z=?
KGEEENLEGLGNQTK
1848.8978
z=?

30-N

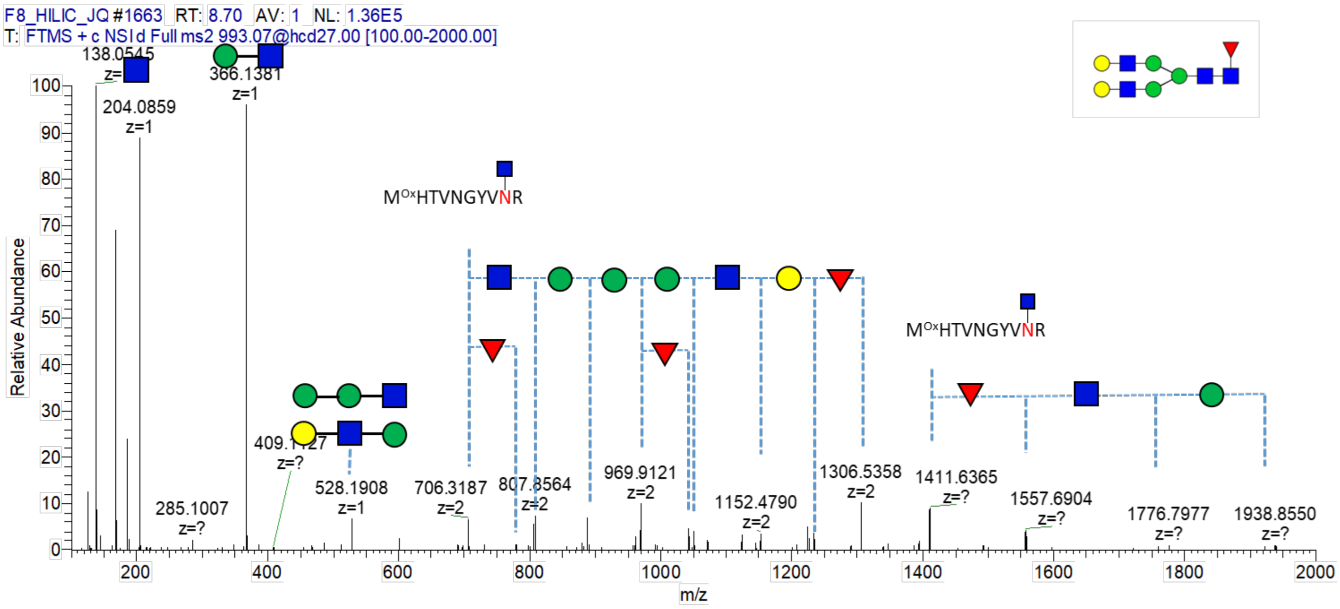
F8_HILIC_JQ #1663 RT: 8.70 AV: 1 NL: 1.36E5
T: FTMS + c NSI d Full ms2 993.07@hcd27.00 [100.00-2000.00]
Relative Abundance
m/z
138.0545
204.0859
z=1
366.1381
z=1
MOxHTVNGYVNR
285.1007
z=?
z=?
528.1908
z=1
706.3187
z=2
969.9121
z=2
1152.4790
z=2
1306.5358
z=2
MOxHTVNGYVNR
1411.6365
z=?
1557.6904
z=?
1776.7977
z=?
1938.8550
z=?

31-N

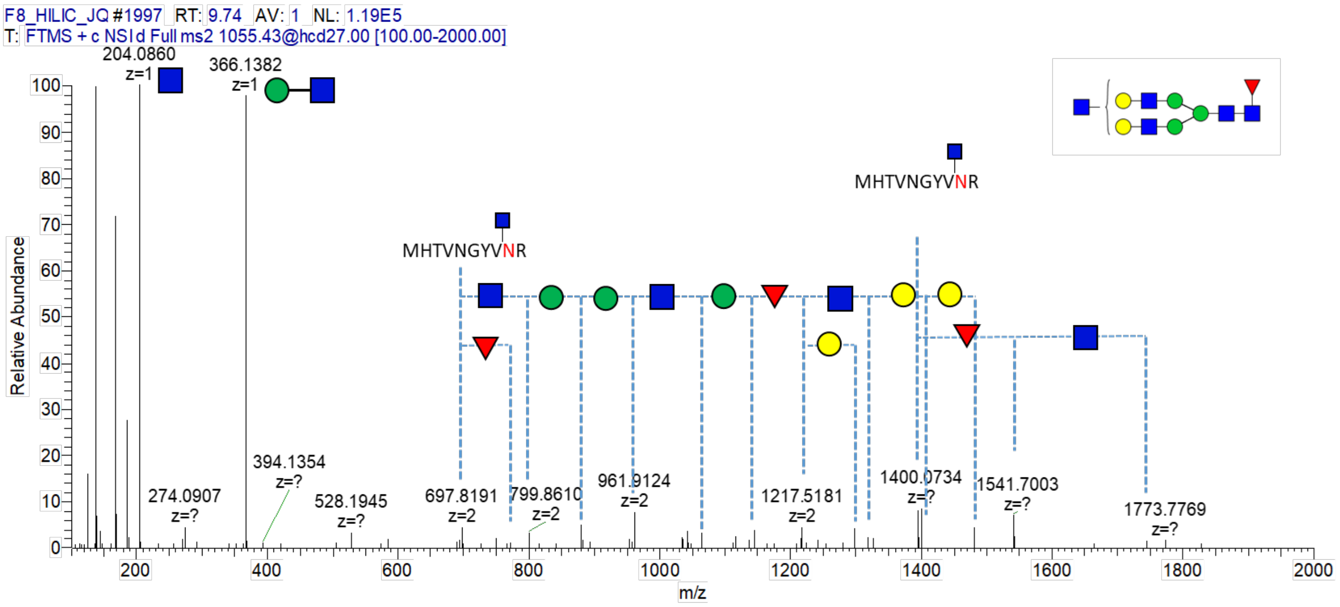
F8_HILIC_JQ #1997 RT: 9.74 AV: 1 NL: 1.19E5
T: FTMS + c NSI d Full ms2 1055.43@hcd27.00 [100.00-2000.00]
204.0860 z=1
366.1382 z=1
MHTVNGYVNR
MHTVNGYVNR
274.0907 z=?
394.1354 z=?
528.1945 z=?
697.8191 z=2
799.8610 z=2
961.9124 z=2
1217.5181 z=2
1400.0734 z=?
1541.7003 z=?
1773.7769 z=?
Relative Abundance
m/z

32-N

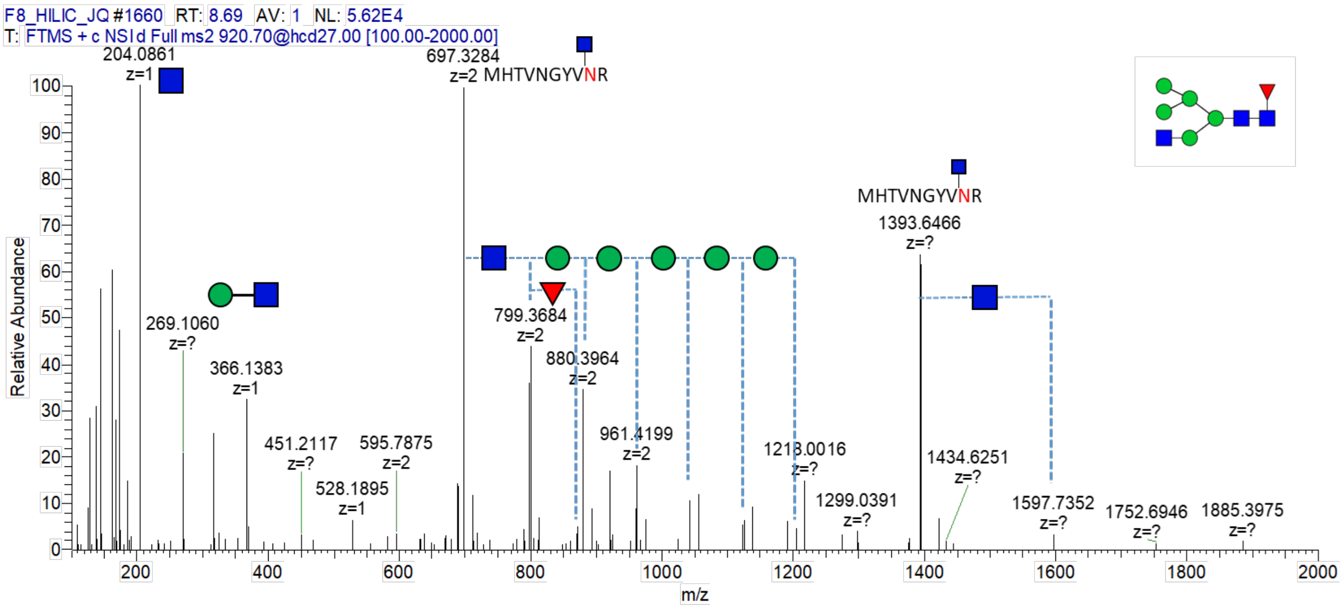
F8_HILIC_JQ #1660 RT: 8.69 AV: 1 NL: 5.62E4
T: FTMS + c NSI d Full ms2 920.70@hcd27.00 [100.00-2000.00]
204.0861 z=1
697.3284 z=2 MHTVNGYVNR
MHTVNGYVNR
1393.6466 z=?
269.1060 z=?
366.1383 z=1
799.3684 z=2
880.3964 z=2
451.2117 z=?
595.7875 z=2
528.1895 z=1
961.4199 z=2
1218.0016 z=?
1299.0391 z=?
1434.6251 z=?
1597.7352 z=?
1752.6946 z=?
1885.3975 z=?
Relative Abundance
m/z

33-N

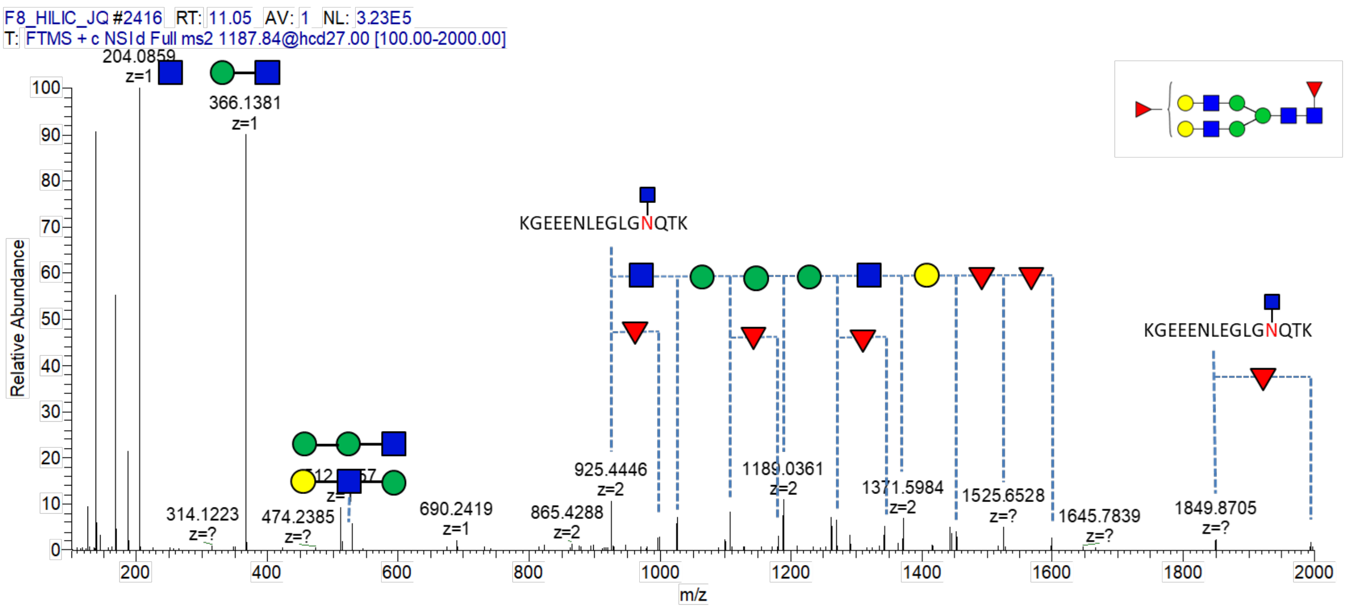

F8_HILIC_JQ #2416 RT: 11.05 AV: 1 NL: 3.23E5
T: FTMS + c NSI d Full ms2 1187.84@hcd27.00 [100.00-2000.00]
204.0859 z=1
366.1381 z=1
314.1223 z=?
474.2385 z=?
690.2419 z=1
865.4288 z=2
925.4446 z=2
1189.0361 z=2
1371.5984 z=2
1525.6528 z=?
1645.7839 z=?
1849.8705 z=?
KGEEENLEGLGNQTK
KGEEENLEGLGNQTK
Relative Abundance
m/z

34-N

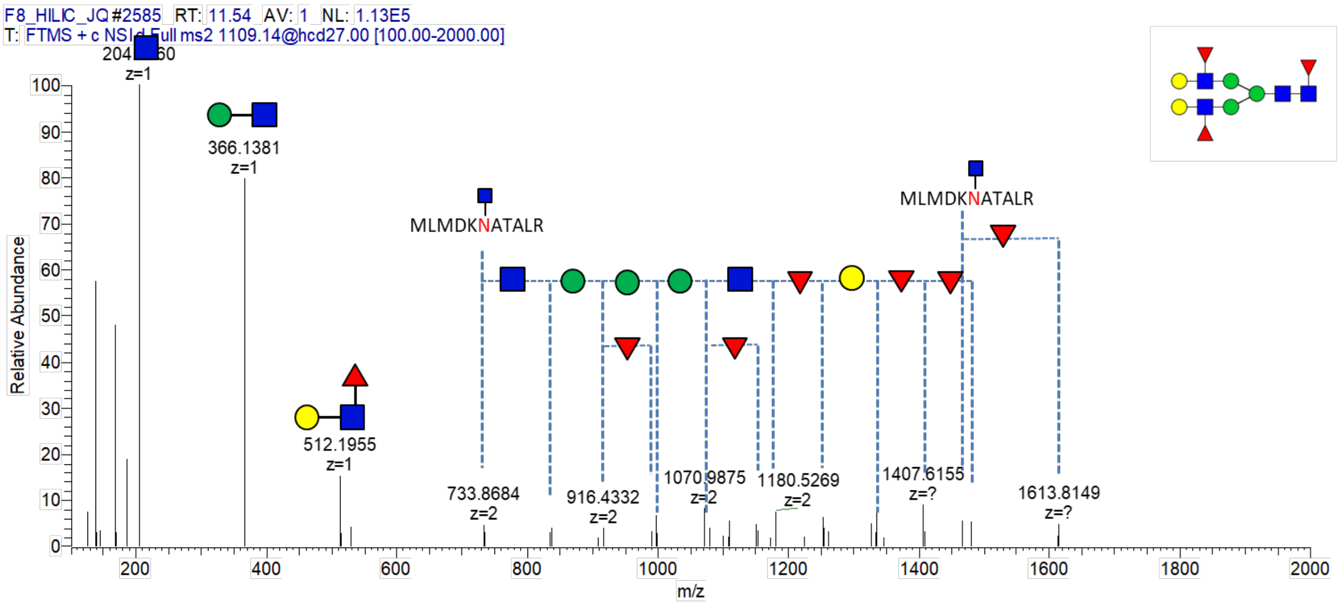

F8_HILIC_JQ #2585 RT: 11.54 AV: 1 NL: 1.13E5
T: FTMS + c NSI d Full ms2 1109.14@hcd27.00 [100.00-2000.00]
z=1
366.1381 z=1
512.1955 z=1
733.8684 z=2
916.4332 z=2
1070.9875 z=2
1180.5269 z=2
1407.6155 z=?
1613.8149 z=?
MLMDKNATALR
MLMDKNATALR
Relative Abundance
m/z

35-N

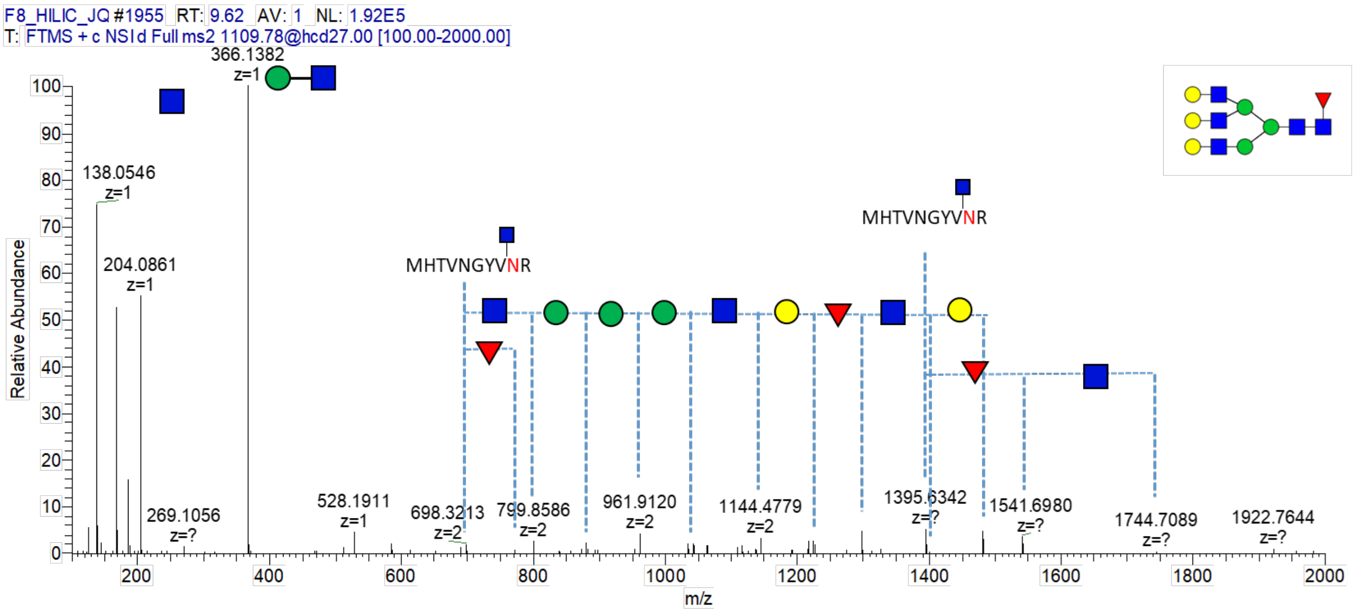
F8_HILIC_JQ #1955 RT: 9.62 AV: 1 NL: 1.92E5
T: FTMS + c NSI d Full ms2 1109.78@hcd27.00 [100.00-2000.00]
366.1382
z=1
138.0546
z=1
204.0861
z=1
269.1056
z=?
528.1911
z=1
698.3213
z=2
799.8586
z=2
961.9120
z=2
1144.4779
z=2
1395.6342
z=?
1541.6980
z=?
1744.7089
z=?
1922.7644
z=?
MHTVNGYVNR
MHTVNGYVNR
Relative Abundance
m/z

36-N

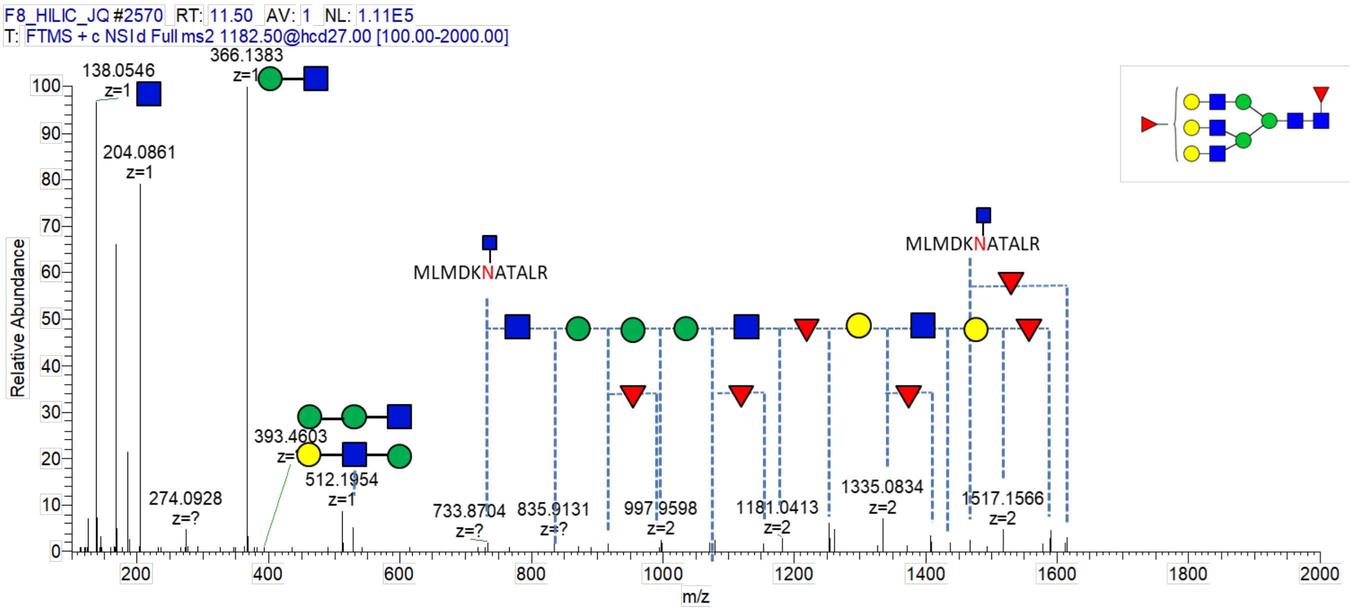
F8_HILIC_JQ #2570 RT: 11.50 AV: 1 NL: 1.11E5
T: FTMS + c NSI d Full ms2 1182.50@hcd27.00 [100.00-2000.00]
366.1383
z=1
138.0546
z=1
204.0861
z=1
274.0928
z=?
393.4603
z=
512.1954
z=1
733.8704
z=?
835.9131
z=?
997.9598
z=2
1181.0413
z=2
1335.0834
z=2
1517.1566
z=2
MLMDKNATALR
MLMDKNATALR
Relative Abundance
m/z

37-N

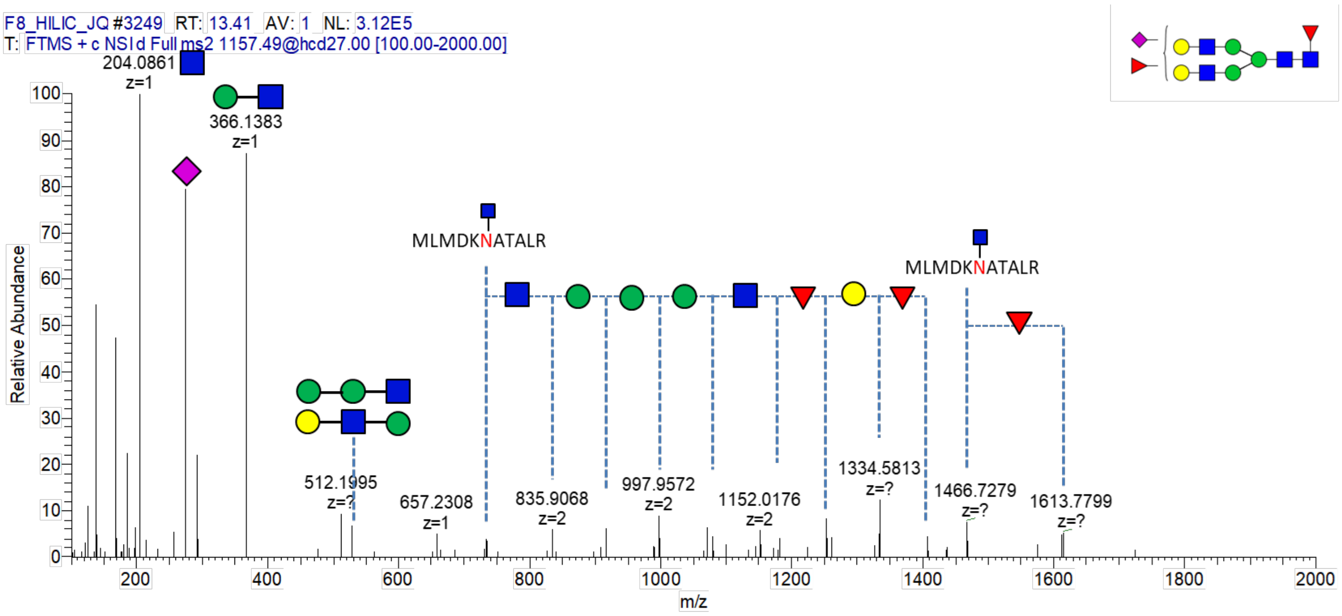
F8_HILIC_JQ #3249 RT: 13.41 AV: 1 NL: 3.12E5
T: FTMS + c NSI d Full ms2 1157.49@hcd27.00 [100.00-2000.00]
204.0861
z=1
366.1383
z=1
MLMDKNATALR
MLMDKNATALR
512.1995
z=?
657.2308
z=1
835.9068
z=2
997.9572
z=2
1152.0176
z=2
1334.5813
z=?
1466.7279
z=?
1613.7799
z=?
Relative Abundance
m/z

38-N

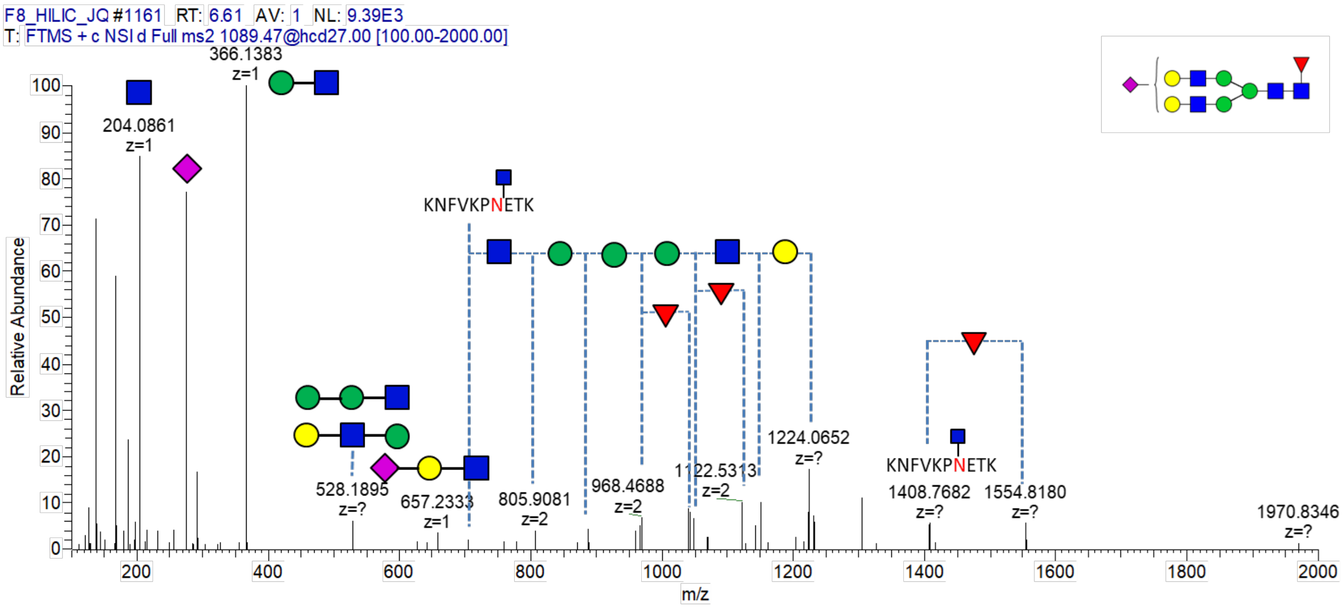
F8_HILIC_JQ #1161 RT: 6.61 AV: 1 NL: 9.39E3
T: FTMS + c NSI d Full ms2 1089.47@hcd27.00 [100.00-2000.00]
366.1383
z=1
204.0861
z=1
KNFVKPNETK
528.1895
z=?
657.2333
z=1
805.9081
z=2
968.4688
z=2
1122.5313
z=2
1224.0652
z=?
KNFVKPNETK
1408.7682
z=?
1554.8180
z=?
1970.8346
z=?
Relative Abundance
m/z

39-N

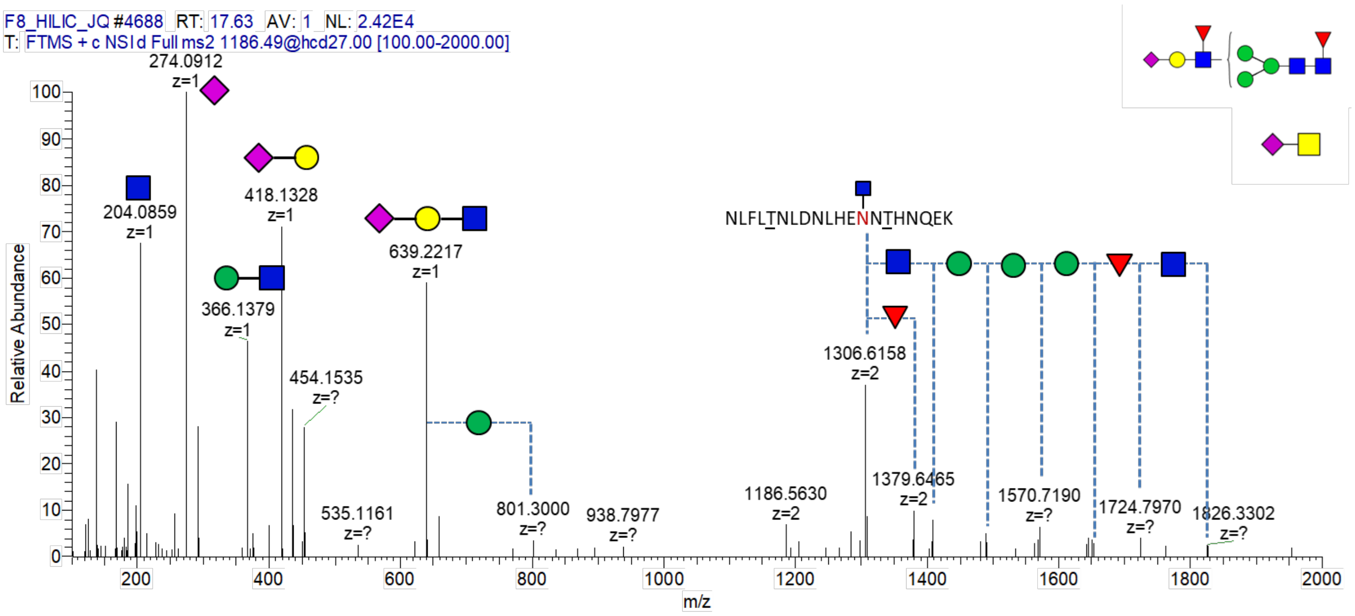

F8_HILIC_JQ #4688 RT: 17.63 AV: 1 NL: 2.42E4
T: FTMS + c NSI d Full ms2 1186.49@hcd27.00 [100.00-2000.00]
274.0912 z=1
204.0859 z=1
418.1328 z=1
366.1379 z=1
454.1535 z=?
639.2217 z=1
535.1161 z=?
801.3000 z=?
938.7977 z=?
1186.5630 z=2
NLFLTNLDNLHENNTHNQEK
1306.6158 z=2
1379.6465 z=2
1570.7190 z=?
1724.7970 z=?
1826.3302 z=?
Relative Abundance
m/z

40-N

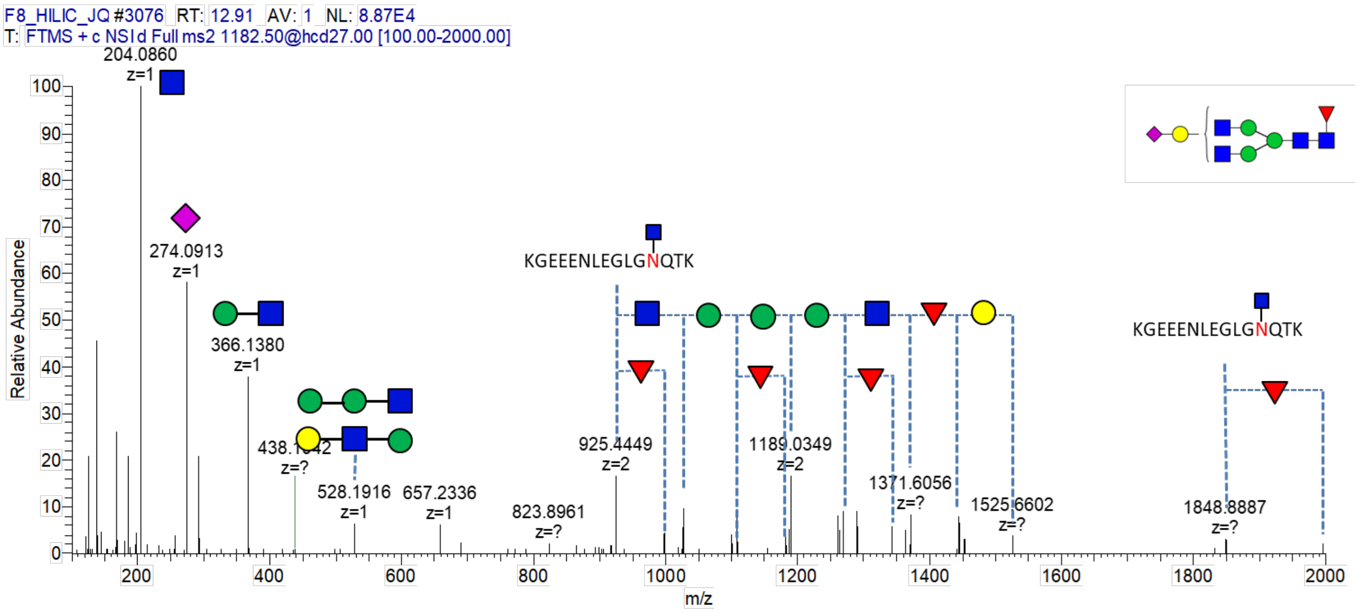

F8_HILIC_JQ #3076 RT: 12.91 AV: 1 NL: 8.87E4
T: FTMS + c NSI d Full ms2 1182.50@hcd27.00 [100.00-2000.00]
204.0860 z=1
274.0913 z=1
366.1380 z=1
438.1642 z=?
528.1916 z=1
657.2336 z=1
823.8961 z=?
KGEEENLEGLGNQTK
925.4449 z=2
1189.0349 z=2
1371.6056 z=?
1525.6602 z=?
KGEEENLEGLGNQTK
1848.8887 z=?
Relative Abundance
m/z

41-N

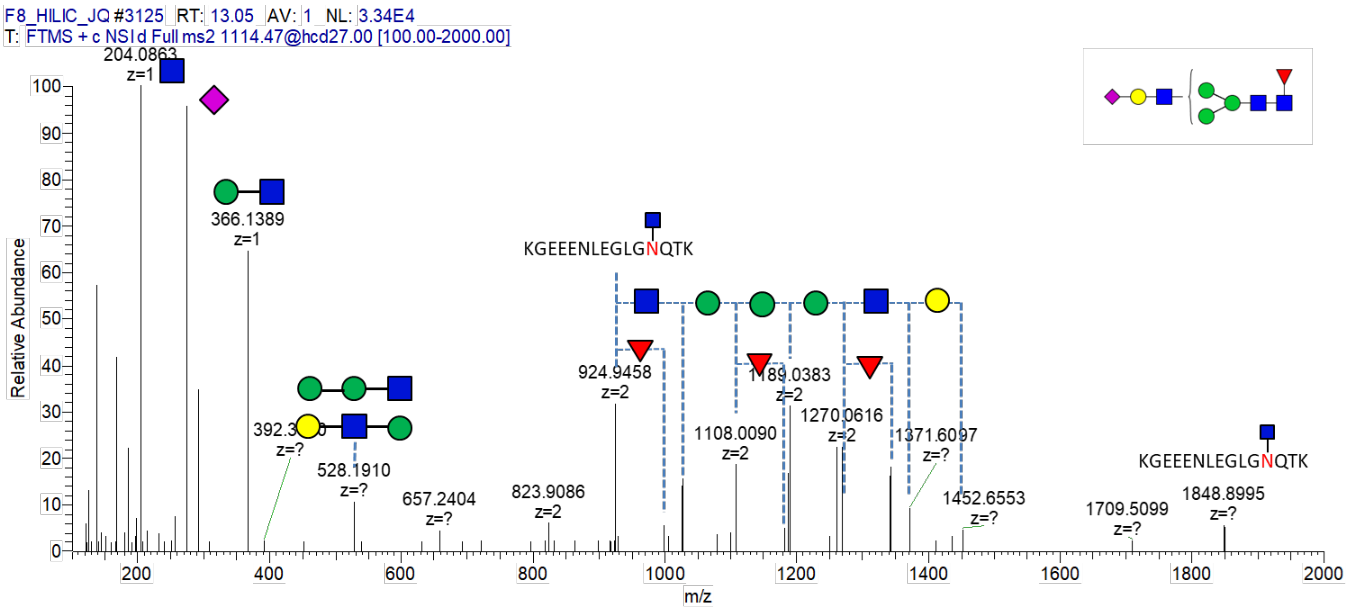
F8_HILIC_JQ #3125 RT: 13.05 AV: 1 NL: 3.34E4
T: FTMS + c NSI d Full ms2 1114.47@hcd27.00 [100.00-2000.00]
Relative Abundance
m/z
204.0863
z=1
366.1389
z=1
392.3
z=?
528.1910
z=?
657.2404
z=?
823.9086
z=2
KGEEENLEGLGNQTK
924.9458
z=2
1108.0090
z=2
1189.0383
z=2
1270.0616
z=2
1371.6097
z=?
1452.6553
z=?
1709.5099
z=?
1848.8995
z=?
KGEEENLEGLGNQTK

42-N

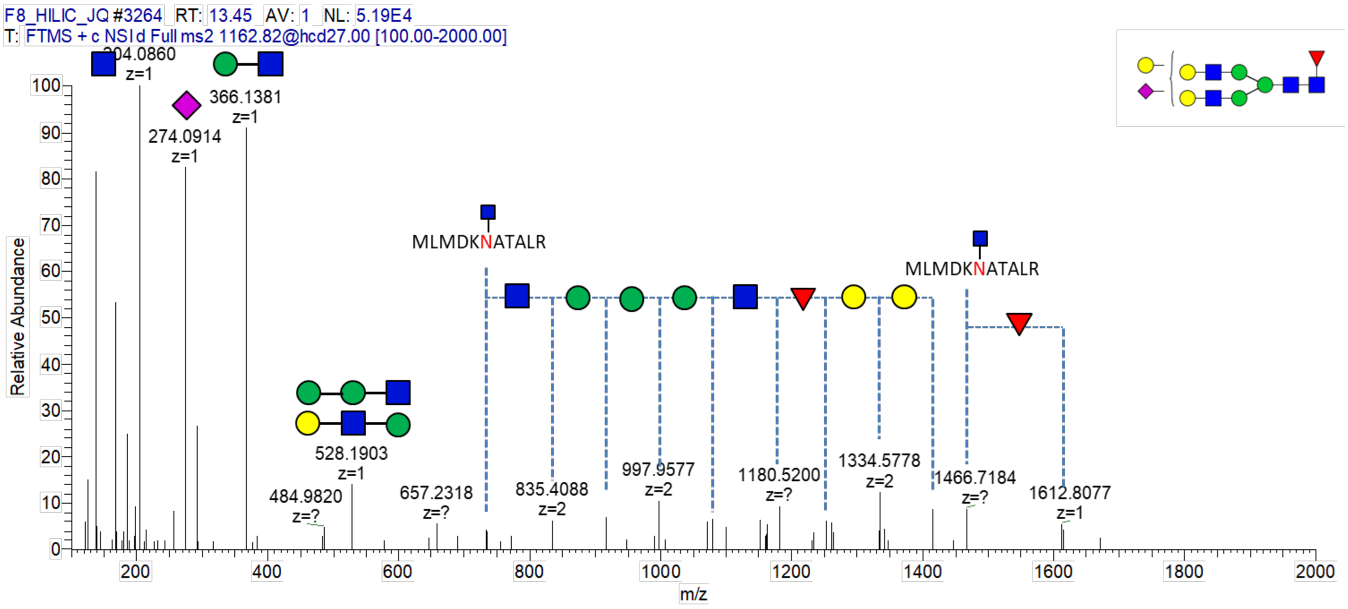
F8_HILIC_JQ #3264 RT: 13.45 AV: 1 NL: 5.19E4
T: FTMS + c NSI d Full ms2 1162.82@hcd27.00 [100.00-2000.00]
Relative Abundance
m/z
204.0860
z=1
366.1381
z=1
274.0914
z=1
528.1903
z=1
484.9820
z=?
657.2318
z=?
MLMDKNATALR
835.4088
z=2
997.9577
z=2
1180.5200
z=?
1334.5778
z=2
MLMDKNATALR
1466.7184
z=?
1612.8077
z=1

43-N

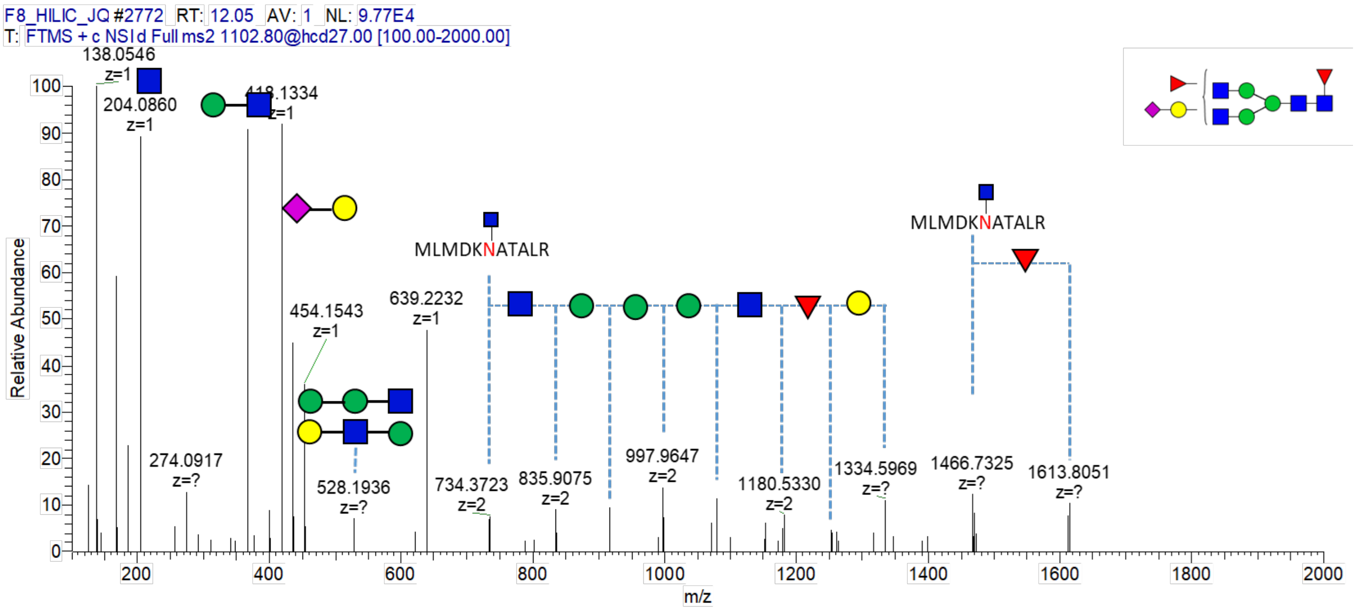
F8_HILIC_JQ #2772 RT: 12.05 AV: 1 NL: 9.77E4
T: FTMS + c NSI d Full ms2 1102.80@hcd27.00 [100.00-2000.00]
138.0546 z=1
204.0860 z=1
274.0917 z=?
454.1543 z=1
528.1936 z=?
639.2232 z=1
MLMDKNATALR
734.3723 z=2
835.9075 z=2
997.9647 z=2
1180.5330 z=2
1334.5969 z=?
1466.7325 z=?
1613.8051 z=?
Relative Abundance
m/z

44-N

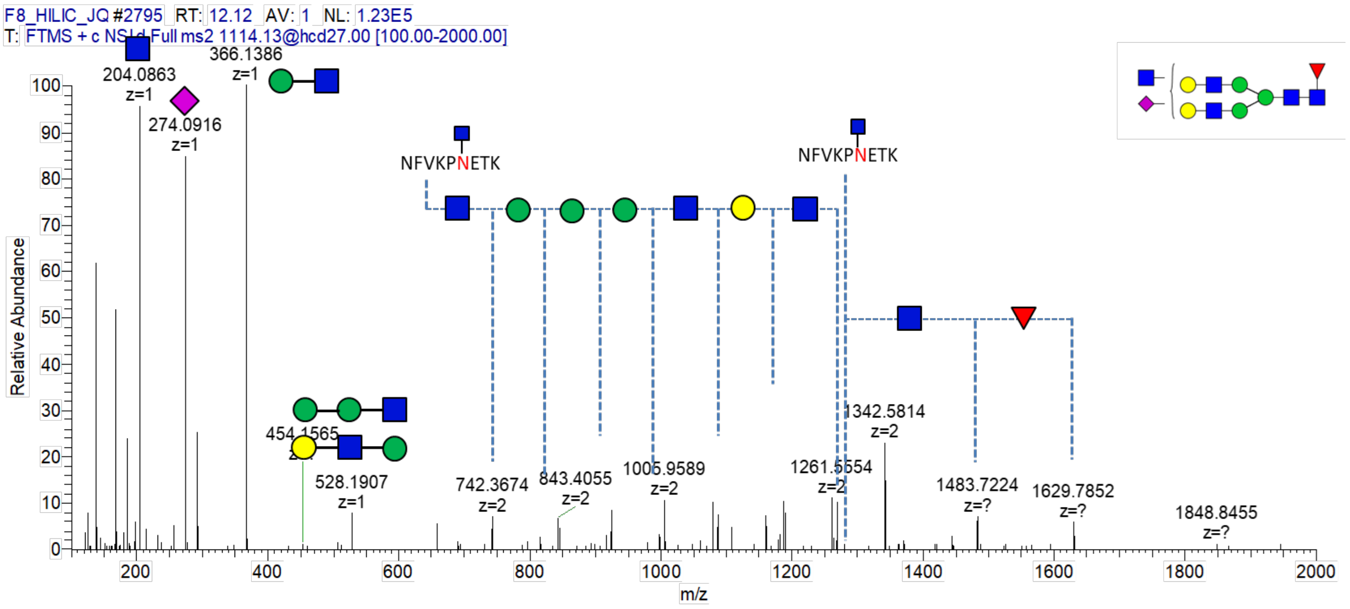
F8_HILIC_JQ #2795 RT: 12.12 AV: 1 NL: 1.23E5
T: FTMS + c NSI d Full ms2 1114.13@hcd27.00 [100.00-2000.00]
204.0863 z=1
274.0916 z=1
366.1386 z=1
528.1907 z=1
NFVKPNETK
742.3674 z=2
843.4055 z=2
1005.9589 z=2
1261.5554 z=2
1342.5814 z=2
1483.7224 z=?
1629.7852 z=?
1848.8455 z=?
Relative Abundance
m/z

45-N

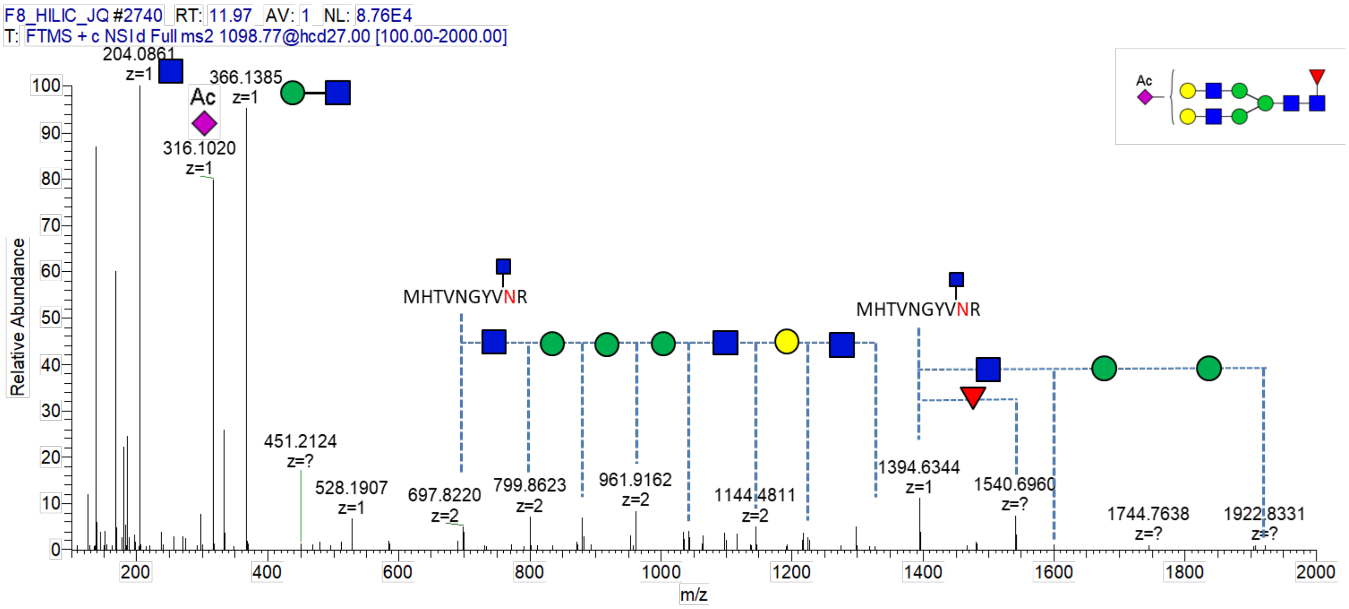

F8_HILIC_JQ #2740 RT: 11.97 AV: 1 NL: 8.76E4
T: FTMS + c NSI d Full ms2 1098.77@hcd27.00 [100.00-2000.00]
Relative Abundance
m/z
204.0861 z=1
366.1385 z=1
Ac
316.1020 z=1
451.2124 z=?
528.1907 z=1
697.8220 z=2
799.8623 z=2
961.9162 z=2
1144.4811 z=2
1394.6344 z=1
1540.6960 z=?
1744.7638 z=?
1922.8331 z=?
MHTVNGYVNR
MHTVNGYVNR
Ac

46-N

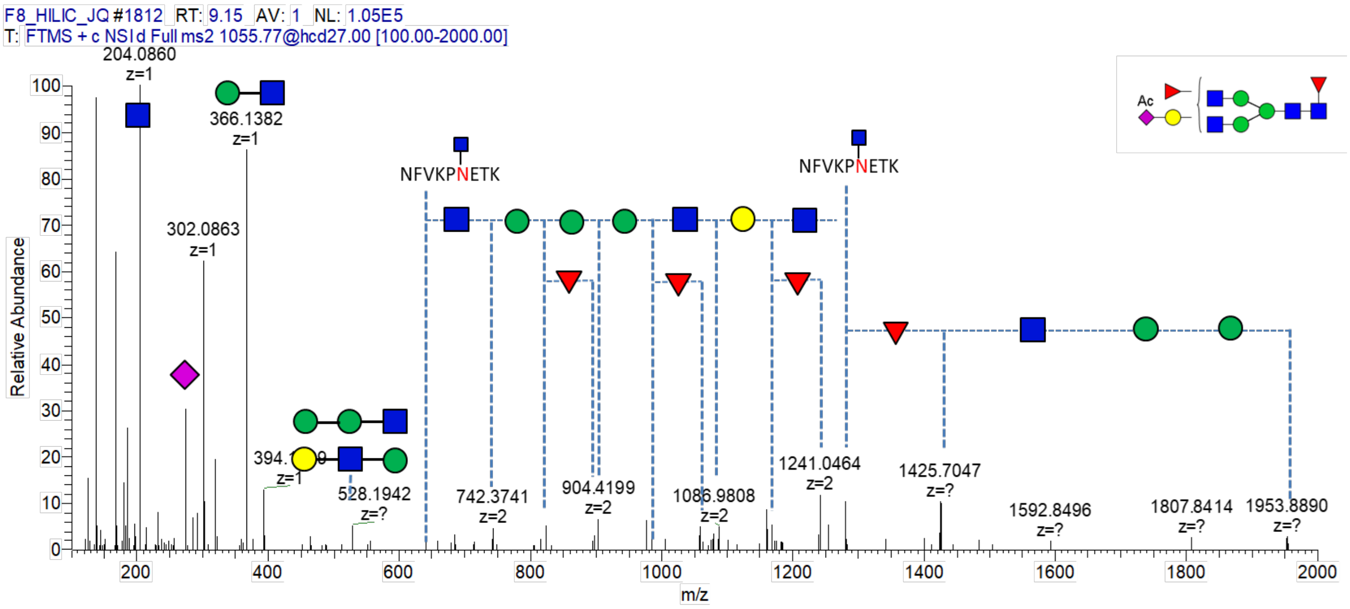

F8_HILIC_JQ #1812 RT: 9.15 AV: 1 NL: 1.05E5
T: FTMS + c NSI d Full ms2 1055.77@hcd27.00 [100.00-2000.00]
Relative Abundance
m/z
204.0860 z=1
366.1382 z=1
302.0863 z=1
z=1
528.1942 z=?
742.3741 z=2
904.4199 z=2
1086.9808 z=2
1241.0464 z=2
1425.7047 z=?
1592.8496 z=?
1807.8414 z=?
1953.8890 z=?
NFVKPNETK
NFVKPNETK
Ac

47-N

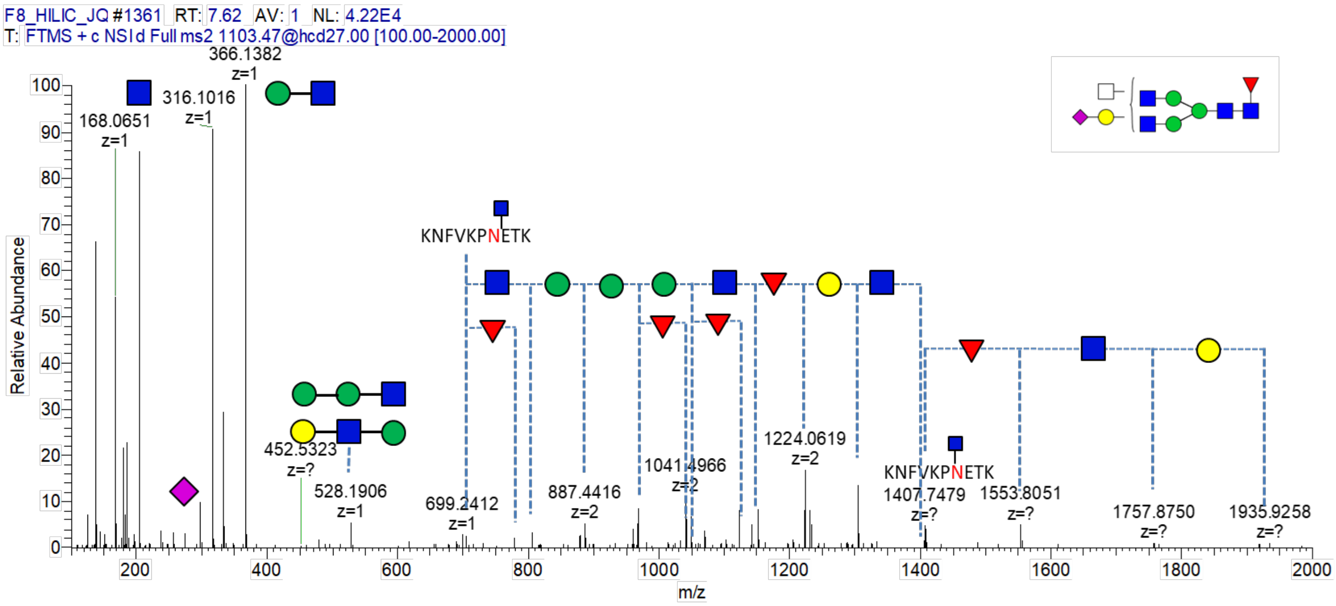
F8_HILIC_JQ #1361 RT: 7.62 AV: 1 NL: 4.22E4
T: FTMS + c NSI d Full ms2 1103.47@hcd27.00 [100.00-2000.00]
366.1382 z=1
316.1016 z=1
168.0651 z=1
452.5323 z=?
528.1906 z=1
KNFVKPNETK
699.2412 z=1
887.4416 z=2
1041.4966 z=2
1224.0619 z=2
KNFVKPNETK
1407.7479 z=?
1553.8051 z=?
1757.8750 z=?
1935.9258 z=?
Relative Abundance
m/z

48-N

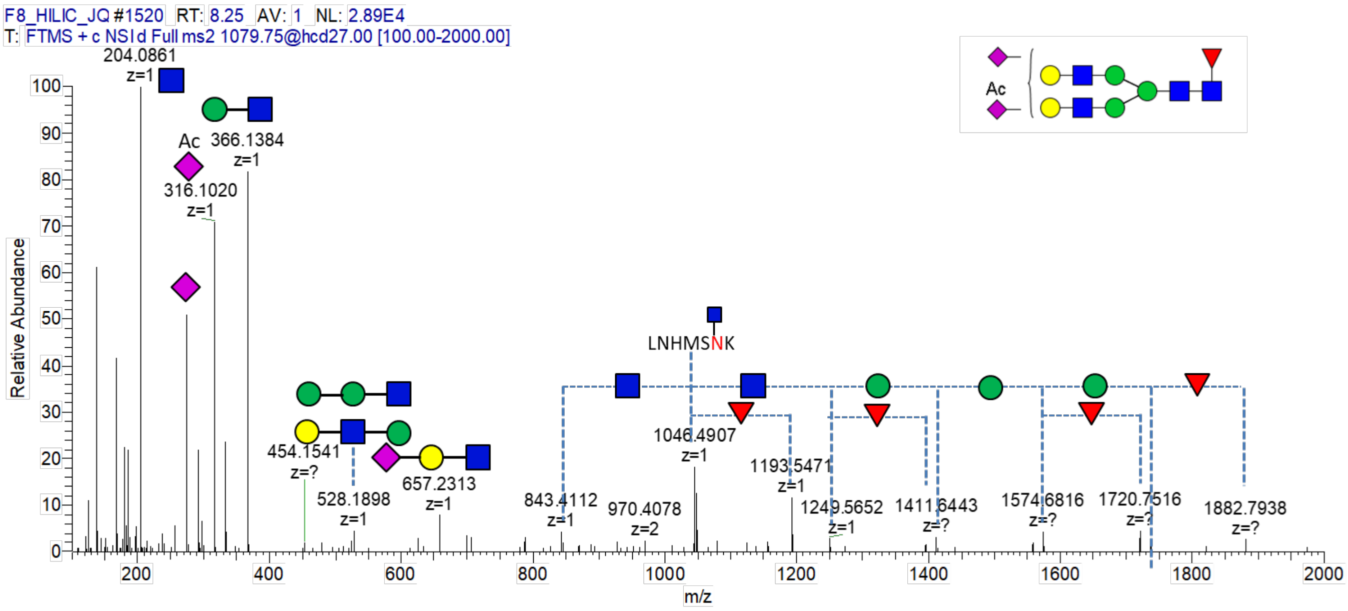
F8_HILIC_JQ #1520 RT: 8.25 AV: 1 NL: 2.89E4
T: FTMS + c NSI d Full ms2 1079.75@hcd27.00 [100.00-2000.00]
204.0861 z=1
Ac
366.1384 z=1
316.1020 z=1
454.1541 z=?
528.1898 z=1
657.2313 z=1
LNHMSNK
843.4112 z=1
970.4078 z=2
1046.4907 z=1
1193.5471 z=1
1249.5652 z=1
1411.6443 z=?
1574.6816 z=?
1720.7516 z=?
1882.7938 z=?
Relative Abundance
m/z

49-N

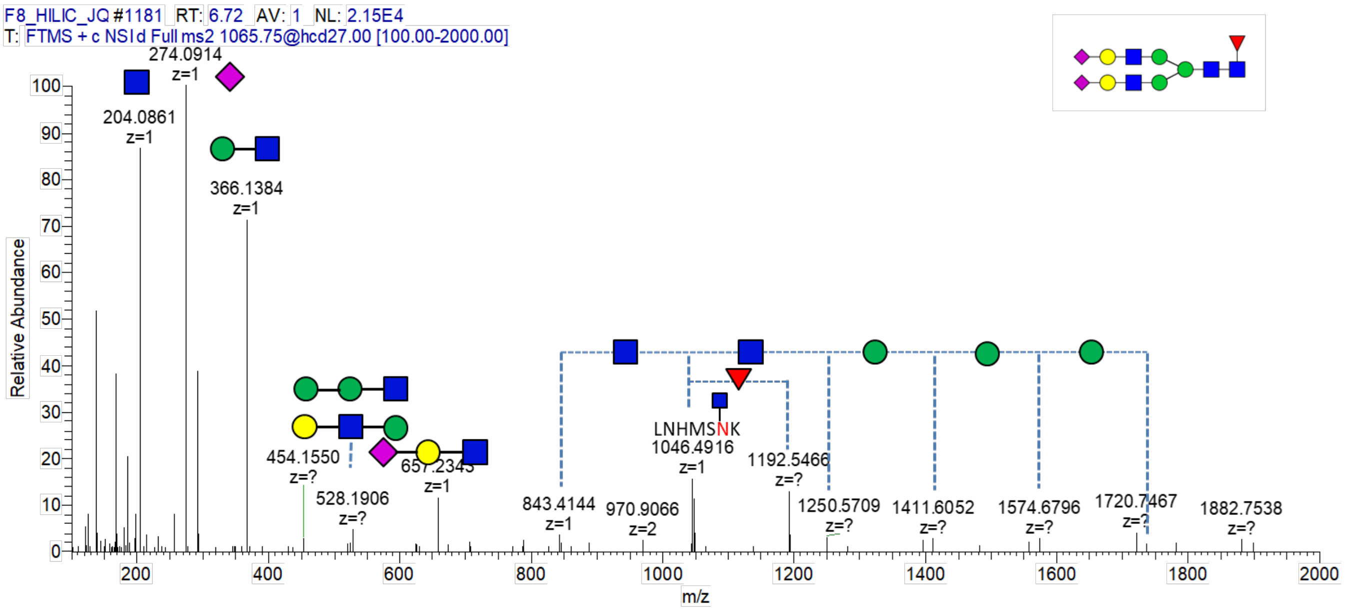
F8_HILIC_JQ #1181 RT: 6.72 AV: 1 NL: 2.15E4
T: FTMS + c NSI d Full ms2 1065.75@hcd27.00 [100.00-2000.00]
Relative Abundance
m/z
204.0861 z=1
274.0914 z=1
366.1384 z=1
454.1550 z=?
528.1906 z=?
657.2343 z=1
843.4144 z=1
970.9066 z=2
LNHMSNK
1046.4916 z=1
1192.5466 z=?
1250.5709 z=?
1411.6052 z=?
1574.6796 z=?
1720.7467 z=?
1882.7538 z=?

50-N

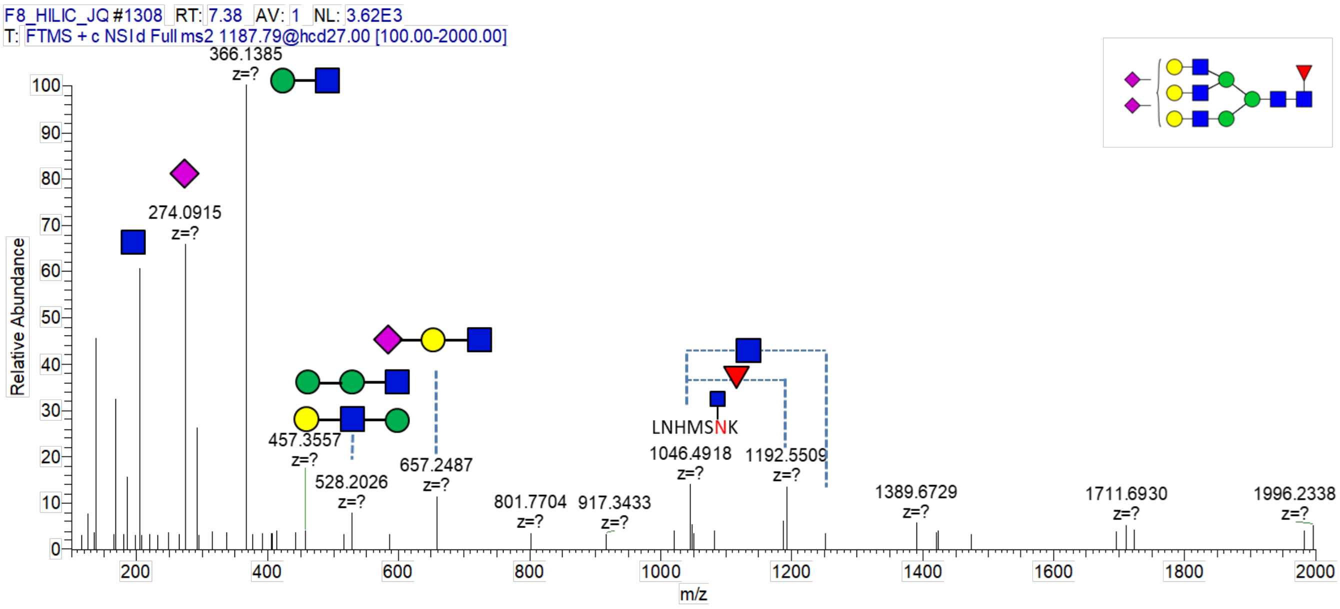
F8_HILIC_JQ #1308 RT: 7.38 AV: 1 NL: 3.62E3
T: FTMS + c NSI d Full ms2 1187.79@hcd27.00 [100.00-2000.00]
Relative Abundance
m/z
366.1385 z=?
274.0915 z=?
457.3557 z=?
528.2026 z=?
657.2487 z=?
801.7704 z=?
917.3433 z=?
LNHMSNK
1046.4918 z=?
1192.5509 z=?
1389.6729 z=?
1711.6930 z=?
1996.2338 z=?

51-N

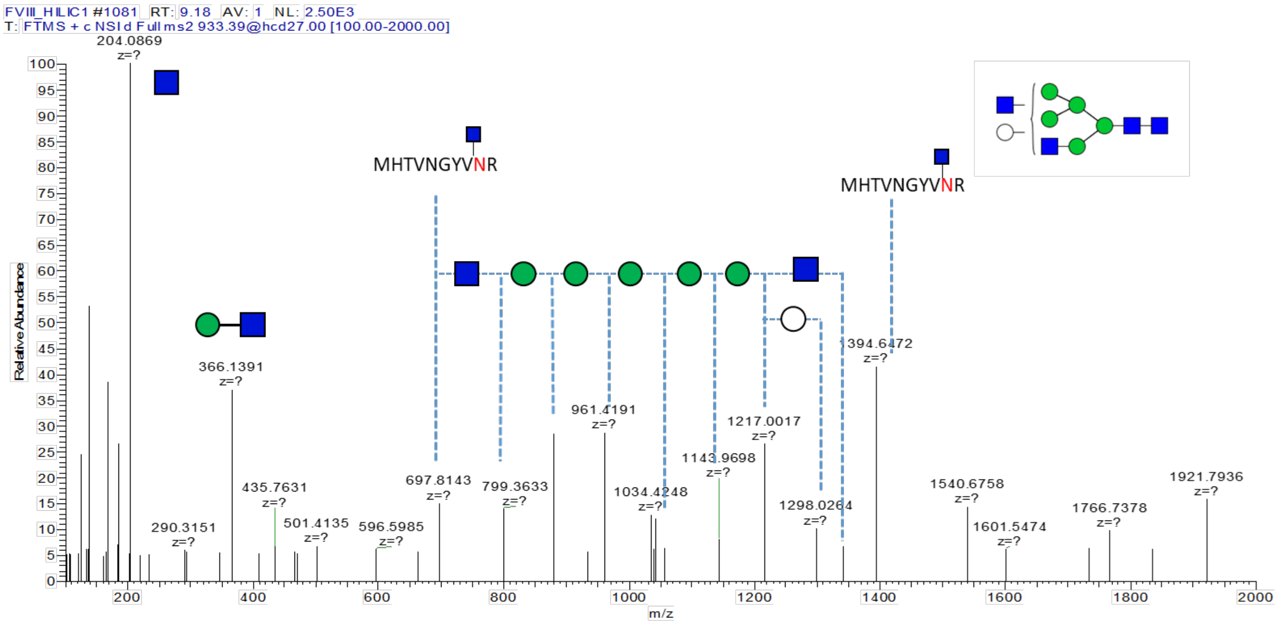

FVIII_HILIC1 #1081 RT: 9.18 AV: 1 NL: 2.50E3
T: FTMS + c NSI d Full ms2 933.39@hcd27.00 [100.00-2000.00]
204.0869
366.1391
290.3151
435.7631
501.4135
596.5985
697.8143
799.3633
961.4191
1034.4248
1143.9698
1217.0017
1298.0264
1394.6472
1540.6758
1601.5474
1766.7378
1921.7936
MHTVNGYVNR
MHTVNGYVNR
Relative Abundance
m/z

52-N

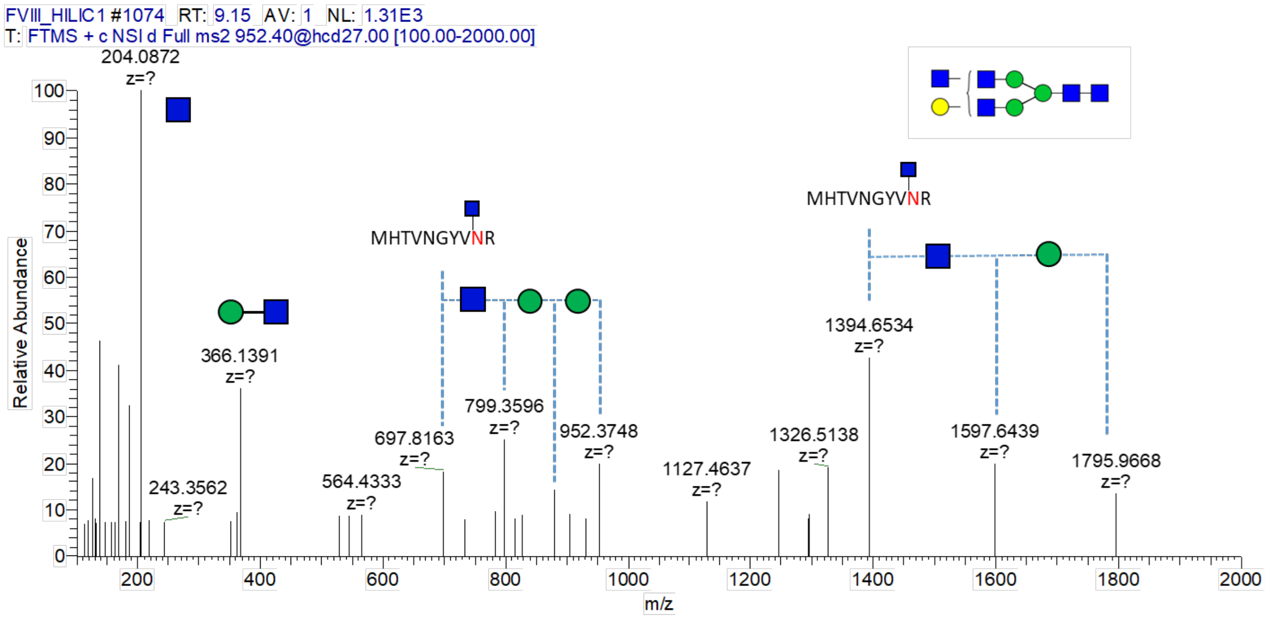

FVIII_HILIC1 #1074 RT: 9.15 AV: 1 NL: 1.31E3
T: FTMS + c NSI d Full ms2 952.40@hcd27.00 [100.00-2000.00]
204.0872
366.1391
243.3562
564.4333
697.8163
799.3596
952.3748
1127.4637
1326.5138
1394.6534
1597.6439
1795.9668
MHTVNGYVNR
MHTVNGYVNR
Relative Abundance
m/z

53-N

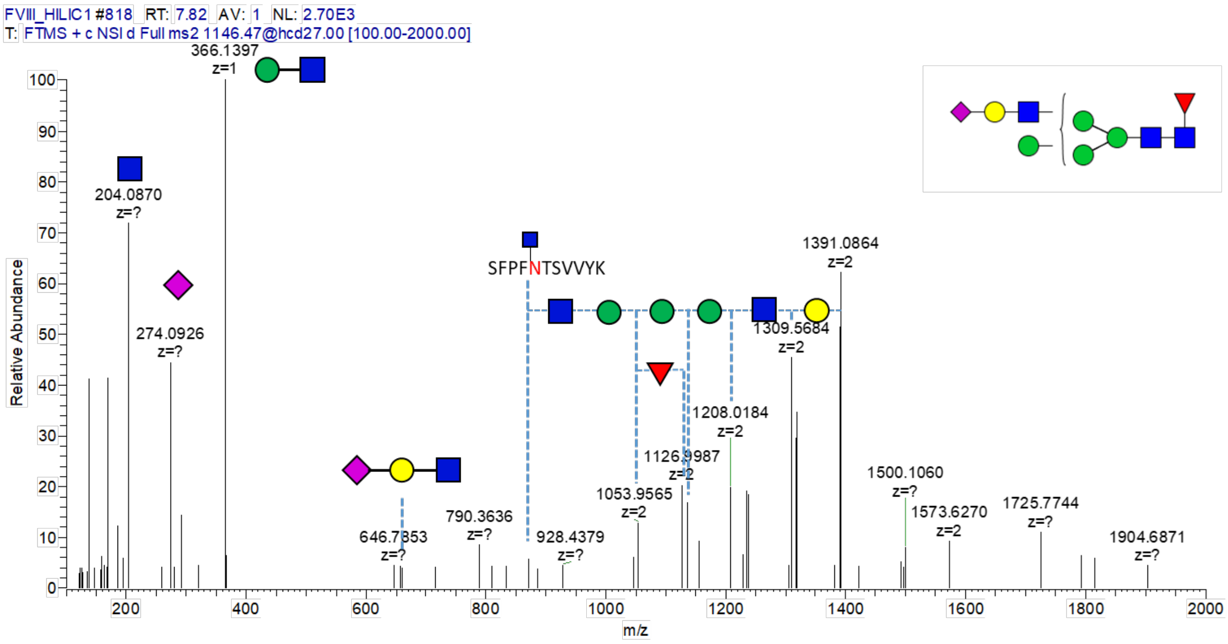

FVIII_HILIC1 #818 RT: 7.82 AV: 1 NL: 2.70E3
T: FTMS + c NSI d Full ms2 1146.47@hcd27.00 [100.00-2000.00]
Relative Abundance
m/z
366.1397 z=1
204.0870 z=?
274.0926 z=?
SFPFNTSVVYK
1391.0864 z=2
1309.5684 z=2
1208.0184 z=2
1126.9987 z=2
1053.9565 z=2
646.7853 z=?
790.3636 z=?
928.4379 z=?
1500.1060 z=?
1573.6270 z=2
1725.7744 z=?
1904.6871 z=?

54-N

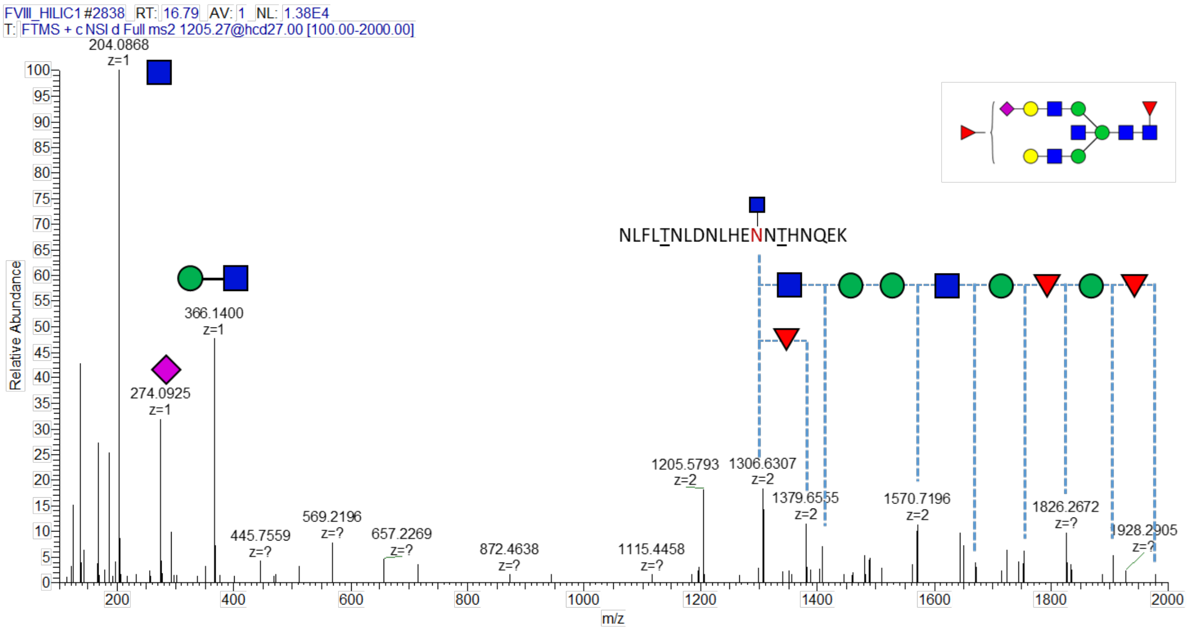

FVIII_HILIC1 #2838 RT: 16.79 AV: 1 NL: 1.38E4
T: FTMS + c NSI d Full ms2 1205.27@hcd27.00 [100.00-2000.00]
Relative Abundance
m/z
204.0868 z=1
366.1400 z=1
274.0925 z=1
NLFLTNLDNLHENNTHNQEK
445.7559 z=?
569.2196 z=?
657.2269 z=?
872.4638 z=?
1115.4458 z=?
1205.5793 z=2
1306.6307 z=2
1379.6555 z=2
1570.7196 z=2
1826.2672 z=?
1928.2905 z=?

55-N

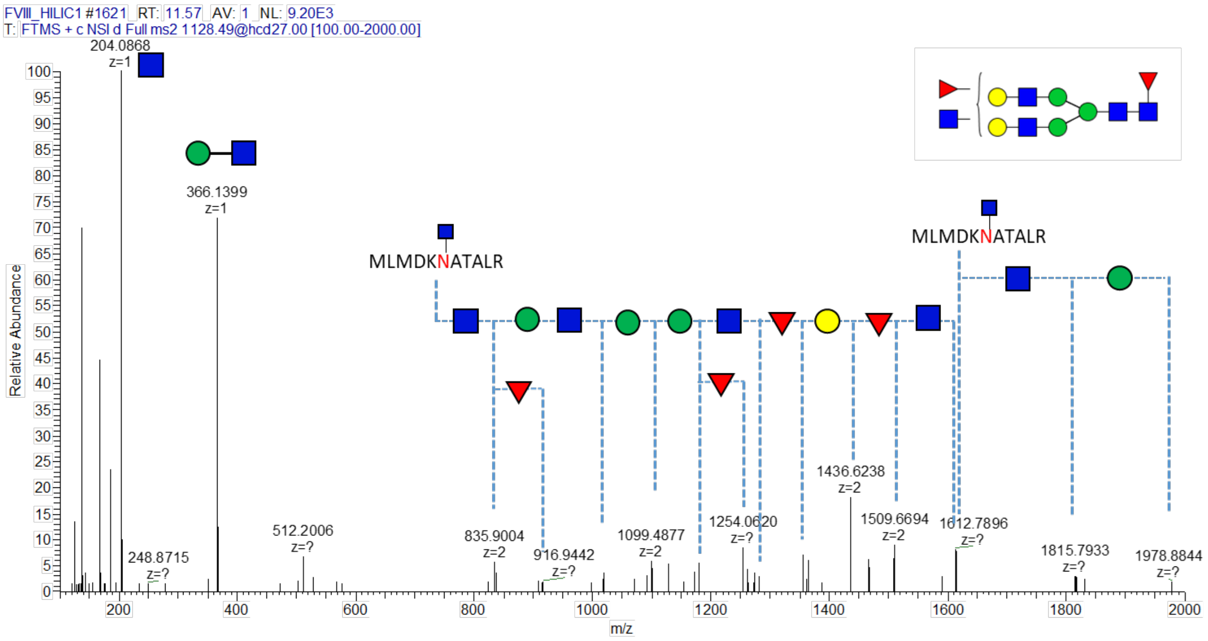

FVIII_HILIC1 #1621 RT: 11.57 AV: 1 NL: 9.20E3
T: FTMS + c NSI d Full ms2 1128.49@hcd27.00 [100.00-2000.00]
204.0868
z=1
366.1399
z=1
MLMDKNATALR
MLMDKNATALR
248.8715
z=?
512.2006
z=?
835.9004
z=2
916.9442
z=?
1099.4877
z=2
1254.0620
z=?
1436.6238
z=2
1509.6694
z=2
1612.7896
z=?
1815.7933
z=?
1978.8844
z=?
Relative Abundance
m/z

56-N

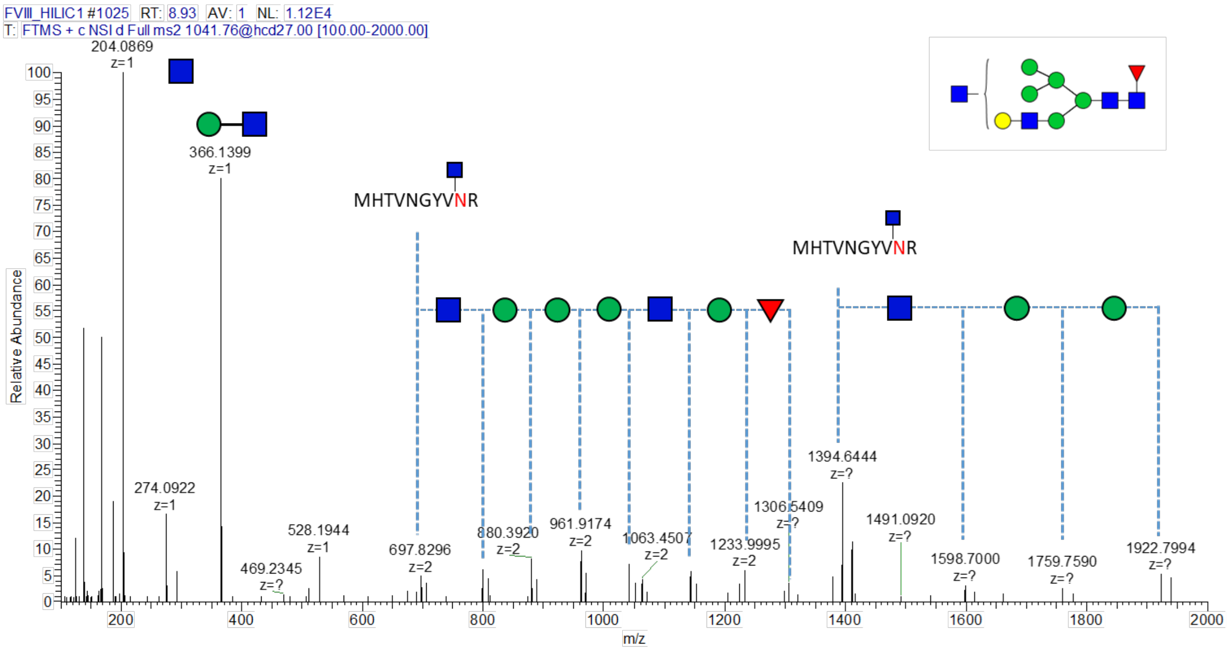

FVIII_HILIC1 #1025 RT: 8.93 AV: 1 NL: 1.12E4
T: FTMS + c NSI d Full ms2 1041.76@hcd27.00 [100.00-2000.00]
204.0869
z=1
366.1399
z=1
MHTVNGYVNR
MHTVNGYVNR
274.0922
z=1
469.2345
z=?
528.1944
z=1
697.8296
z=2
880.3920
z=2
961.9174
z=2
1063.4507
z=2
1233.9995
z=2
1306.5409
z=?
1394.6444
z=?
1491.0920
z=?
1598.7000
z=?
1759.7590
z=?
1922.7994
z=?
Relative Abundance
m/z

57-N

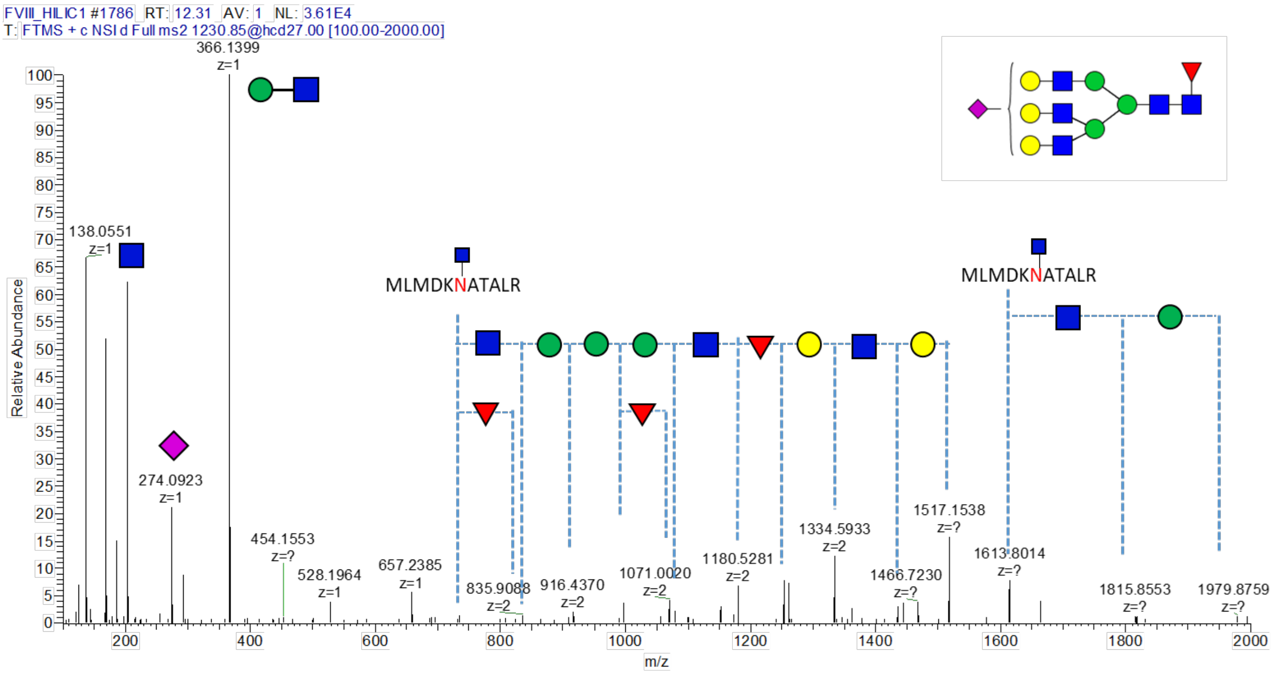
FVIII_HILIC1 #1786 RT: 12.31 AV: 1 NL: 3.61E4
T: FTMS + c NSI d Full ms2 1230.85@hcd27.00 [100.00-2000.00]
Relative Abundance
m/z
366.1399 z=1
138.0551 z=1
274.0923 z=1
454.1553 z=?
528.1964 z=1
657.2385 z=1
835.9088 z=2
916.4370 z=2
1071.0020 z=2
1180.5281 z=2
1334.5933 z=2
1466.7230 z=?
1517.1538 z=?
1613.8014 z=?
1815.8553 z=?
1979.8759 z=?
MLMDKNATALR
MLMDKNATALR

58-N

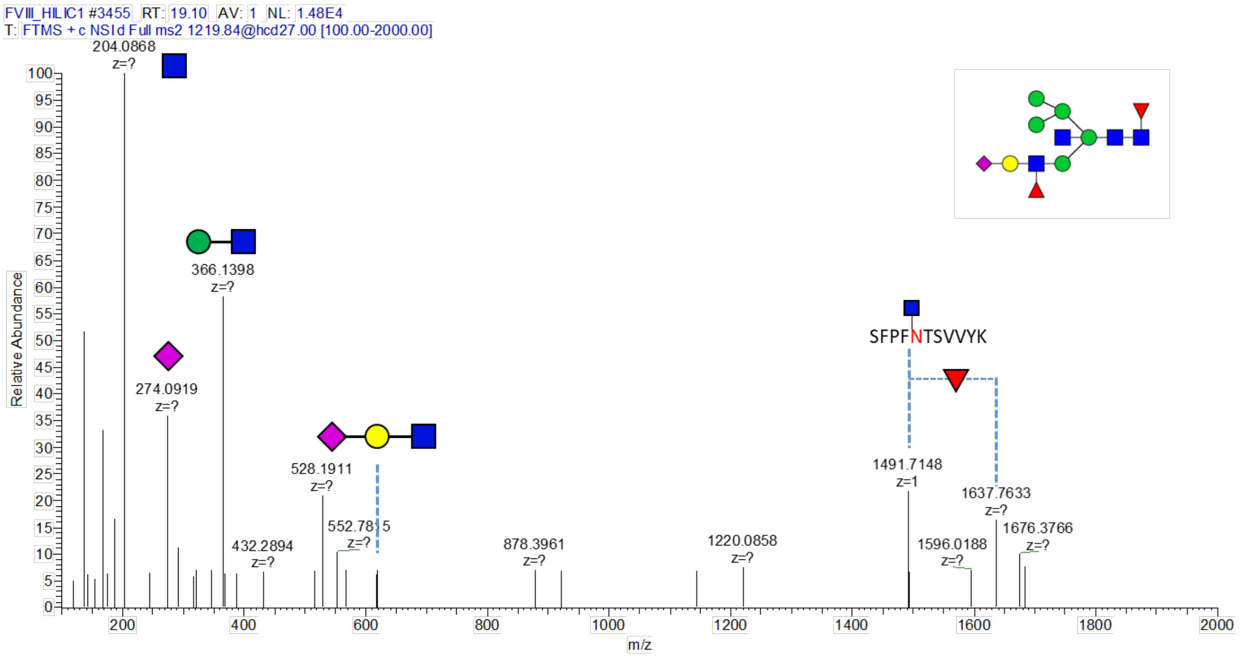
FVIII_HILIC1 #3455 RT: 19.10 AV: 1 NL: 1.48E4
T: FTMS + c NSI d Full ms2 1219.84@hcd27.00 [100.00-2000.00]
Relative Abundance
m/z
204.0868 z=?
366.1398 z=?
274.0919 z=?
528.1911 z=?
552.7815 z=?
432.2894 z=?
878.3961 z=?
1220.0858 z=?
1491.7148 z=1
1637.7633 z=?
1596.0188 z=?
1676.3766 z=?
SFPFNTSVVYK

59-N

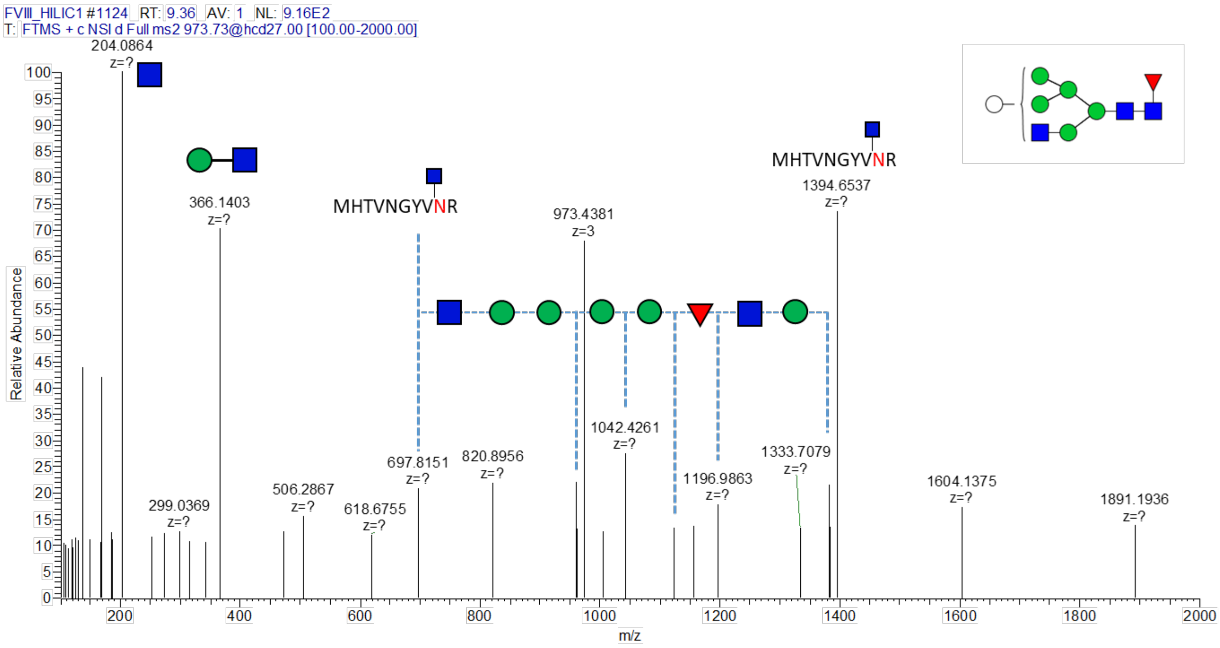
FVIII_HILIC1 #1124 RT: 9.36 AV: 1 NL: 9.16E2
T: FTMS + c NSI d Full ms2 973.73@hcd27.00 [100.00-2000.00]
204.0864 z=?
366.1403 z=?
MHTVNGYVNR
973.4381 z=3
MHTVNGYVNR
1394.6537 z=?
1042.4261 z=?
697.8151 z=?
820.8956 z=?
1333.7079 z=?
1196.9863 z=?
506.2867 z=?
618.6755 z=?
299.0369 z=?
1604.1375 z=?
1891.1936 z=?
Relative Abundance
m/z

60-N

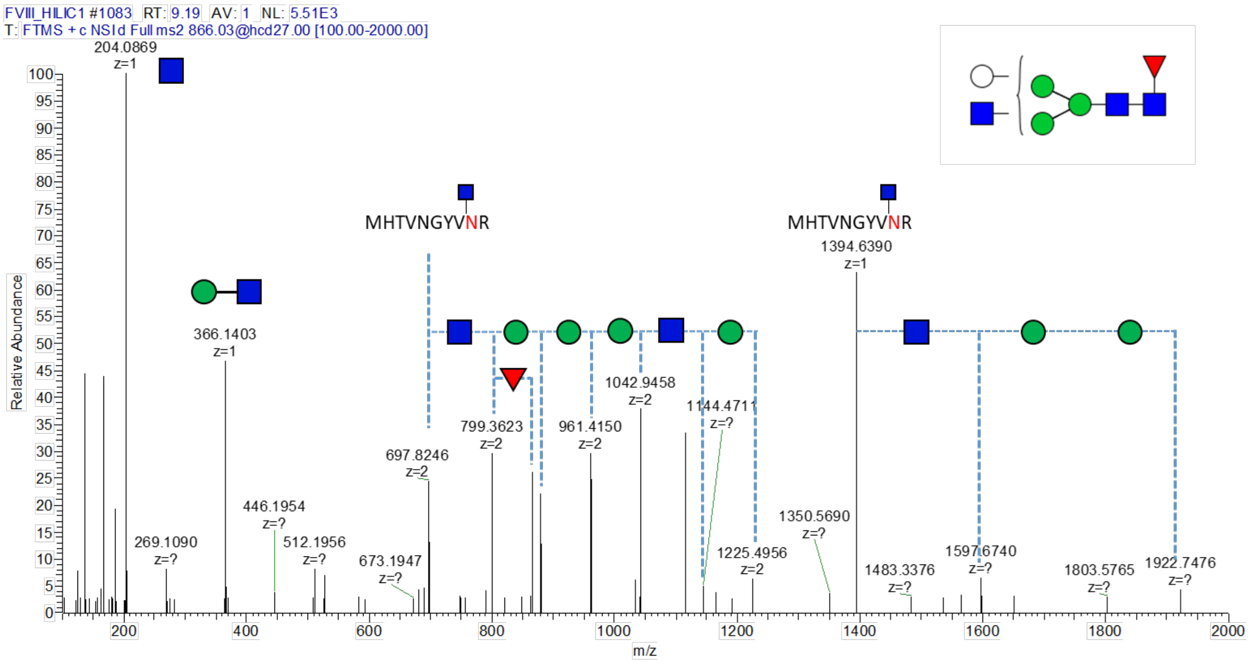
FVIII_HILIC1 #1083 RT: 9.19 AV: 1 NL: 5.51E3
T: FTMS + c NSI d Full ms2 866.03@hcd27.00 [100.00-2000.00]
204.0869 z=1
MHTVNGYVNR
MHTVNGYVNR
1394.6390 z=1
366.1403 z=1
1042.9458 z=2
1144.4711 z=?
799.3623 z=2
961.4150 z=2
697.8246 z=2
446.1954 z=?
1350.5690 z=?
269.1090 z=?
512.1956 z=?
673.1947 z=?
1225.4956 z=2
1597.6740 z=?
1483.3376 z=?
1803.5765 z=?
1922.7476 z=?
Relative Abundance
m/z

61-N

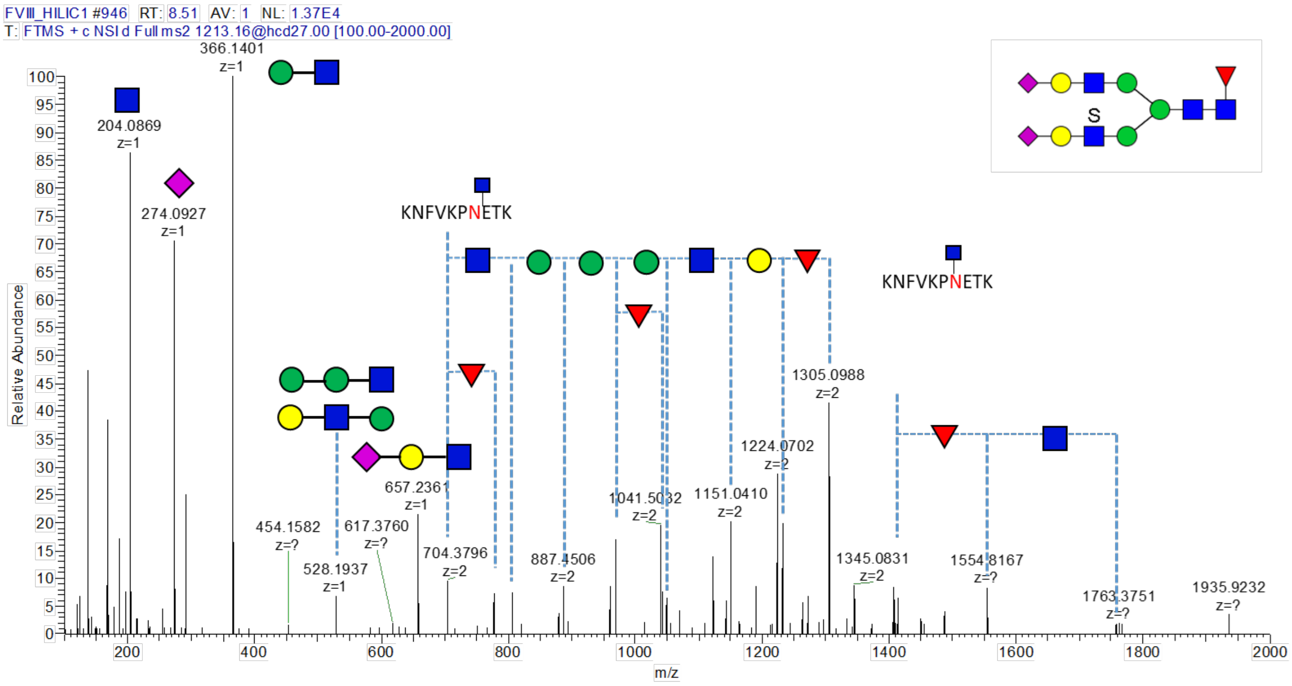
FVIII_HILIC1 #946 RT: 8.51 AV: 1 NL: 1.37E4
T: FTMS + c NSI d Full ms2 1213.16@hcd27.00 [100.00-2000.00]
Relative Abundance
m/z
KNFVKPNETK
S
366.1401 z=1
204.0869 z=1
274.0927 z=1
454.1582 z=?
528.1937 z=1
617.3760 z=?
657.2361 z=1
704.3796 z=2
887.4506 z=2
1041.5032 z=2
1151.0410 z=2
1224.0702 z=2
1305.0988 z=2
1345.0831 z=2
1554.8167 z=?
1763.3751 z=?
1935.9232 z=?

1-O

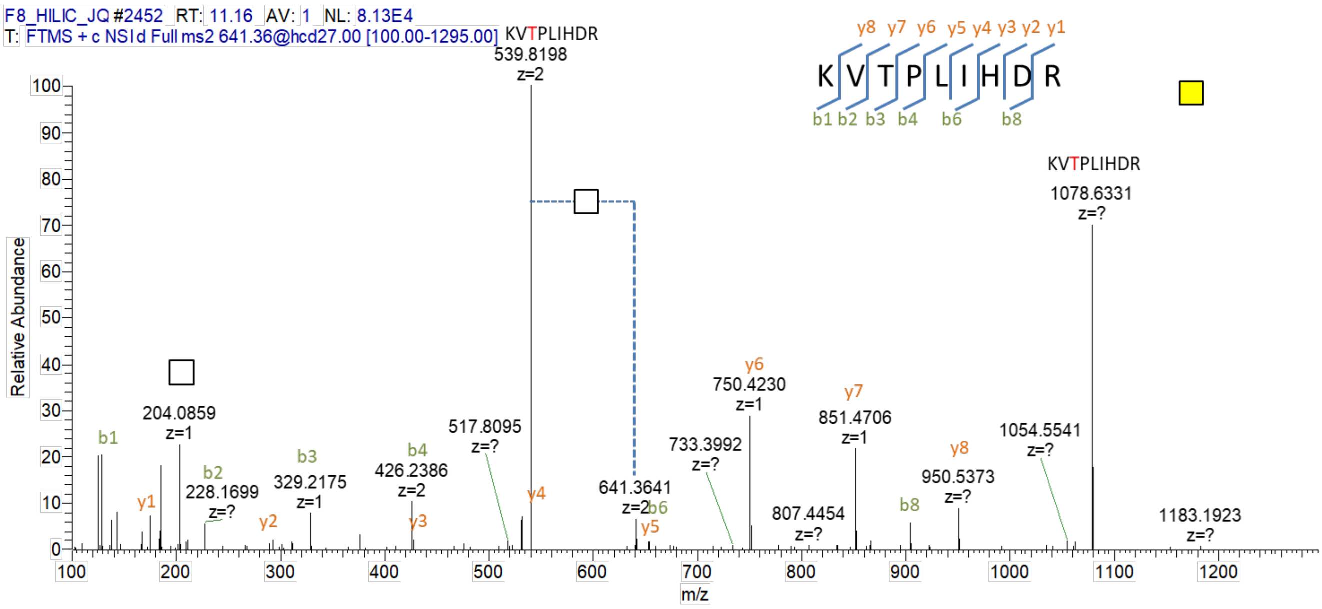
F8_HILIC_JQ #2452 RT: 11.16 AV: 1 NL: 8.13E4
T: FTMS + c NSI d Full ms2 641.36@hcd27.00 [100.00-1295.00]
KVTPLIHDR
y8 y7 y6 y5 y4 y3 y2 y1
K V T P L I H D R
b1 b2 b3 b4 b6 b8
Relative Abundance
m/z
539.8198 z=2
1078.6331 z=?
204.0859 z=1
228.1699 z=?
329.2175 z=1
426.2386 z=2
517.8095 z=?
641.3641 z=2
733.3992 z=?
750.4230 z=1
807.4454 z=?
851.4706 z=1
950.5373 z=?
1054.5541 z=?
1183.1923 z=?

2-O

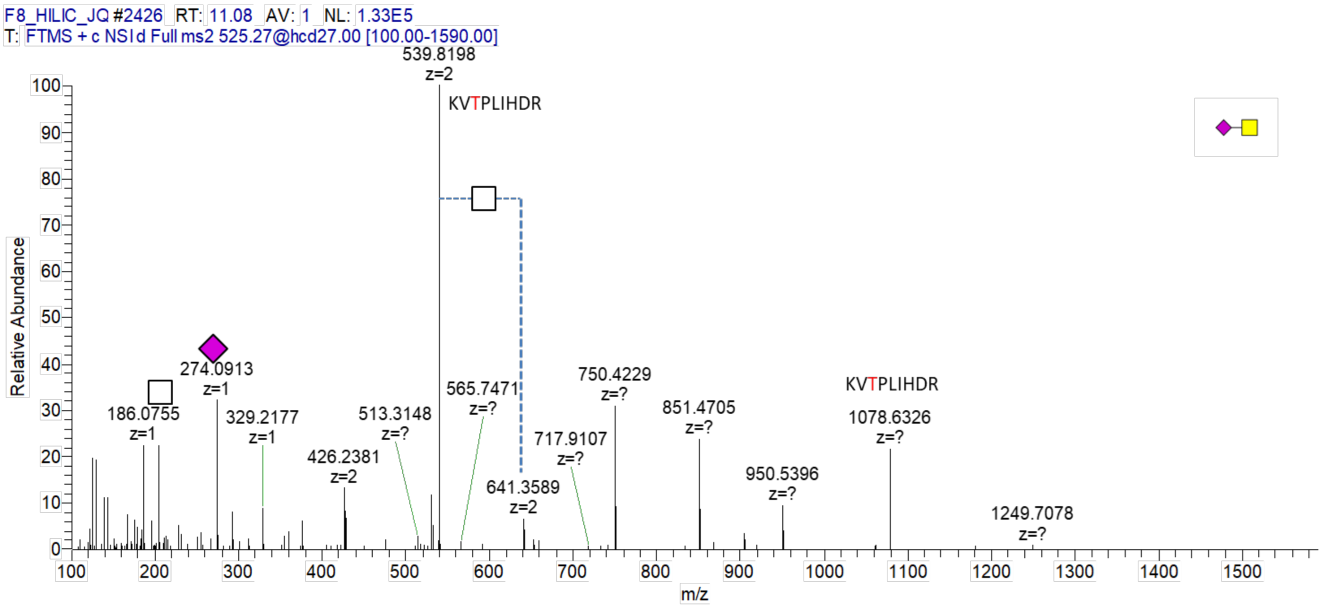

F8_HILIC_JQ #2426 RT: 11.08 AV: 1 NL: 1.33E5
T: FTMS + c NSI d Full ms2 525.27@hcd27.00 [100.00-1590.00]
539.8198 z=2
KVTPLIHDR
274.0913 z=1
186.0755 z=1
329.2177 z=1
513.3148 z=?
565.7471 z=?
426.2381 z=2
641.3589 z=2
717.9107 z=?
750.4229 z=?
851.4705 z=?
950.5396 z=?
KVTPLIHDR
1078.6326 z=?
1249.7078 z=?
Relative Abundance
m/z

3-O

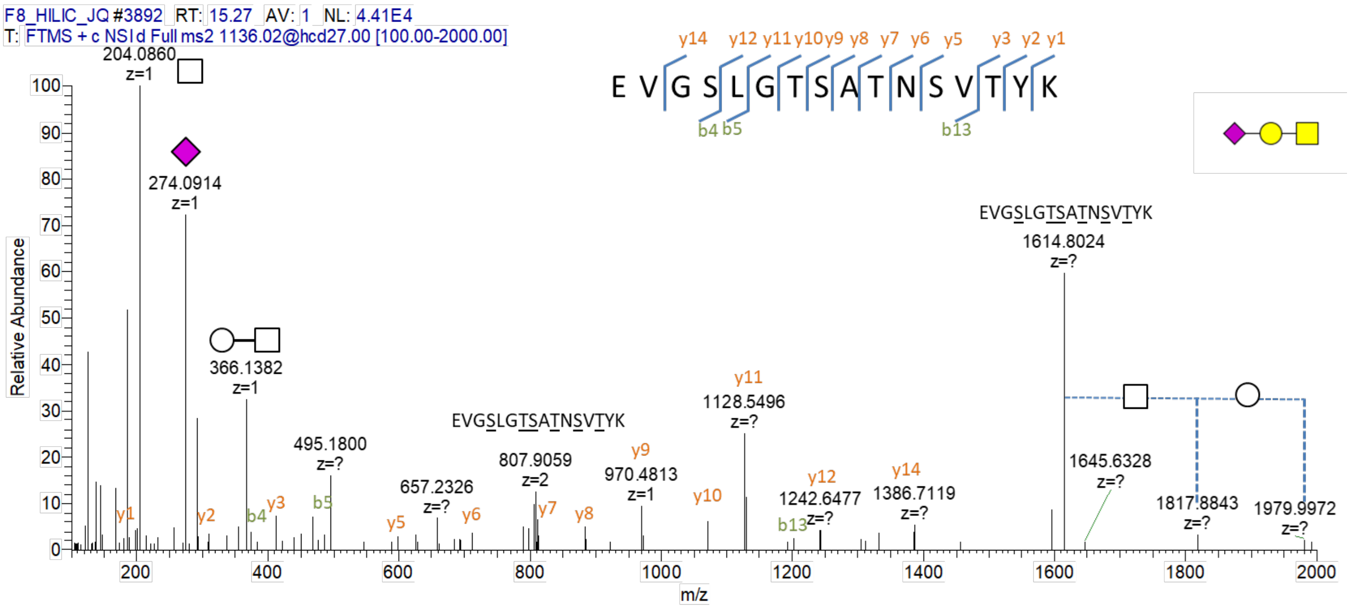

F8_HILIC_JQ #3892 RT: 15.27 AV: 1 NL: 4.41E4
T: FTMS + c NSI d Full ms2 1136.02@hcd27.00 [100.00-2000.00]
y14 y12 y11 y10 y9 y8 y7 y6 y5 y3 y2 y1
E V G S L G T S A T N S V T Y K
b4 b5 b13
204.0860 z=1
274.0914 z=1
366.1382 z=1
495.1800 z=?
657.2326 z=?
EVGSLGTSATNSVTYK
807.9059 z=2
970.4813 z=1
1128.5496 z=?
1242.6477 z=?
1386.7119 z=?
EVGSLGTSATNSVTYK
1614.8024 z=?
1645.6328 z=?
1817.8843 z=?
1979.9972 z=?
Relative Abundance
m/z

4-O

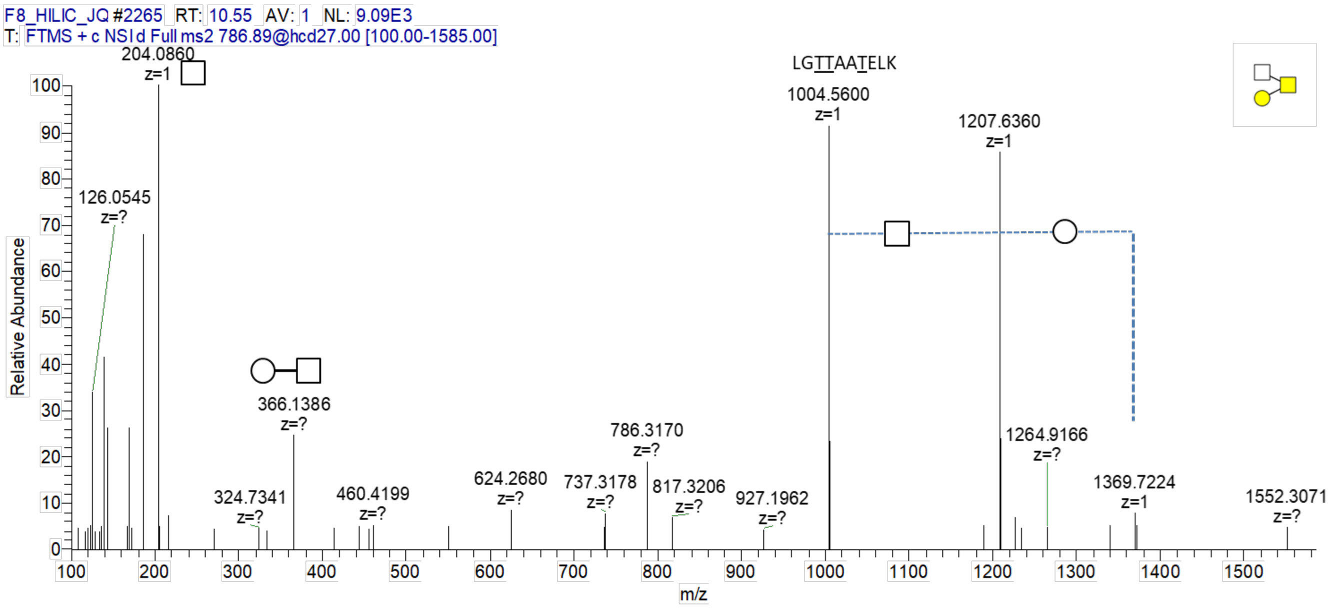
F8_HILIC_JQ #2265 RT: 10.55 AV: 1 NL: 9.09E3
T: FTMS + c NSI d Full ms2 786.89@hcd27.00 [100.00-1585.00]
LGTTAATELK
204.0860 z=1
126.0545 z=?
366.1386 z=?
324.7341 z=?
460.4199 z=?
624.2680 z=?
737.3178 z=?
786.3170 z=?
817.3206 z=?
927.1962 z=?
1004.5600 z=1
1207.6360 z=1
1264.9166 z=?
1369.7224 z=1
1552.3071 z=?
Relative Abundance
m/z

5-O

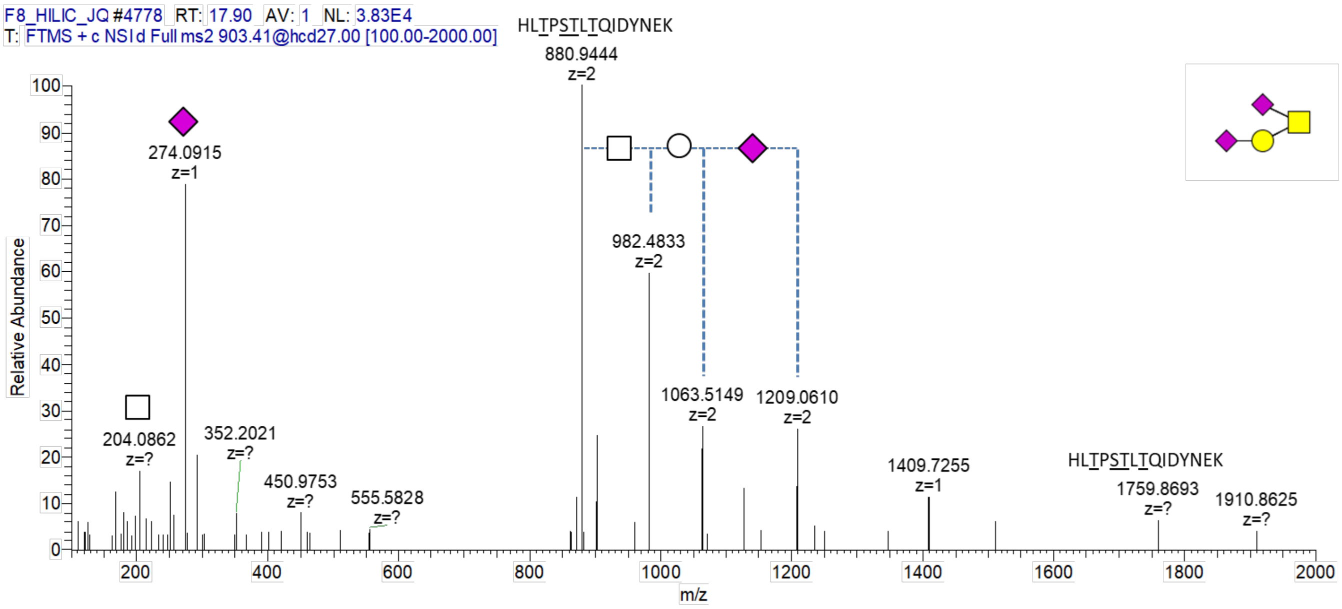
F8_HILIC_JQ #4778 RT: 17.90 AV: 1 NL: 3.83E4
T: FTMS + c NSI d Full ms2 903.41@hcd27.00 [100.00-2000.00]
HLTPSTLTQIDYNEK
880.9444 z=2
274.0915 z=1
204.0862 z=?
352.2021 z=?
450.9753 z=?
555.5828 z=?
982.4833 z=2
1063.5149 z=2
1209.0610 z=2
1409.7255 z=1
HLTPSTLTQIDYNEK
1759.8693 z=?
1910.8625 z=?
Relative Abundance
m/z

6-O

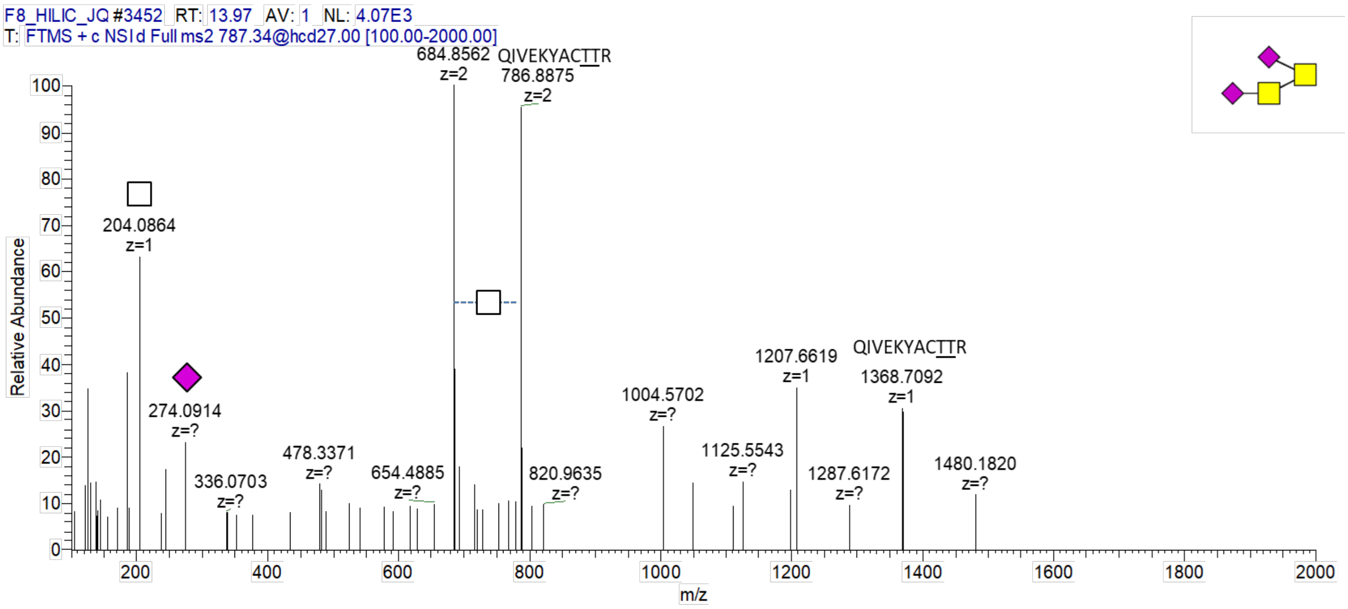

F8_HILIC_JQ #3452 RT: 13.97 AV: 1 NL: 4.07E3
T: FTMS + c NSI d Full ms2 787.34@hcd27.00 [100.00-2000.00]
684.8562 z=2
QIVEKYACTTR
786.8875 z=2
204.0864 z=1
274.0914 z=?
336.0703 z=?
478.3371 z=?
654.4885 z=?
820.9635 z=?
1004.5702 z=?
1125.5543 z=?
1207.6619 z=1
QIVEKYACTTR
1368.7092 z=1
1287.6172 z=?
1480.1820 z=?
Relative Abundance
m/z

7-O

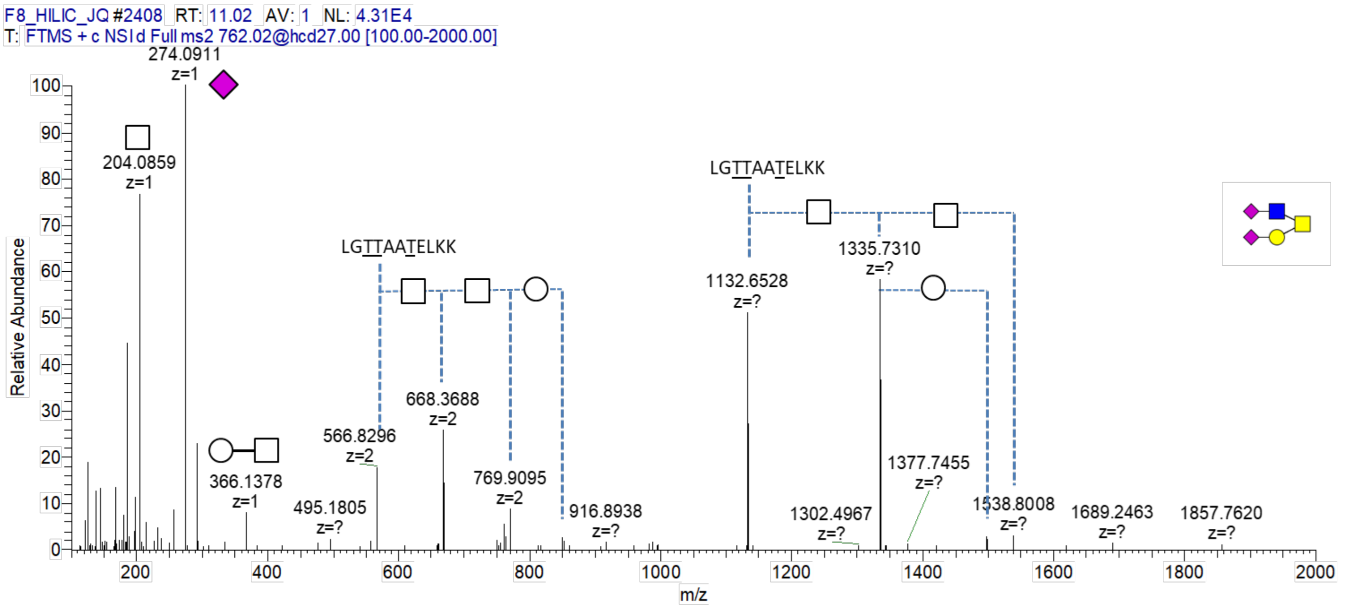

F8_HILIC_JQ #2408 RT: 11.02 AV: 1 NL: 4.31E4
T: FTMS + c NSI d Full ms2 762.02@hcd27.00 [100.00-2000.00]
274.0911 z=1
204.0859 z=1
366.1378 z=1
495.1805 z=?
LGTTAATELKK
566.8296 z=2
668.3688 z=2
769.9095 z=2
916.8938 z=?
LGTTAATELKK
1132.6528 z=?
1335.7310 z=?
1377.7455 z=?
1302.4967 z=?
1538.8008 z=?
1689.2463 z=?
1857.7620 z=?
Relative Abundance
m/z

8-O

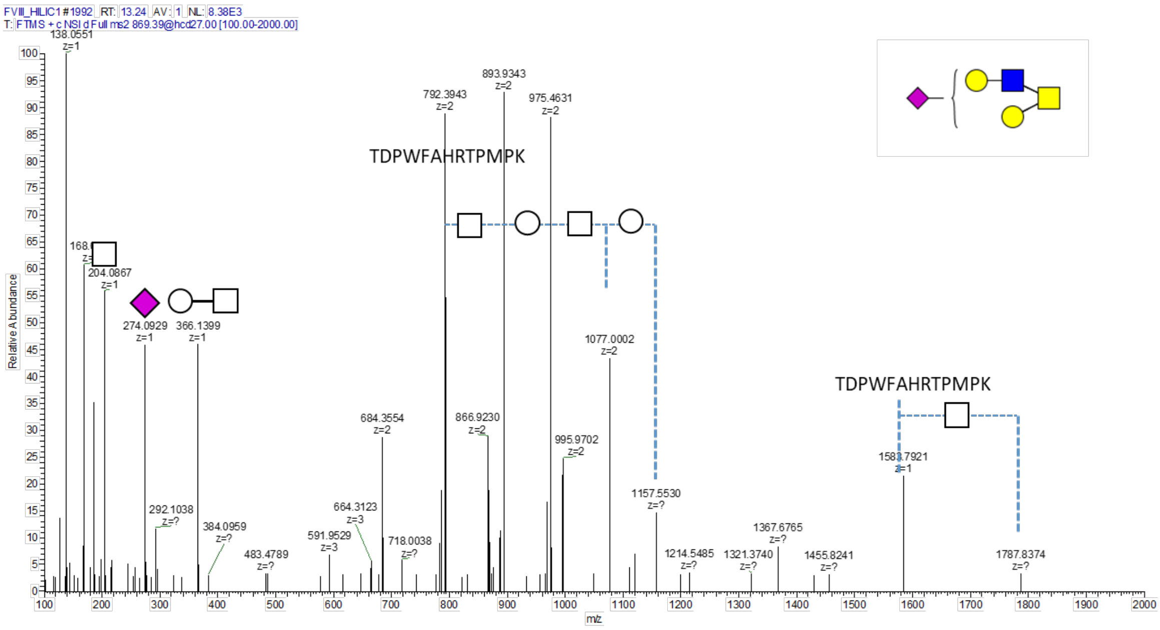
FVIII_HILIC1 #1992 RT: 13.24 AV: 1 NL: 8.38E3
T: FTMS + c NSI d Full ms2 869.39@hcd27.00 [100.00-2000.00]
TDPWFAHRTPMPK
TDPWFAHRTPMPK
Relative Abundance
m/z
138.0551 z=1
204.0867 z=1
274.0929 z=1
366.1399 z=1
292.1038 z=?
384.0959 z=?
483.4789 z=?
591.9529 z=3
664.3123 z=3
684.3554 z=2
718.0038 z=?
792.3943 z=2
866.9230 z=2
893.9343 z=2
975.4631 z=2
995.9702 z=2
1077.0002 z=2
1157.5530 z=?
1214.5485 z=?
1321.3740 z=?
1367.6765 z=?
1455.8241 z=?
1583.7921 z=1
1787.8374 z=?

9-O

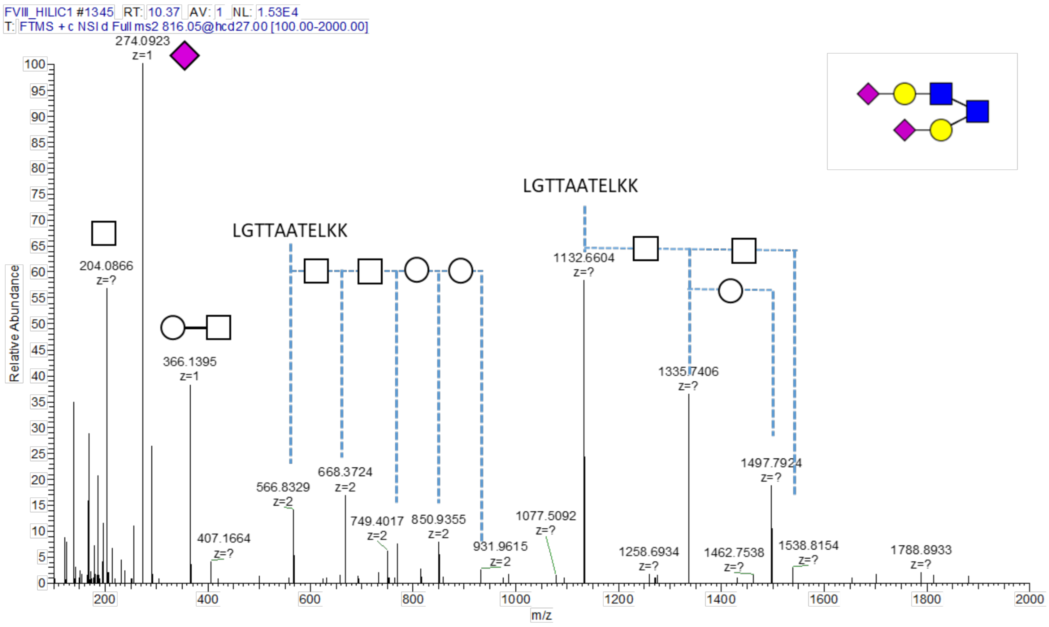
FVIII_HILIC1 #1345 RT: 10.37 AV: 1 NL: 1.53E4
T: FTMS + c NSI d Full ms2 816.05@hcd27.00 [100.00-2000.00]
LGTTAATELKK
LGTTAATELKK
Relative Abundance
m/z
274.0923 z=1
204.0866 z=?
366.1395 z=1
407.1664 z=?
566.8329 z=2
668.3724 z=2
749.4017 z=2
850.9355 z=2
931.9615 z=2
1077.5092 z=?
1132.6604 z=?
1258.6934 z=?
1335.7406 z=?
1462.7538 z=?
1497.7924 z=?
1538.8154 z=?
1788.8933 z=?

10-O

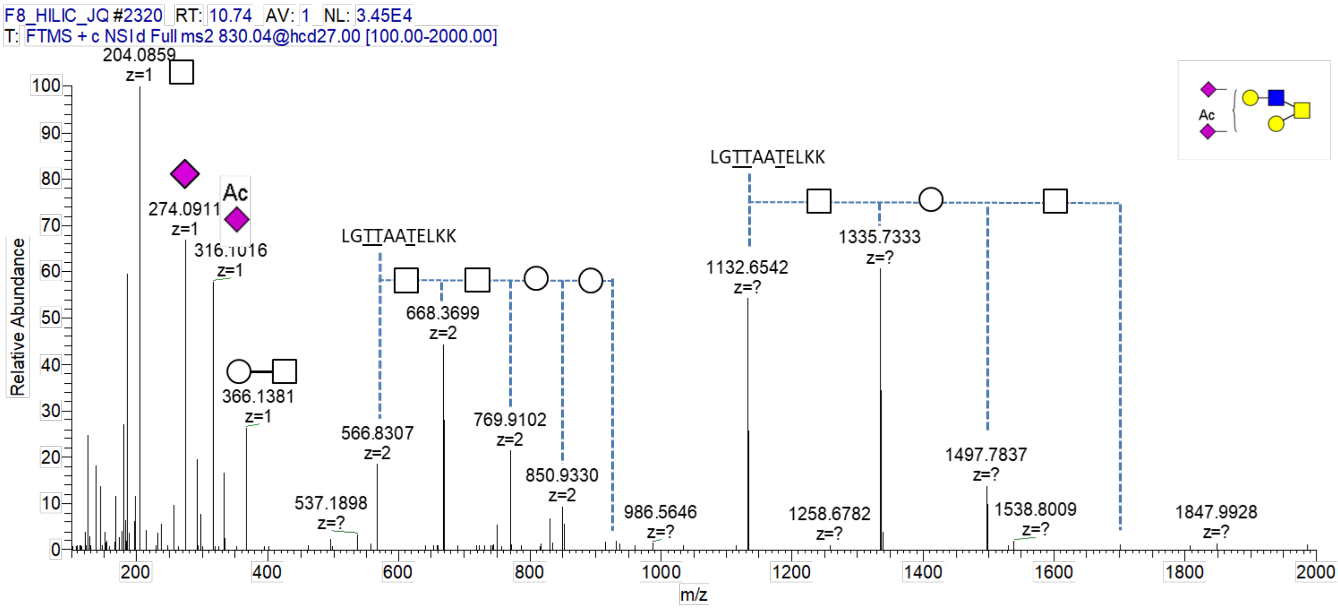

F8_HILIC_JQ #2320 RT: 10.74 AV: 1 NL: 3.45E4
T: FTMS + c NSI d Full ms2 830.04@hcd27.00 [100.00-2000.00]
204.0859
z=1
274.0911
z=1
Ac
316.1016
z=1
366.1381
z=1
LGTTAATELKK
566.8307
z=2
668.3699
z=2
769.9102
z=2
850.9330
z=2
537.1898
z=?
986.5646
z=?
LGTTAATELKK
1132.6542
z=?
1335.7333
z=?
1258.6782
z=?
1497.7837
z=?
1538.8009
z=?
1847.9928
z=?
Relative Abundance
m/z
Ac

11-O

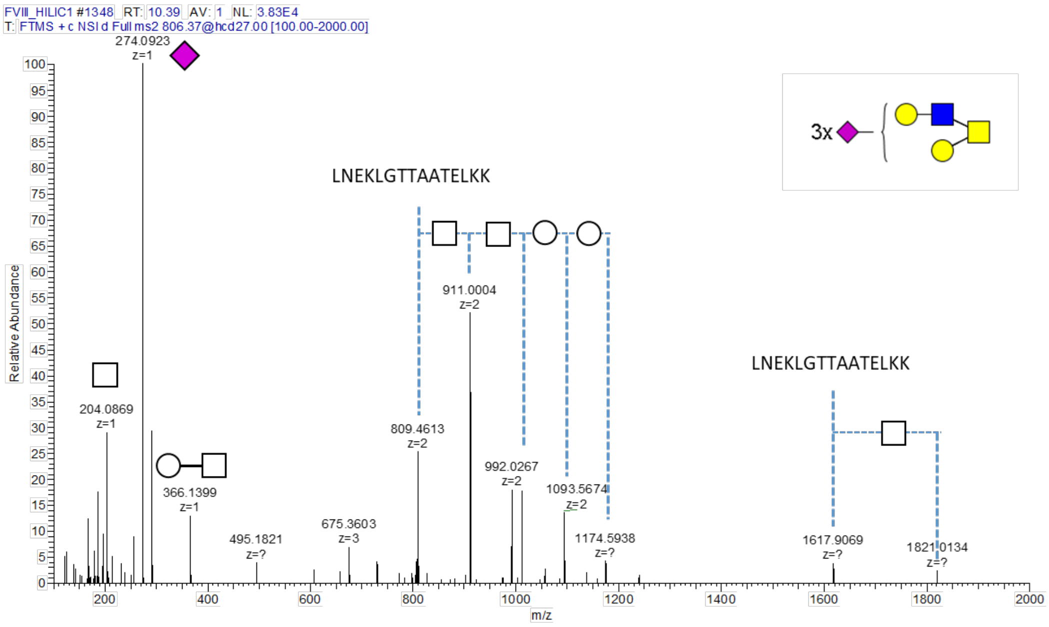

FVIII_HILIC1 #1348 RT: 10.39 AV: 1 NL: 3.83E4
T: FTMS + c NSI d Full ms2 806.37@hcd27.00 [100.00-2000.00]
274.0923
z=1
204.0869
z=1
366.1399
z=1
495.1821
z=?
675.3603
z=3
LNEKLGTTAATELKK
809.4613
z=2
911.0004
z=2
992.0267
z=2
1093.5674
z=2
1174.5938
z=?
LNEKLGTTAATELKK
1617.9069
z=?
1821.0134
z=?
Relative Abundance
m/z
3x

12-O

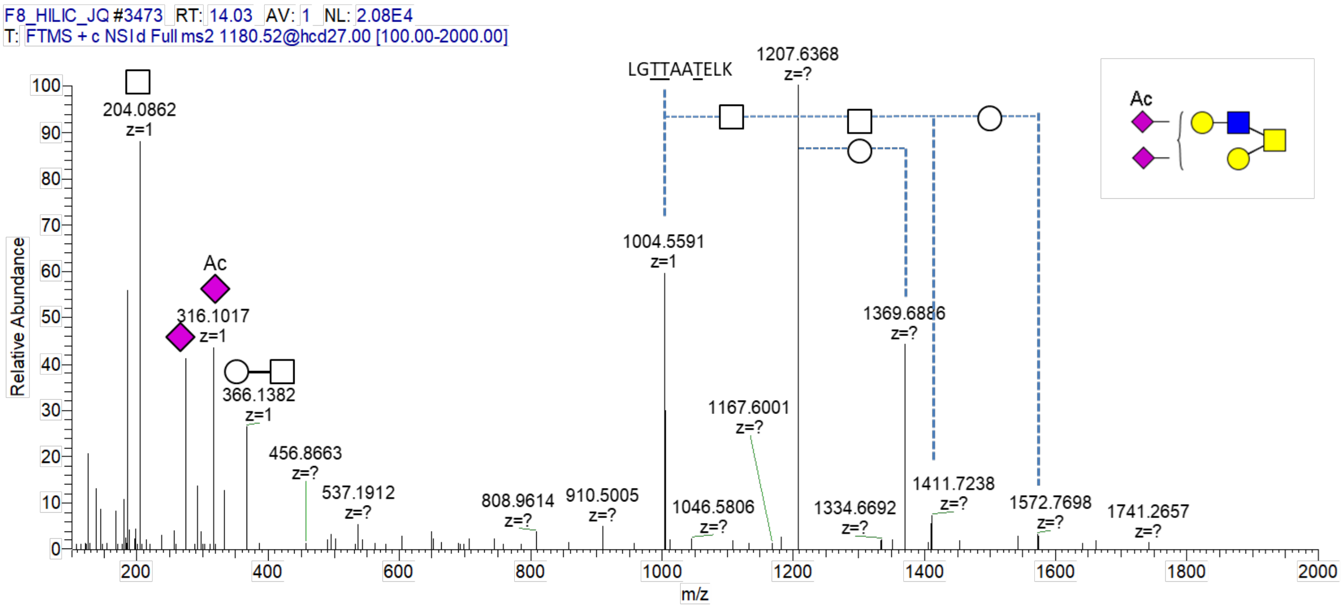
F8_HILIC_JQ #3473 RT: 14.03 AV: 1 NL: 2.08E4
T: FTMS + c NSI d Full ms2 1180.52@hcd27.00 [100.00-2000.00]
Relative Abundance
m/z
204.0862
z=1
Ac
316.1017
z=1
366.1382
z=1
456.8663
z=?
537.1912
z=?
808.9614
z=?
910.5005
z=?
LGTTAATELK
1004.5591
z=1
1046.5806
z=?
1167.6001
z=?
1207.6368
z=?
1334.6692
z=?
1369.6886
z=?
1411.7238
z=?
1572.7698
z=?
1741.2657
z=?
Ac

13-O

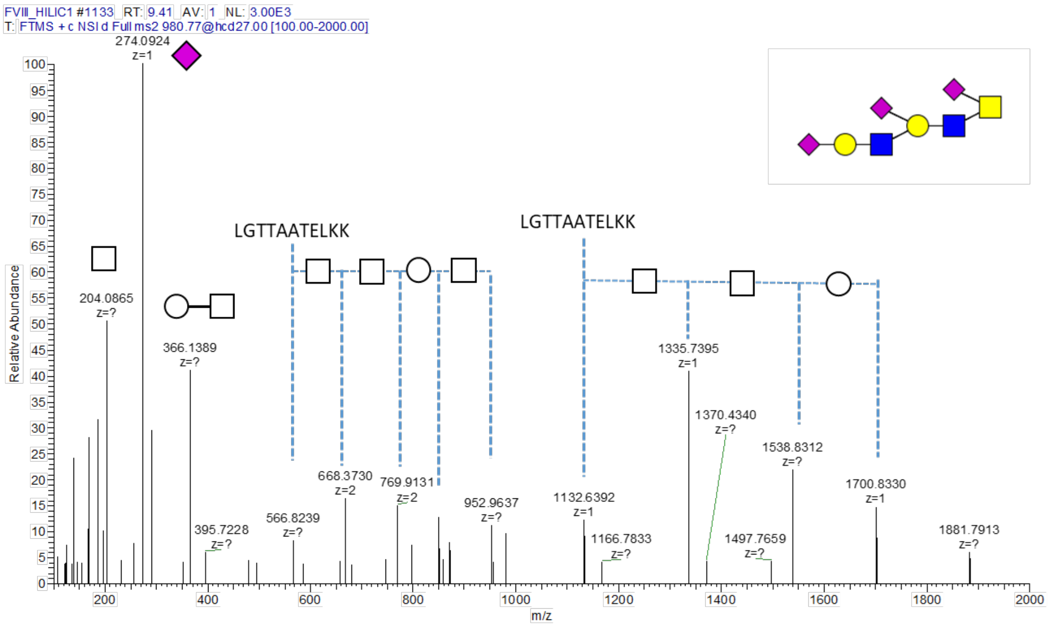
FVIII_HILIC1 #1133 RT: 9.41 AV: 1 NL: 3.00E3
T: FTMS + c NSI d Full ms2 980.77@hcd27.00 [100.00-2000.00]
Relative Abundance
m/z
274.0924
z=1
204.0865
z=?
366.1389
z=?
395.7228
z=?
566.8239
z=?
LGTTAATELKK
668.3730
z=2
769.9131
z=2
952.9637
z=?
LGTTAATELKK
1132.6392
z=1
1166.7833
z=?
1335.7395
z=1
1370.4340
z=?
1497.7659
z=?
1538.8312
z=?
1700.8330
z=1
1881.7913
z=?

14-O

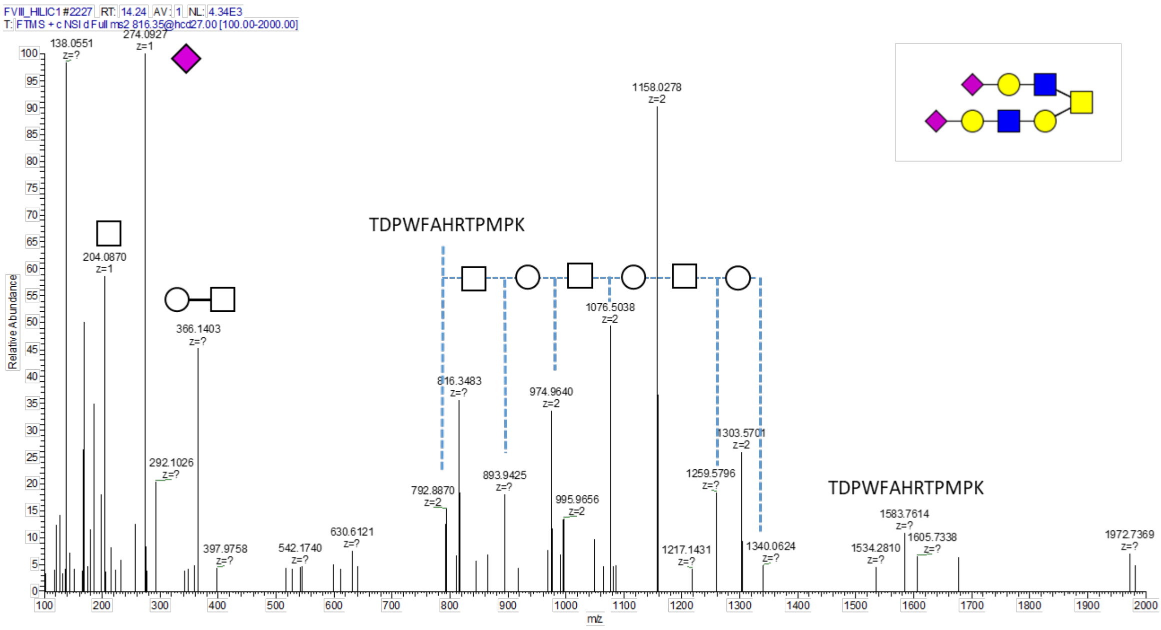

FVIII_HILIC1 #2227 RT: 14.24 AV: 1 NL: 4.34E3
T: FTMS + c NSI d Full ms2 816.35@hcd27.00 [100.00-2000.00]
TDPWFAHRTPMPK
TDPWFAHRTPMPK
138.0551 z=?
274.0927 z=1
204.0870 z=1
366.1403 z=?
292.1026 z=?
397.9758 z=?
542.1740 z=?
630.6121 z=?
792.8870 z=2
816.3483 z=?
893.9425 z=?
974.9640 z=2
995.9656 z=2
1076.5038 z=2
1158.0278 z=2
1217.1431 z=?
1259.5796 z=?
1303.5701 z=2
1340.0624 z=?
1534.2810 z=?
1583.7614 z=?
1605.7338 z=?
1972.7369 z=?
Relative Abundance
m/z

15-O

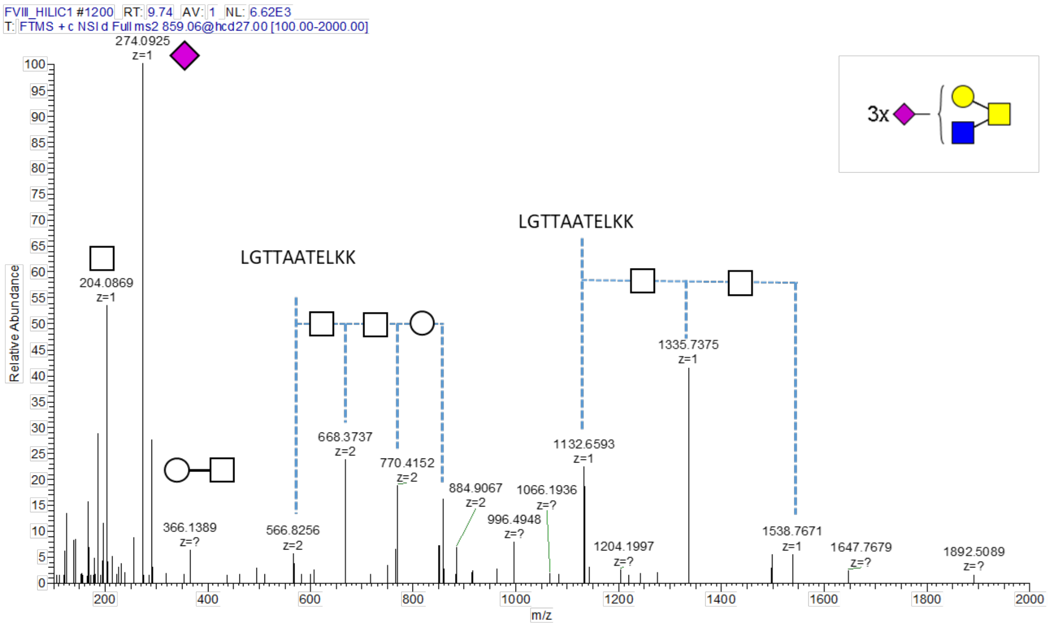

FVIII_HILIC1 #1200 RT: 9.74 AV: 1 NL: 6.62E3
T: FTMS + c NSI d Full ms2 859.06@hcd27.00 [100.00-2000.00]
3x
LGTTAATELKK
LGTTAATELKK
274.0925 z=1
204.0869 z=1
366.1389 z=?
566.8256 z=2
668.3737 z=2
770.4152 z=2
884.9067 z=2
996.4948 z=?
1066.1936 z=?
1132.6593 z=1
1204.1997 z=?
1335.7375 z=1
1538.7671 z=1
1647.7679 z=?
1892.5089 z=?
Relative Abundance
m/z

16-O

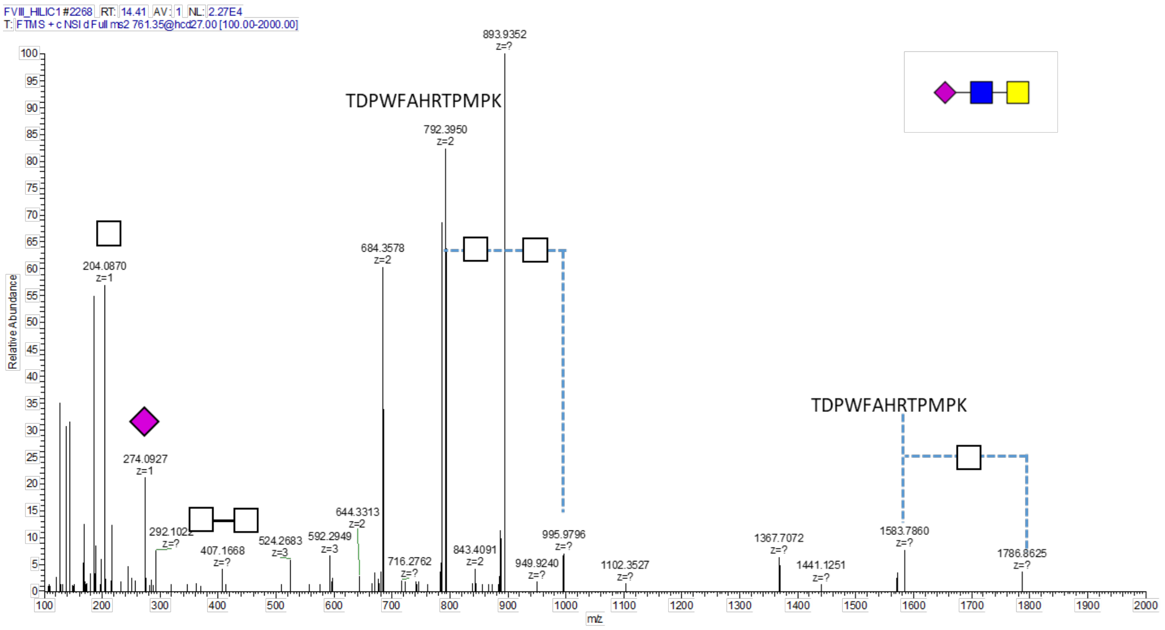
FVIII_HILIC1 #2268 RT: 14.41 AV: 1 NL: 2.27E4
T: FTMS + c NSI d Full ms2 761.35@hcd27.00 [100.00-2000.00]
TDPWFAHRTPMPK
893.9352 z=?
792.3950 z=2
684.3578 z=2
204.0870 z=1
274.0927 z=1
292.1022 z=?
407.1668 z=?
524.2683 z=3
592.2949 z=3
644.3313 z=2
716.2762 z=?
843.4091 z=2
949.9240 z=?
995.9796 z=?
1102.3527 z=?
1367.7072 z=?
1441.1251 z=?
1583.7860 z=?
1786.8625 z=?
Relative Abundance
m/z

17-O

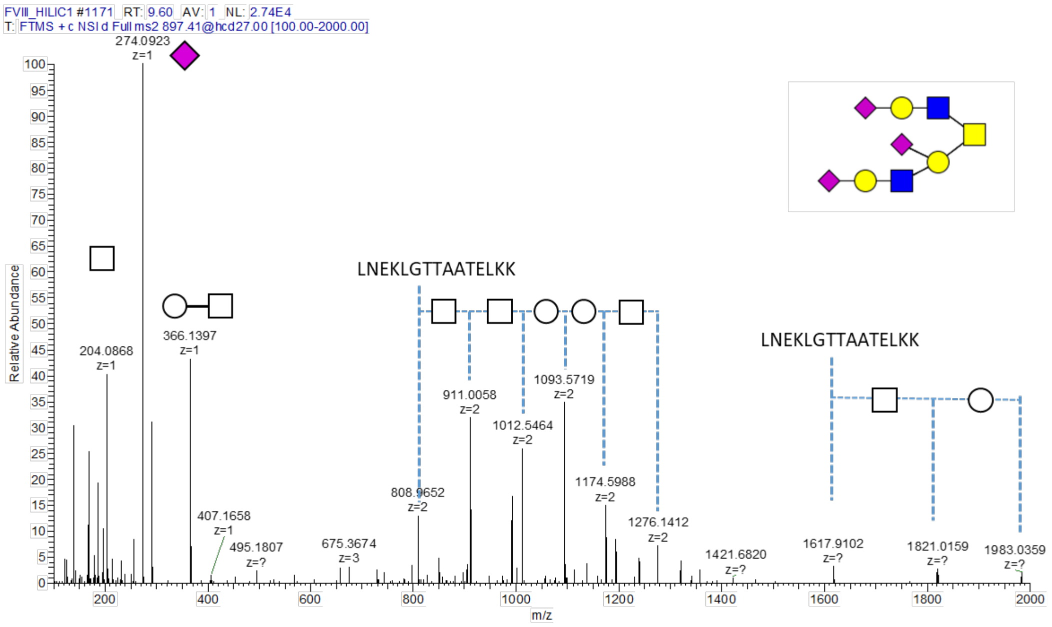
FVIII_HILIC1 #1171 RT: 9.60 AV: 1 NL: 2.74E4
T: FTMS + c NSI d Full ms2 897.41@hcd27.00 [100.00-2000.00]
274.0923 z=1
LNEKLGTTAATELKK
204.0868 z=1
366.1397 z=1
407.1658 z=1
495.1807 z=?
675.3674 z=3
808.9652 z=2
911.0058 z=2
1012.5464 z=2
1093.5719 z=2
1174.5988 z=2
1276.1412 z=2
1421.6820 z=?
1617.9102 z=?
1821.0159 z=?
1983.0359 z=?
Relative Abundance
m/z

18-O

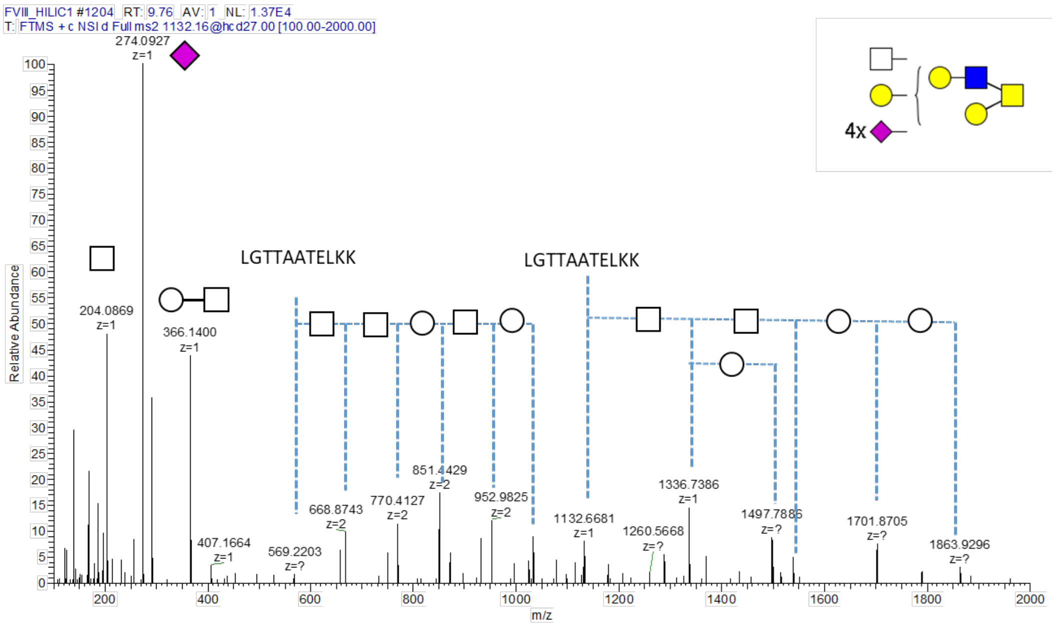

FVIII_HILIC1 #1204 RT: 9.76 AV: 1 NL: 1.37E4
T: FTMS + c NSI d Full ms2 1132.16@hcd27.00 [100.00-2000.00]
Relative Abundance
m/z
4x
LGTTAATELKK
LGTTAATELKK
274.0927
z=1
204.0869
z=1
366.1400
z=1
407.1664
z=1
569.2203
z=?
668.8743
z=2
770.4127
z=2
851.4429
z=2
952.9825
z=2
1132.6681
z=1
1260.5668
z=?
1336.7386
z=1
1497.7886
z=?
1701.8705
z=?
1863.9296
z=?

19-O

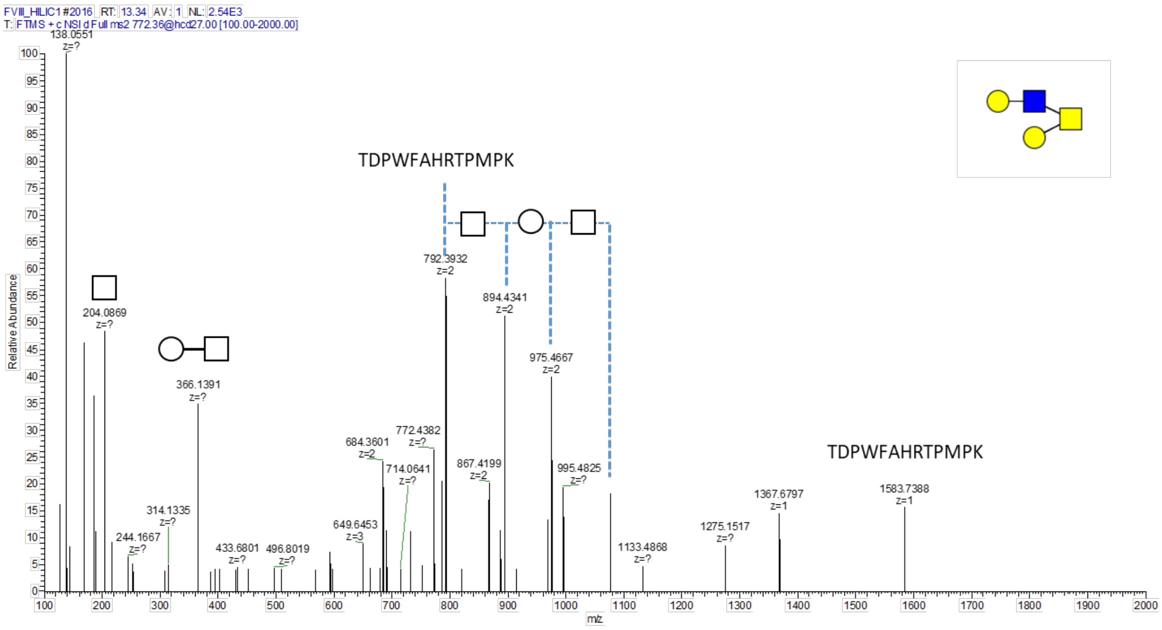

FVIII_HILIC1 #2016 RT: 13.34 AV: 1 NL: 2.54E3
T: FTMS + c NSI d Full ms2 772.36@hcd27.00 [100.00-2000.00]
Relative Abundance
m/z
TDPWFAHRTPMPK
TDPWFAHRTPMPK
138.0551
z=?
204.0869
z=?
366.1391
z=?
244.1667
z=?
314.1335
z=?
433.6801
z=?
496.8019
z=?
649.6453
z=3
684.3601
z=2
714.0641
z=?
772.4382
z=?
792.3932
z=2
867.4199
z=2
894.4341
z=2
975.4667
z=2
995.4825
z=?
1133.4868
z=?
1275.1517
z=?
1367.6797
z=1
1583.7388
z=1

20-O

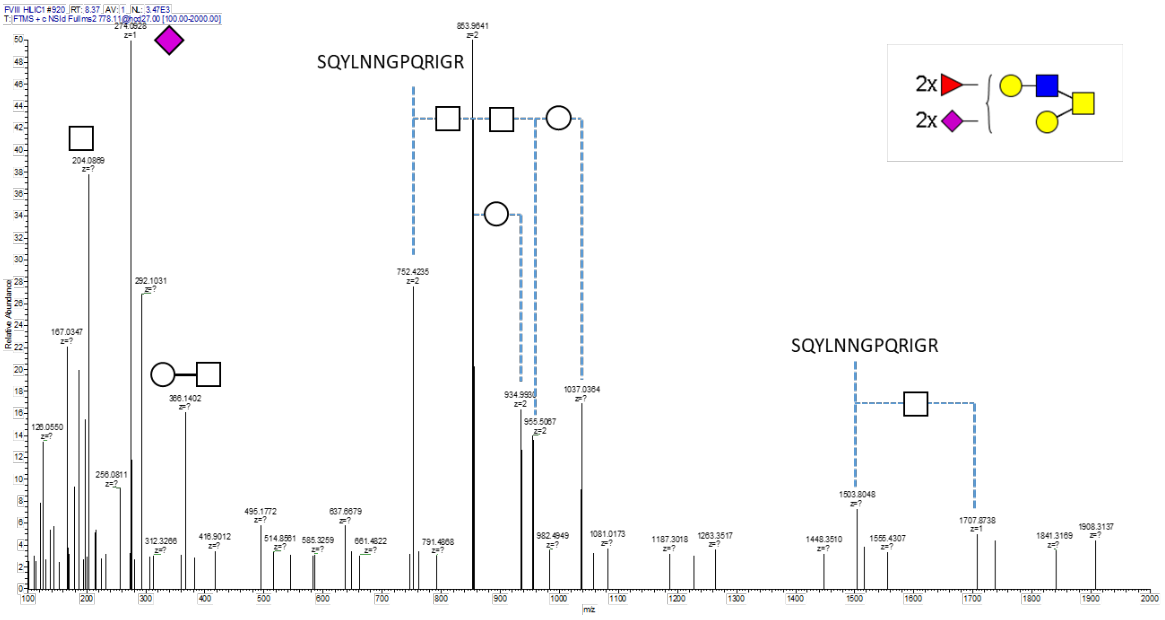
FVIII_HILIC1 #920 RT: 8.37 AV: 1 NL: 3.47E3
T: FTMS + c NSI d Full ms2 778.11@hcd27.00 [100.00-2000.00]
SQYLNNGPQRIGR
SQYLNNGPQRIGR
2x
2x
274.0928
853.9641
204.0869
292.1031
167.0347
366.1402
752.4235
934.9930
955.5067
1037.0364
1503.8048
1707.8738
1908.3137
1841.3169
Relative Abundance
m/z

21-O

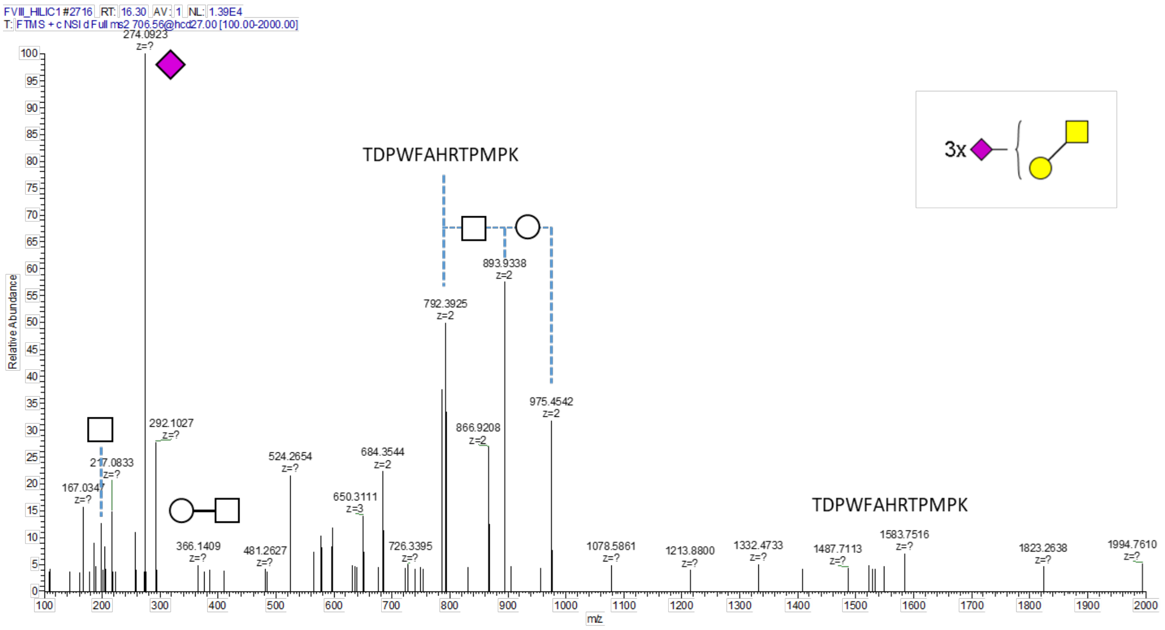
FVIII_HILIC1 #2716 RT: 16.30 AV: 1 NL: 1.39E4
T: FTMS + c NSI d Full ms2 706.56@hcd27.00 [100.00-2000.00]
TDPWFAHRTPMPK
TDPWFAHRTPMPK
3x
274.0923
893.9338
792.3925
975.4542
866.9208
292.1027
217.0833
167.0347
524.2654
684.3544
650.3111
366.1409
481.2627
726.3395
1078.5861
1213.8800
1332.4733
1487.7113
1583.7516
1823.2638
1994.7610
Relative Abundance
m/z

22-O

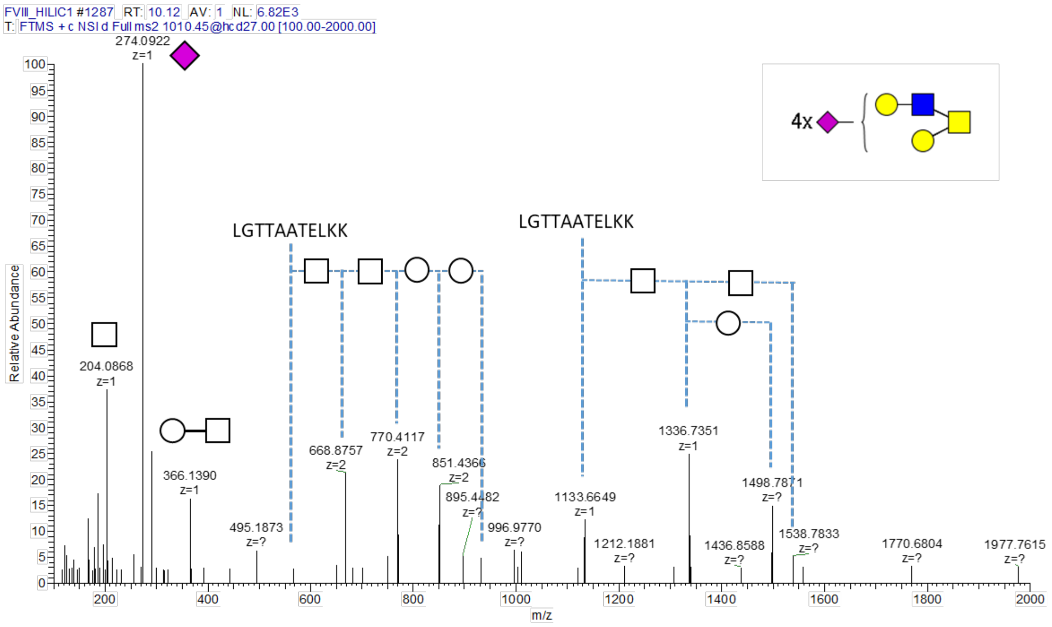

FVIII_HILIC1 #1287 RT: 10.12 AV: 1 NL: 6.82E3
T: FTMS + c NSI d Full ms2 1010.45@hcd27.00 [100.00-2000.00]
Relative Abundance
m/z
4x
LGTTAATELKK
LGTTAATELKK
274.0922 z=1
204.0868 z=1
366.1390 z=1
495.1873 z=?
668.8757 z=2
770.4117 z=2
851.4366 z=2
895.4482 z=?
996.9770 z=?
1133.6649 z=1
1212.1881 z=?
1336.7351 z=1
1436.8588 z=?
1498.7871 z=?
1538.7833 z=?
1770.6804 z=?
1977.7615 z=?

23-O

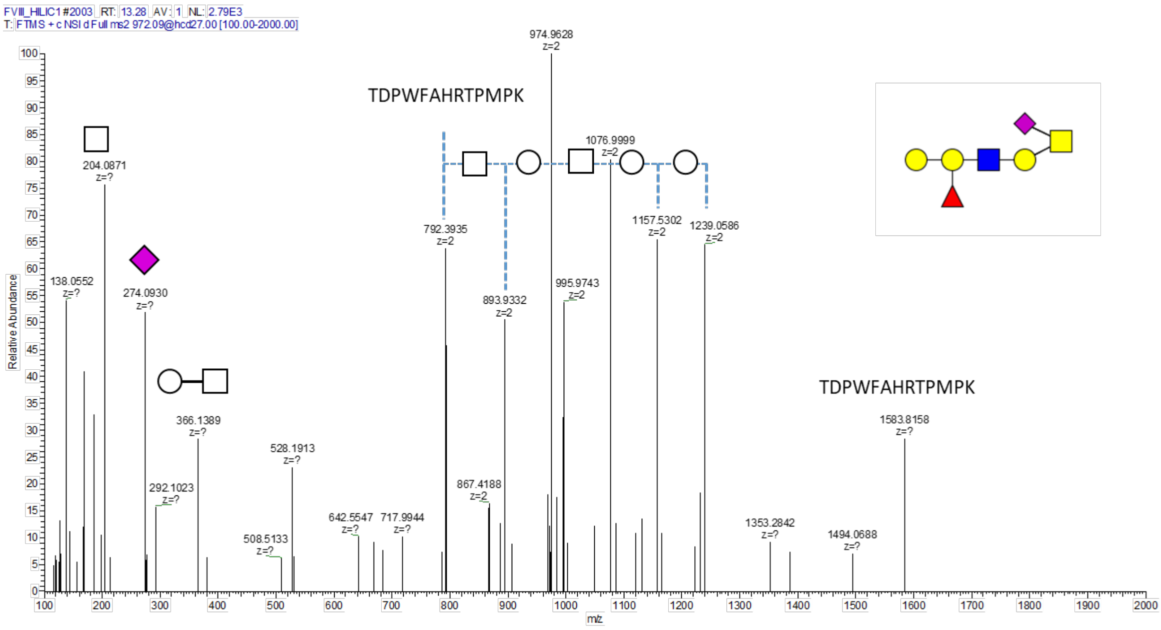

FVIII_HILIC1 #2003 RT: 13.28 AV: 1 NL: 2.79E3
T: FTMS + c NSI d Full ms2 972.09@hcd27.00 [100.00-2000.00]
Relative Abundance
m/z
TDPWFAHRTPMPK
TDPWFAHRTPMPK
974.9628 z=2
204.0871 z=?
138.0552 z=?
274.0930 z=?
366.1389 z=?
292.1023 z=?
528.1913 z=?
508.5133 z=?
642.5547 z=?
717.9944 z=?
792.3935 z=2
867.4188 z=2
893.9332 z=2
995.9743 z=2
1076.9999 z=2
1157.5302 z=2
1239.0586 z=2
1353.2842 z=?
1494.0688 z=?
1583.8158 z=?

24-O

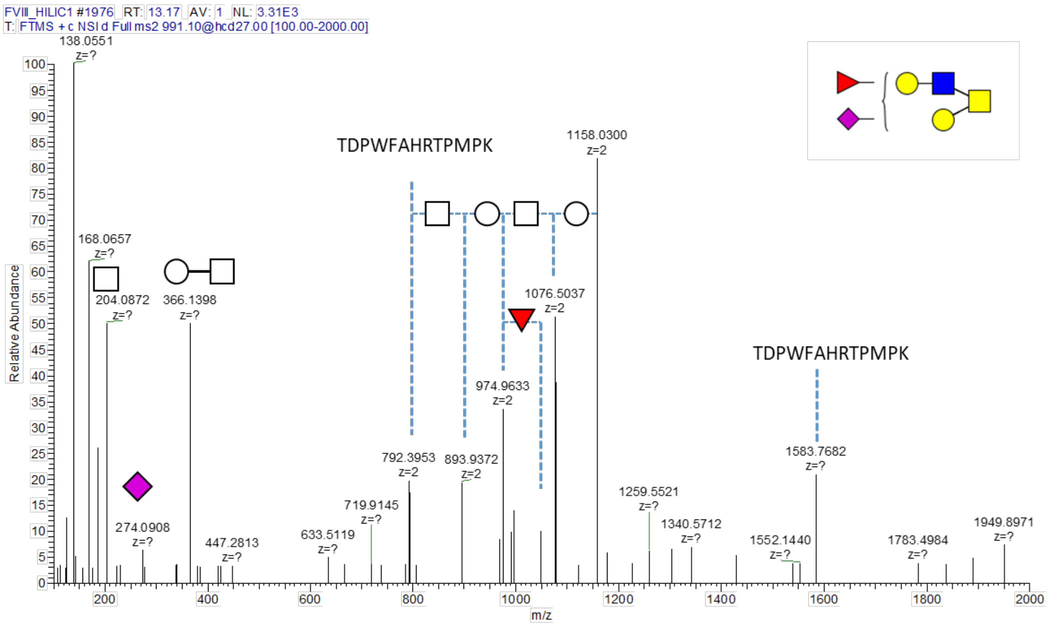

FVIII_HILIC1 #1976 RT: 13.17 AV: 1 NL: 3.31E3
T: FTMS + c NSI d Full ms2 991.10@hcd27.00 [100.00-2000.00]
Relative Abundance
m/z
TDPWFAHRTPMPK
138.0551 z=?
168.0657 z=?
204.0872 z=?
366.1398 z=?
274.0908 z=?
447.2813 z=?
633.5119 z=?
719.9145 z=?
792.3953 z=2
893.9372 z=2
974.9633 z=2
1076.5037 z=2
1158.0300 z=2
1259.5521 z=?
1340.5712 z=?
1552.1440 z=?
1583.7682 z=?
1783.4984 z=?
1949.8971 z=?

25-O

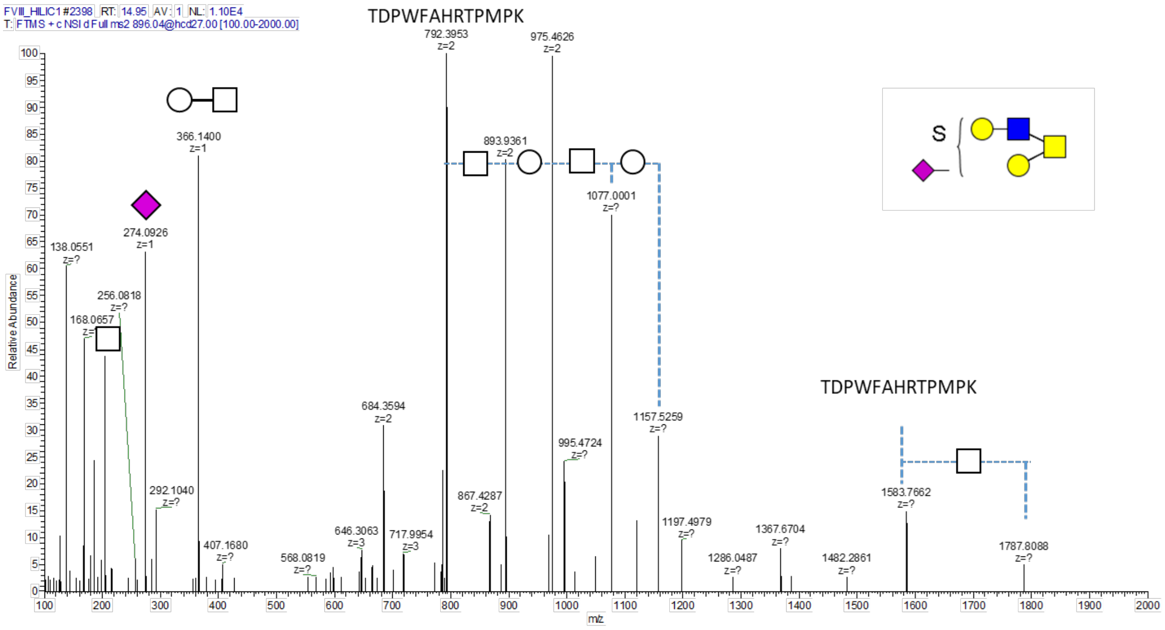

FVIII_HILIC1 #2398 RT: 14.95 AV: 1 NL: 1.10E4
T: FTMS + c NSI d Full ms2 896.04@hcd27.00 [100.00-2000.00]
Relative Abundance
m/z
TDPWFAHRTPMPK
S
792.3953 z=2
975.4626 z=2
366.1400 z=1
893.9361 z=2
1077.0001 z=?
274.0926 z=1
138.0551 z=?
256.0818 z=?
168.0657
1157.5259 z=?
684.3594 z=2
995.4724 z=?
292.1040 z=?
867.4287 z=2
1583.7662 z=?
1197.4979 z=?
646.3063 z=3
717.9954 z=3
1367.6704 z=?
407.1680 z=?
568.0819 z=?
1286.0487 z=?
1482.2861 z=?
1787.8088 z=?

26-O

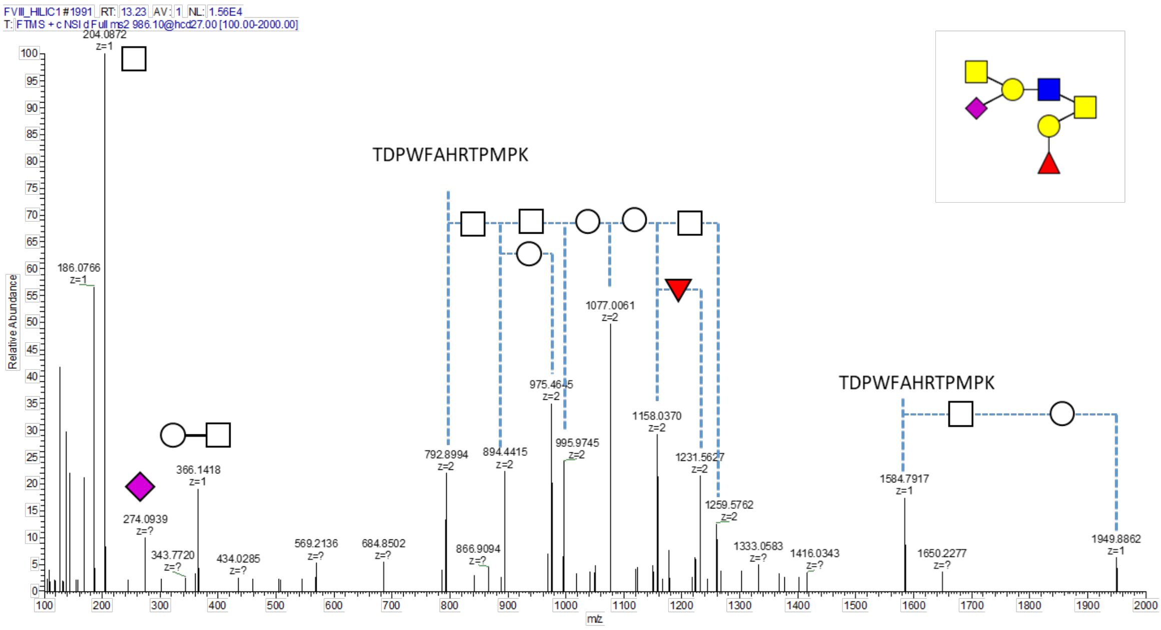

FVIII_HILIC1 #1991 RT: 13.23 AV: 1 NL: 1.56E4
T: FTMS + c NSI d Full ms2 986.10@hcd27.00 [100.00-2000.00]
TDPWFAHRTPMPK
TDPWFAHRTPMPK
204.0872 z=1
186.0766 z=1
274.0939 z=?
343.7720 z=?
366.1418 z=1
434.0285 z=?
569.2136 z=?
684.8502 z=?
792.8994 z=2
866.9094 z=2
894.4415 z=2
975.4645 z=2
995.9745 z=2
1077.0061 z=2
1158.0370 z=2
1231.5627 z=2
1259.5762 z=2
1333.0583 z=?
1416.0343 z=?
1584.7917 z=1
1650.2277 z=?
1949.8862 z=1
Relative Abundance
m/z

27-O

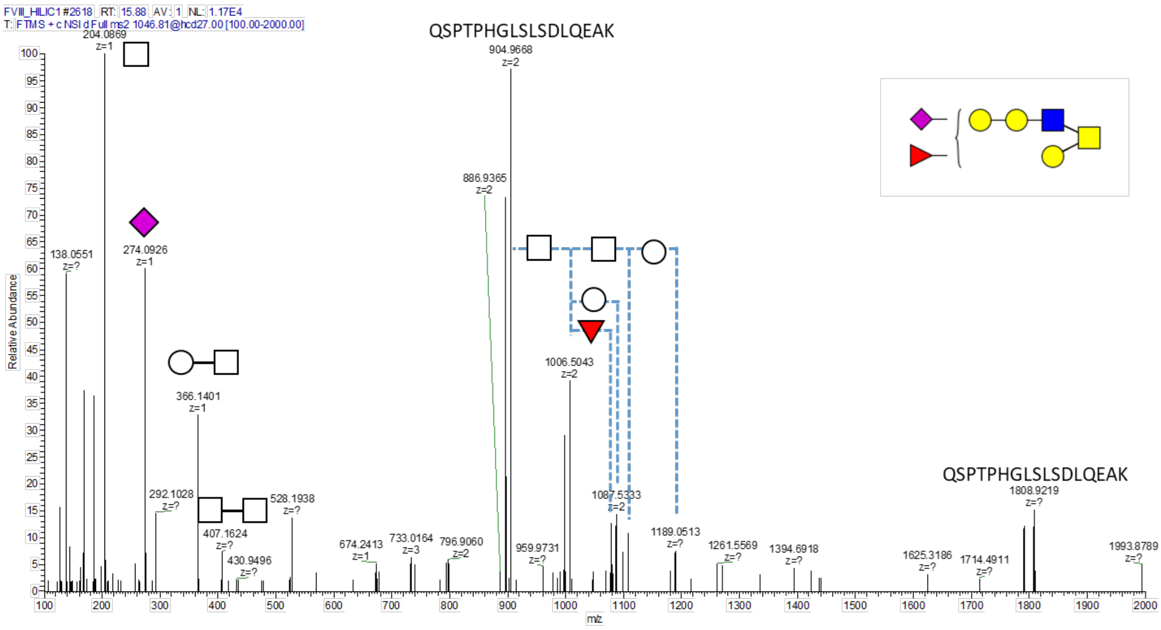

FVIII_HILIC1 #2618 RT: 15.88 AV: 1 NL: 1.17E4
T: FTMS + c NSI d Full ms2 1046.81@hcd27.00 [100.00-2000.00]
QSPTPHGLSLSDLQEAK
QSPTPHGLSLSDLQEAK
204.0869 z=1
138.0551 z=?
274.0926 z=1
292.1028 z=?
366.1401 z=1
407.1624 z=?
430.9496 z=?
528.1938 z=?
674.2413 z=1
733.0164 z=3
796.9060 z=2
886.9365 z=2
904.9668 z=2
959.9731 z=?
1006.5043 z=2
1087.5333 z=2
1189.0513 z=?
1261.5569 z=?
1394.6918 z=?
1625.3186 z=?
1714.4911 z=?
1808.9219 z=?
1993.8789 z=?
Relative Abundance
m/z

28-O

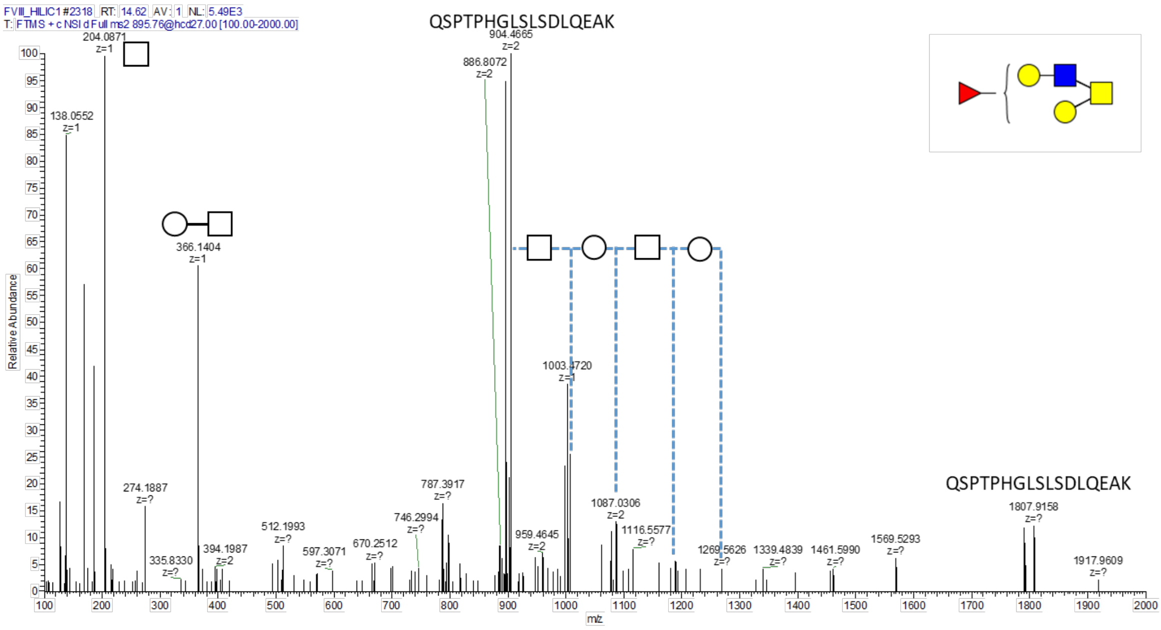

FVIII_HILIC1 #2318 RT: 14.62 AV: 1 NL: 5.49E3
T: FTMS + c NSI d Full ms2 895.76@hcd27.00 [100.00-2000.00]
QSPTPHGLSLSDLQEAK
QSPTPHGLSLSDLQEAK
Relative Abundance
m/z

29-O

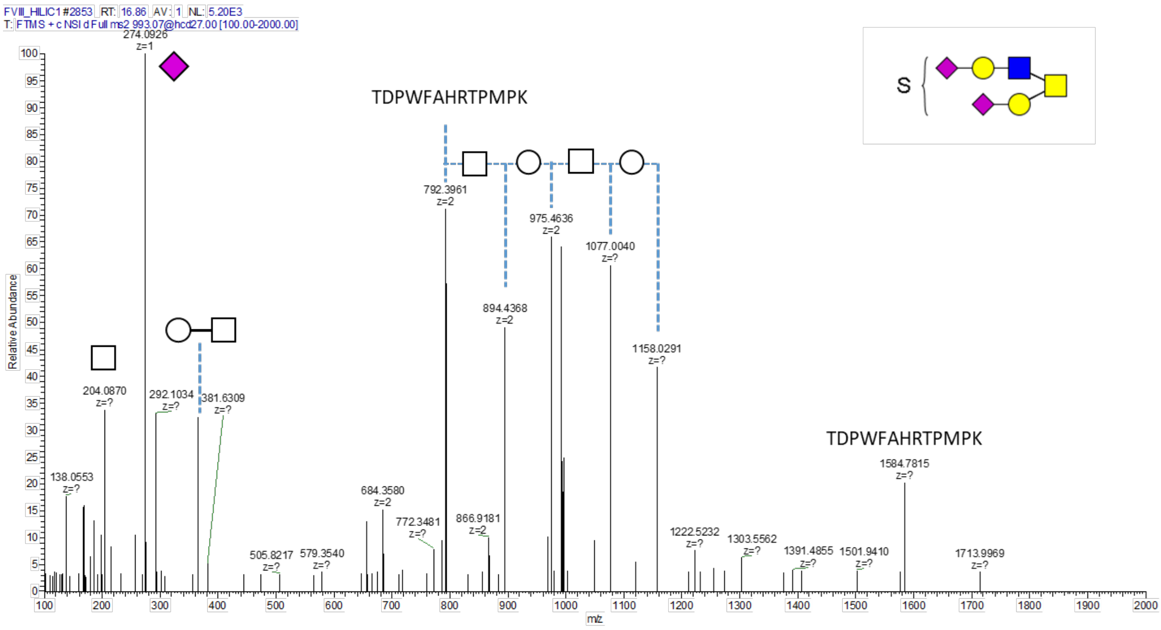

FVIII_HILIC1 #2853 RT: 16.86 AV: 1 NL: 5.20E3
T: FTMS + c NSI d Full ms2 993.07@hcd27.00 [100.00-2000.00]
TDPWFAHRTPMPK
TDPWFAHRTPMPK
S
Relative Abundance
m/z

30-O

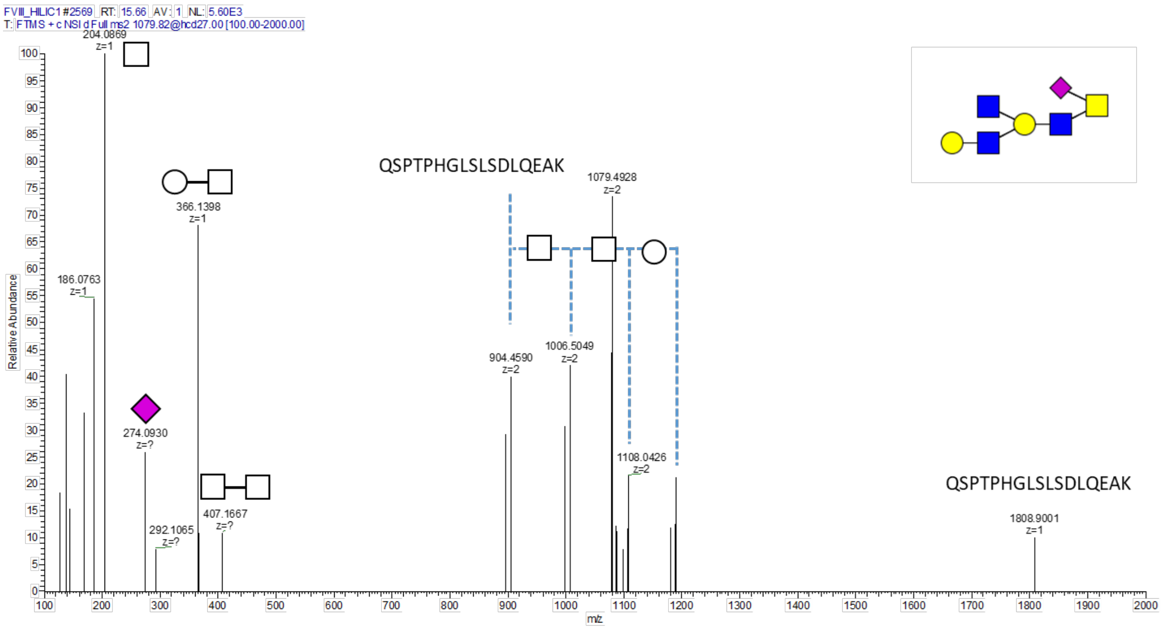
FVIII_HILIC1 #2569 RT: 15.66 AV: 1 NL: 5.60E3
T: FTMS + c NSI d Full ms2 1079.82@hcd27.00 [100.00-2000.00]
QSPTPHGLSLSDLQEAK
204.0869 z=1
366.1398 z=1
186.0763 z=1
274.0930 z=?
292.1065 z=?
407.1667 z=?
904.4590 z=2
1006.5049 z=2
1079.4928 z=2
1108.0426 z=2
1808.9001 z=1
Relative Abundance
m/z

31-O

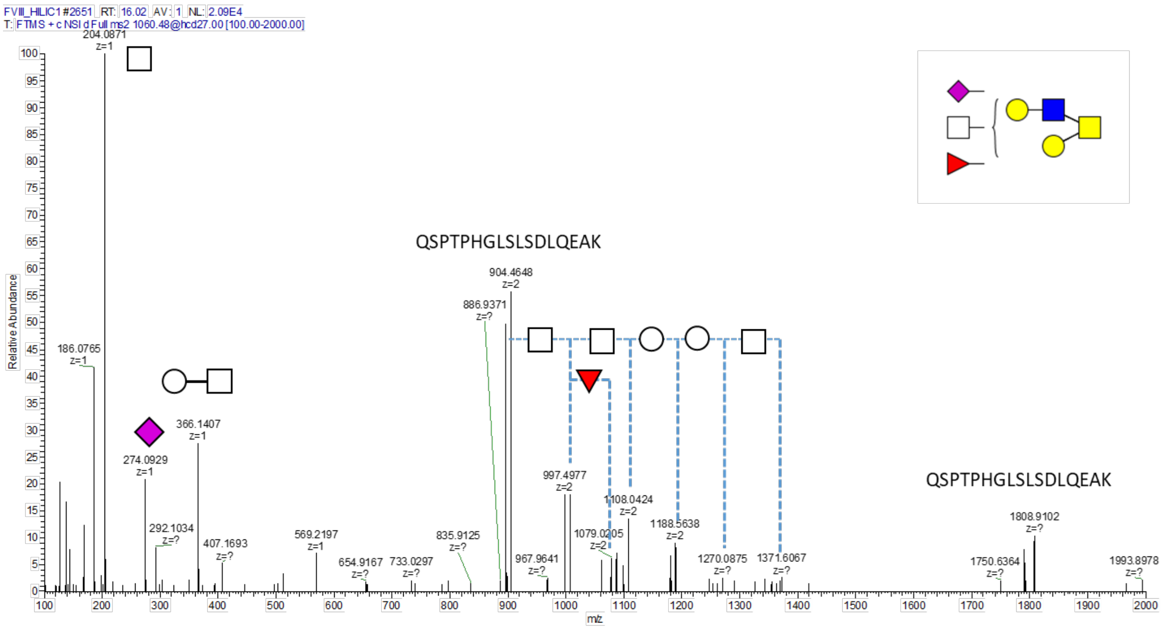
FVIII_HILIC1 #2651 RT: 16.02 AV: 1 NL: 2.09E4
T: FTMS + c NSI d Full ms2 1060.48@hcd27.00 [100.00-2000.00]
QSPTPHGLSLSDLQEAK
204.0871 z=1
186.0765 z=1
366.1407 z=1
274.0929 z=1
292.1034 z=?
407.1693 z=?
569.2197 z=1
654.9167 z=?
733.0297 z=?
835.9125 z=?
886.9371 z=?
904.4648 z=2
967.9641 z=?
997.4977 z=2
1079.0205 z=2
1108.0424 z=2
1188.5638 z=2
1270.0875 z=?
1371.6067 z=?
1750.6364 z=?
1808.9102 z=?
1993.8978 z=?
Relative Abundance
m/z
